# Transcriptional gene fusions via targeted integration at safe harbors for high transgene expression in *Chlamydomonas reinhardtii*

**DOI:** 10.1111/nph.70368

**Published:** 2025-07-08

**Authors:** Nick Jacobebbinghaus, Florian Bigge, Merve Saudhof, Wolfgang Hübner, Olaf Kruse, Thomas Baier

**Affiliations:** ^1^ Algae Biotechnology and Bioenergy, Faculty of Biology Center for Biotechnology (CeBiTec), Bielefeld University Universitätsstrasse 27 33615 Bielefeld Germany; ^2^ Biomolecular Photonics Faculty of Physics, Bielefeld University Universitätsstrasse 25 33615 Bielefeld Germany

**Keywords:** *Chlamydomonas reinhardtii*, CRISPR/Cas9, microalga, nuclear transgene engineering, targeted integration, transgene design

## Abstract

- Conventional genetic engineering in green microalgae employs error‐prone nonhomologous end joining to integrate recombinant DNA at double‐strand breaks generated at random positions across the nuclear genome. This typically results in variable transcription strength and requires a labor‐intensive screening procedure to identify transformants with sufficient expression. Current advances in genome editing enable scar‐less integration of DNA at any desired locus for engineered bioproduction.
- We optimized construct design for predictable transgene expression at a high level, significantly improved scar‐less integration rates into the nuclear genome via homology arm length optimization and quantified endogenous gene expression *in vivo*. Subsequently, endogenous genes were successfully targeted via Clustered Regularly Interspaced Short Palindromic Repeats and CRISPR‐associated protein 9 (CRISPR/Cas9) to evaluate their capacity for high transgene expression and terpenoid production.
- Highest scar‐less homology‐directed repair efficiency was achieved with 50 bp homology arms. The Light harvesting Chl a/b binding protein of LHCII (LHCBM1) locus was found to be differentially expressed under several light intensities and allows an 8.6‐fold increase in transgenic protein accumulation compared with random insertion approaches. Co‐expression of a functional sesquiterpene synthase achieved a 60‐fold increase in valencene production compared with previous attempts.
- We showed LHCBM1 locus constitutes a genetic safe harbor for transgene expression and demonstrates the potential of *C. reinhardtii* as a green cell factory.

## Introduction

The unicellular green microalga *Chlamydomonas reinhardtii* is an established model organism for fundamental research in photosynthesis, cilia biogenesis and phototaxis and has emerged as a powerful chassis for biotechnological applications. Due to the ease of phototrophic cultivation and the high carbon flux through the 2‐C‐methyl‐D‐erythritol 4‐phosphate (MEP) pathway, efficient and sustainable bioproduction of several high‐value terpenoids has been successfully demonstrated, including the ketocarotenoid astaxanthin (Amendola *et al*., 2023; Kneip *et al*., 2024), the fragrance molecules sclareol (Einhaus *et al*., 2022), patchoulol (Lauersen *et al*., 2016; Abdallah *et al*., 2022) and synthetic agarwood (Gutiérrez *et al*., 2024), as well as the chemical precursors isoprene (Yahya *et al*., 2023) and bisabolene (Wichmann *et al*., 2018). Successful transformation of the nuclear genome has already been demonstrated more than three decades ago (Kindle, 1990), and current protocols reliably allow the generation of sufficient quantities of transformants. Successive advancements in strain development (Neupert *et al*., 2009; Kurniasih *et al*., 2016), DNA assembly (Rasala *et al*., 2014; Lauersen *et al*., 2015; Crozet *et al*., 2018), promoter engineering (Scranton *et al*., 2016; Einhaus *et al*., 2021; Torres‐Tiji *et al*., 2024), reporter design (Fuhrmann *et al*., 1999; Yang *et al*., 2019; de Carpentier *et al*., 2020; Freudenberg *et al*., 2022; Gutiérrez *et al*., 2022) and coding‐sequence optimization (Barahimipour *et al*., 2015; Baier *et al*., 2018, 2020; Weiner *et al*., 2018) have distinctly improved the capacity for strong transgene expression and increased achievable titers of respective proteins and products. Despite these valuable advancements, identifying well‐performing transformants still remains a tedious challenge due to great variations in transgene expression strength within a transformant population. In detail, integration of recombinant DNA occurs at double‐strand breaks (DSB) generated at random positions across the nuclear genome via nonhomologous end joining (NHEJ) (Puchta, 2005; Plecenikova *et al*., 2014; Ferenczi *et al*., 2021). Each integration site is subject to individual epigenetic regulation, resulting in the positioning effect (Zhang *et al*., 2014; Schroda, 2019; Schroda & Remacle, 2022). In most transformants, various transgene silencing mechanisms effectively inhibit transgene expression; wherefore, large transformant populations need to be screened to identify individuals with strong expression (Wu‐Scharf *et al*., 2000; Neupert *et al*., 2020). In addition, multiple integrations (Shahar *et al*., 2020) and extensive genome rearrangements (Jinkerson & Jonikas, 2015) are frequent during nuclear transformations, which interfere with endogenous metabolism and complicate precise genetic engineering. Identifying genetic safe harbors for strong and reliable transgene expression would directly address these issues and might lead to a valuable advancement and novel tool for genetic engineering within this host.

Recent developments in CRISPR‐mediated genome editing allow precise and efficient introduction of DSB at desired positions (Shin *et al*., 2016; Angstenberger *et al*., 2020; Ferenczi *et al*., 2021; Freudenberg *et al*., 2022; Nievergelt *et al*., 2023) and novel strategies tune the homologous recombination rates for directed integration of DNA into the nuclear genome (e.g. by application of nitrogen starvation (Freudenberg *et al*., 2022) or cell synchronization (Angstenberger *et al*., 2020)). Sophisticated approaches, such as reporter tagging by fusing fluorescent reporters to endogenous genes, now allow *in vivo* protein characterization in this host (Greiner *et al*., 2017; Nievergelt *et al*., 2023). For these applications, the use of preassembled ribonucleoproteins (RNP) has successfully been established, which reduces the risk of undesired chromosomal changes or off‐target effects and displays a less invasive strategy since the RNP is subject to degradation after successful genome editing.

In Nannochloropsis, a CRISPR‐mediated, targeted integration approach was established at genomic loci exhibiting high transcriptional activity (Ryu *et al*., 2021; Südfeld *et al*., 2022). These transcriptional ‘safe harbors’ allow strong transgene expression for biotechnological application, circumvent screening efforts from random integrations and work minimally invasive to retain the genome architecture and genetic regulation. In *C. reinhardtii*, however, efficient homologous recombination and the scar‐less integration of donor DNA still remains challenging and requires further optimization. The addition of terminal DNA, homologous to the target locus (homology arms (HAs)), has been shown to assist in successful homologous recombination (Ferenczi *et al*., 2021). Currently, different guidelines for the design of donor DNA exist, which recommend HAs with a length of 0 bp (Kim *et al*., 2020; Song *et al*., 2020), 45–50 bp (Picariello *et al*., 2020; Ferenczi *et al*., 2021; Nievergelt *et al*., 2023), 500 bp (Freudenberg *et al*., 2022) or up to 2000 bp (Angstenberger *et al*., 2020); however, the optimum for efficient homology‐directed repair (HDR) by double‐stranded DNA has not been described, yet.

Aspiring to improve genetic engineering within *C. reinhardtii*, our work aimed to identify potential safe harbors for transgene expression in this alga and to improve the precision of editing for targeted integrations of transgenic DNA. In a first systematic screening, we identified the optimum for homology arm length to enhance the efficiency of scar‐less donor DNA integrations. Subsequently, we tested five genomic loci for integration of a reporter construct composed of a fluorescence reporter and a selection marker. The nuclear‐encoded genes for Cytosolic 80S ribosomal protein S8 (*RPS8*), Cytosolic 80S ribosomal protein L3 (*RPL3*) and Cytosolic 80S ribosomal protein L10A (*RPL10A*) code for ribosomal subunits, allow the assembly of the translation machinery (Ban *et al*., 2014) and are constitutively expressed at a high level. The Ribulose‐1,5‐bisphosphate carboxylase/oxygenase small subunit 2 (*RBCS2*) is part of RuBisCO, which allows inorganic carbon fixation (Cleland *et al*., 1998; Roy & Andrews, 2000) and is estimated to be the most abundant protein on earth (Ellis, 1979). The *C. reinhardtii* Light harvesting Chl a/b binding protein of LHCII (*LHCBM1*) is part of the Photosystem II light harvesting antenna system (Ferrante *et al*., 2012) and allows thermal dissipation of excess light energy via energy‐dependent quenching (Elrad *et al*., 2002). These gene targets are highly expressed under vegetative conditions (Schmollinger *et al*., 2014) and likely depict a favorable genetic framework for the integration of transgenes. We successfully performed *in vivo* characterization of target proteins, identified light dependent expression of LHCBM1 and demonstrated biotechnological application by expression of an industrially relevant sesquiterpene synthase.

## Materials and Methods

### PCR, cloning and vector design

Coding sequences were designed as optimized algal transgenes through codon optimization and integration of synthetic introns (Baier *et al*., 2018, 2020) using the open‐access software ‘Intronserter’ (Jaeger *et al*., 2019). All genetic parts were *de novo* assembled using complementary oligonucleotides, amplified by PCR from *Chlamydomonas reinhardtii* genomic DNA (Q5 High‐Fidelity DNA Polymerase; New England Biolabs (NEB, Frankfurt am Main, Germany); Supporting Information Table S1) or commercially synthesized (Genscript). The optimized *Callitropsis nootkatensis* Valencene synthase (*Cn*Vs, GenBank ID: AFN21429.1) was kindly provided by K. Lauersen (Gutiérrez *et al*., 2024). For random insertion events, the AßSAP(i) promotor (Einhaus *et al*., 2021) was used to drive Gene of Interest (GOI) expression (Notes S1, S2).

Plasmid assembly was performed based on the standardized *C. reinhardtii* modular cloning system (MoClo; Crozet *et al*., 2018). Briefly, digestion was performed using suitable restriction enzymes (BsaI, BbsI and NEB), followed by product separation in 2% (w/v) agarose gels, DNA extraction (peqGOLD Gel Extraction Kit; VWR) and ligation (T4‐Ligase; NEB) following the manufacturer's instructions. Ligation products were used for heat shock transformation of chemically competent *Escherichia coli* DH5α cells with subsequent selection at 37°C on antibiotic‐containing lysogeny broth (LB) agar plates (50 mg L^−1^ kanamycin, 300 mg l^−1^ ampicillin or 50 mg l^−1^ streptomycin). Plasmid DNA was isolated from liquid cultures after cultivation overnight (peqGOLD Plasmid Miniprep Kit I; VWR) following the manufacturer's instructions, and sequence identity was confirmed by Sanger sequencing (Center for Biotechnology, Bielefeld University).

### *Chlamydomonas reinhardtii* cultivation and transformation

All *C. reinhardtii* cell lines were routinely maintained on Tris‐Acetate‐Phosphate (TAP) agar plates (Gorman & Levine, 1965) at room temperature and 350 μmol photons m^−2^ s^−1^ continuous light. Liquid cultivations were performed in microtiter plates or Erlenmeyer flasks on an orbital shaker using TAP medium and continuous illumination at 350 μmol photons m^−2^ s^−1^. *Chlamydomonas reinhardtii* UVM4 (Neupert *et al*., 2009) was used as a parental strain for nuclear transformations via glass bead method (Kindle, 1990) or electroporation for CRISPR/Cas9‐mediated genome editing.

### CRISPR/Cas9 mediated targeted integration

Target genes for GOI co‐expression were selected based on ranked ‘Reads Per Kilobase per Million mapped reads’ values under replete cultivation conditions indicating high expression during the exponential growth phase (Schmollinger *et al*., 2014). In this study, the *C. reinhardtii* gene loci *RPS8* (Phytozome gene identifier: Cre06.g272800), *RPL3* (Cre10.g417700), *RPL10A* (Cre02.g101350), *RBCS2* (Cre02.g120150) and *LHCBM1* (Cre01.g066917) were targeted for integration of GOI expression cassette at the end of the respective coding sequence (CDS).

At least two potential sgRNA binding sites for each target gene were identified using the online tool ‘chopchop’ (https://chopchop.cbu.uib.no/; standard settings, *C. reinhardtii* genome assembly v.5.6, mismatch restriction (Labun *et al*., 2019)), and sgRNAs were synthesized using EnGen® sgRNA Synthesis Kit (NEB) followed by purification using RNA Clean & Concentrator™ (Zymo Research, Freiburg, Germany) according to the manufacturer's instructions. Cleavage efficiency was tested on PCR products from genomic DNA amplification (300–500 bp length) carrying the respective sgRNA binding site (Table S1). Functional RNP assembly was performed (0.9 pmol EnGen Cas9 protein (NEB) and 0.9 pmol sgRNA), and 0.09 pmol of PCR product were subjected to *in vitro* digest at 37°C for 30 min followed by denaturation at 80°C for 10 min and product separation via agarose gel electrophoresis (Fig. S1).

For *in vivo* genome editing, *C. reinhardtii* cells were kept in the mid‐logarithmic growth phase for 48 h followed by 24 h nitrogen starvation (nitrogen‐depleted TAP, TAP‐N). 7 × 10^7^ cells were harvested by centrifugation (1000 ***g***, 3 min), resuspended in 1 ml TAP‐sucrose (TAP medium containing 40 mM sucrose), centrifuged and resuspended in 120 μl TAP‐sucrose. A heat shock at 40°C was applied for 20 min before the addition of preassembled RNPs (8 μg EnGen Cas9 protein and 7 μg sgRNA, incubated at room temperature for 15 min) and 750 ng restriction‐enzyme‐linearized repair plasmid for HDR. Electroporation was performed using 2‐mm electrode gap cuvettes (Bio‐Rad Laboratories) and Gene Pulser Xcel System (Bio‐Rad Laboratories (Feldkirchen, Germany); square‐wave protocol, single pulse, 8 ms, 250 V; Freudenberg *et al*., 2022). After 10 min at room temperature, cells were regenerated in 5 ml TAP‐sucrose for 24 h at 5 μmol photons m^−2^ s^−1^. The selection was applied by transfer on TAP agar plates containing appropriate antibiotics (10 mg l^−1^ hygromycin or 200 mg l^−1^ spectinomycin) for 3 d at 10 μmol photons m^−2^ s^−1^ and 4 d at 500 μmol photons m^−2^ s^−1^.

**Fig. 1:**
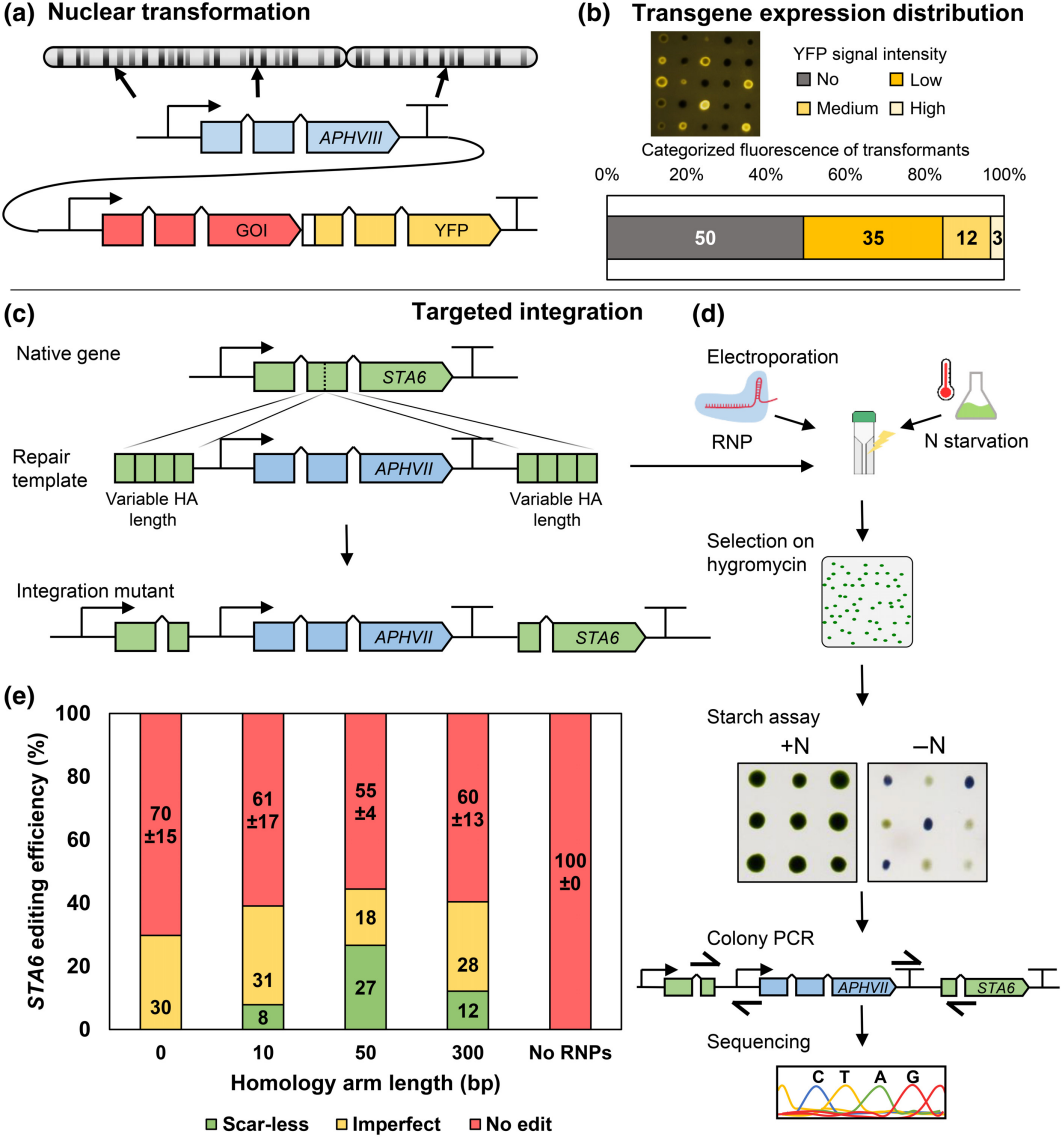
Optimized length of homology arms (HAs). (a) Schematic construct for random integration of transgenes in the nuclear genome. The selection marker is flanked by HSP70A/Ribulose‐1,5‐bisphosphate carboxylase/oxygenase small subunit 2 (RBCS2) fusion promoter and RBCS2 terminator. The Gene of Interest (GOI) *Cn*Vs is fused to a GSGSGS‐linker and the mVenus fluorescent marker, and flanked by AßAP(i) promoter and FDX1 terminator. (b) Expression pattern of randomly isolated transformants using the conventional nuclear glass bead method. After transformation recovery, 288 randomly isolated transformants are subjected to fresh Tris‐Acetate‐Phosphate (TAP) plates and analyzed via the plant fluorescence imaging system. (c) Schematics of the endogenous *STA6* gene, repair template consisting of the *APHVII* hygromycin resistance cassette with variable lengths of respective HAs, as well as the resulting mutant harboring the donor DNA for disruption of *STA6*. (d) Workflow. Pretreated *Chlamydomonas reinhardtii* cells, Cas9 ribonucleoproteins (RNPs) and repair templates were used for transformation via electroporation. After recovery and selection, the starch assay is performed (*n* = 3× 142 for each construct). Starch‐less mutants (*n* = 10) were analyzed by colony PCR and respective PCR product sequencing. (e) Editing frequencies are extrapolated respective to the recovered transformants and displayed for different lengths of HAs. Green bars show scar‐less integration of the *STA6* repair template through homology‐directed repair (HDR), while yellow bars show incorrect or no HDR for at least one of the HAs. Red bars indicate no edit in *STA6*. Error bars represent SD of technical triplicates of the starch assay.

**Fig. 2:**
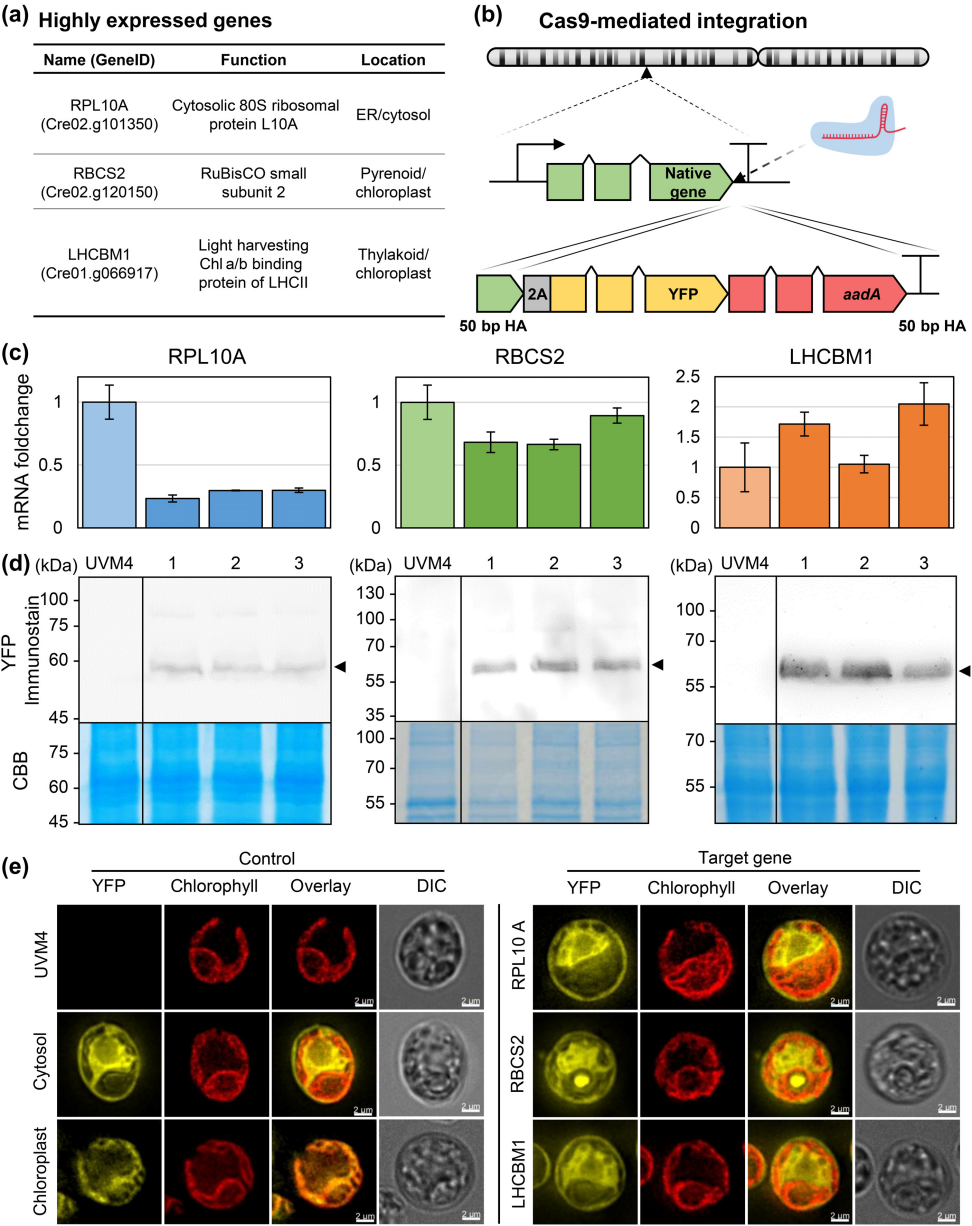
Effect of transgene fusion on native expression. (a) Selection of natively high expressed genes (Schmollinger *et al*., 2014). (b) Schematic of a highly expressed gene locus. Repair template integrated in native coding sequence and is composed of 5′‐homology arm, the FMDV 2A peptide, mVenus and aadA fusion CDS and 3′‐homology arm. (c) Relative mRNA fold change of respective gene for biological triplicates compared with UVM4. Error bars (SD) were calculated from technical triplicates. PCR products were separated in agarose gels (Supporting Information Fig. S5). (d) Western blot with immunodetection (anti‐GFP for YFP‐aadA fusion protein) and Coomassie Brilliant Blue (CBB) as loading control. (e) Single‐cell microscopy of *Chlamydomonas reinhardtii* showing YFP emission, Chl emission, a combined overlay as well as a differential interference contrast (DIC) picture for UVM4, cytosolic and chloroplast control (PSAD), representative RPL10, RBCS,2 and Light harvesting Chl a/b binding protein of LHCII (LHCBM1) transformants. GFP, green fluorescent protein; YFP, yellow fluorescent protein.

Repair templates carried the *APHVII* (Fig. 1c) or GOI expression cassette (Figs 2b, 4b) flanked by homologous DNA to the respective integration site (HAs; Notes S1). For GOI co‐expression as fusions with the target gene, the upstream HAs were designed to exclude the target gene stop codon (Fig. S2). Constructs included subsequent fusion to Glycine‐Serine‐linker (GS‐linker, 6 aa length) and the sequence of the extended FMDV2A peptide (40 aa, Plucinak *et al*., 2015) followed by yellow fluorescent protein (YFP, mVenus) or *Cn*Vs, respectively, a second GS‐linker and the selection marker aadA (including a stop codon, Figs 2b, 4b). The downstream HAs were designed complementary to the native 3′ UTR. Native and edited loci can be found in Notes S2 with the corresponding sgRNA, HAs and genetic features. The repair template for targeting ADP‐glucose pyrophosphorylase (*AGP4/STA6*, Phytozome gene identifier: Cre03.g188250) was designed previously (Freudenberg *et al*., 2022).

### Starch assay

*STA6* targeted transformants were tested for starch accumulation as described previously (Work *et al*., 2010). Colonies were transferred to nitrogen‐depleted TAP medium and cultivated for 2 d at 500 μmol photons m^−2^ s^−1^. Colonies were exposed to sublimated iodine for 10 min. The ratio of edited (green phenotype) to unedited cells (dark coloration) was quantified.

### Genomic DNA amplification

Selected transformants were grown in 10 ml TAP until stationary phase, and genomic DNA extraction was performed using the NucleoSpin Microbial DNA Kit (Macherey‐Nagel, Düren, Germany) according to the manufacturer's instructions. PCR was performed using Q5 polymerase (NEB) and target‐specific oligonucleotides adjacent to the HAs (Table S1). PCR products were separated via agarose gel electrophoresis, purified using the peqGOLD Gel Extraction Kit (VWR), and the sequence identity for scar‐less integration was verified by Sanger sequencing.

### Fluorescence measurements and single‐cell microscopy

Transformants with a repair template containing mVenus were selected by YFP fluorescence using the Leica Binocular MZFLIII system (LSM780; Carl Zeiss GmbH, Oberkochen, Germany) equipped with suitable filters (excitation at 514 nm and emission at 520–550 nm).

Transformants were individually cultivated in 24‐well microtiter plates under 0, 15, 120 and 350 μmol photons m^−2^ s^−1^ for 4 d, and quantitative expression intensities were determined routinely using a microtiter plate reader (Tecan Group Ltd, Männedorf, Switzerland) based on fluorescence signals normalized to the respective cell densities. For single‐cell microscopy, a 10 μl cell suspension from the early logarithmic growth phase was mounted between a microscope slide and a high precision #1.5 coverglass (Marienfeld‐Superior, Germany). Images were recorded on a Deltavision Elite microscope (GE Healthcare, Düsseldorf, Germany) with a 60 × 1.42 N.A. oil immersion PlanApoN objective (Olympus Life Science, Hamburg, Germany) and an Evolve 512 EMCCD camera (Photometrics, Tucson, AZ, USA). The following excitation and emission wavelengths were used to record YFP and Chl signals: YFP excitation at 505–522 nm and emission at 537–559 nm, and Chl excitation at 563–588 nm and emission at 603–648 nm. A single reference white‐light differential interference contrast (DIC) image and several Z planes were recorded at a distance of 250 nm. The fluorescent images were deconvolved with the appropriate recorded optical transfer function at default parameters for 10 iterations in the softWoRx v.6.1.3 program (GE Healthcare). The background was averaged on deconvolved images with a Gaussian blur at 1 px width (Fiji/ImageJ package). The intensities were adjusted linearly: The background was subtracted with a value determined by the nonexpressing controls for each channel, whereas the maximum intensities were individually adjusted for representation. The images shown represent a single deconvolution Z‐slice of a stack in the correct cell orientation.

Two hundred eighty‐eight randomly isolated random insertion transformants were characterized by fluorescence measurements using the plant imaging system NightShade LB985 (Berthold Technologies, Bad Wildbad, Germany) with respective YFP‐fluorescence filter sets (excitation: 504/10 nm, emission: 530/20 nm). Fluorescence intensity was grouped into four categories: no rfu corresponds to no expression, 0–22 000 rfu to low expression, 22 000–44 000 rfu to medium expression and > 44 000 rfu to high expression. Twenty transformants with the highest rfu values were subjected to terpenoid production cultivation.

### RNA extraction and RT‐qPCR

Three transformants for each locus were individually cultivated in 45‐ml TAP medium until the mid‐logarithmic growth phase. Equal amounts were harvested by centrifugation (3000 ***g***, 3 min), and total RNA was extracted via Quick‐RNA MiniPrep Kit (Zymo Research) according to the manufacturer's instructions. Reverse transcriptase quantitative polymerase chain reaction (RT‐qPCR) was conducted using Luna® Universal One‐Step RT‐qPCR Kit (NEB) and 200 ng total RNA according to the manufacturer's instructions. Target mRNA amounts were quantified with specific oligonucleotides: 18S RNA (for 5′‐ACCTGGTTGATCCTGCCAG‐3′; rev 5′‐TGATCCTTCGCAGGTTCAC‐3′), RPL10A (for 5′‐TTCTCCGCGAGAGTGTCTCC‐3′; rev 5′‐CATCACCCAGCACGCACACG‐3′), RBCS2 (for 5′‐CCAGGTCGACTACATTGTCG‐3′; rev 5′‐TCTCGCGCAGCACCTGCATG‐3′) and LHCBM1 (for 5′‐TCGACCGTCAAGGTCGAGGC‐3′; rev 5′‐CGTGGATCAGCTCCAGCTCG‐3′). SYBR Green fluorescence was recorded in a LightCycler® 96 System (Roche Diagnostics GmbH, Mannheim, Germany), and relative mRNA expression levels were determined according to the 2^−ΔΔCt^ method (Livak & Schmittgen, 2001) and compared with expression in parental strain UVM4. Amplified fragments were subjected to an agarose gel verifying specific and correct amplification in the RT‐qPCR (Fig. S3).

### SDS‐PAGE and western blotting

*Trans*formants were individually cultivated in 6‐well microtiter plates until the mid‐logarithmic growth phase. In total, 4 × 10^7^ cells were harvested by centrifugation (3000 ***g***, 3 min) and pellets were resuspended in 200 μl 2× SDS sample buffer (60 mM Tris pH 6.8, 4% (w/v) SDS, 20% (v/v) glycerol, 0.01% (w/v) bromophenol blue). Equal protein amounts (2 × 10^6^ cells) were separated in 12% polyacrylamide gels during Tris‐glycine‐SDS‐PAGE (Laemmli *et al*., 1970). Gels were stained using colloidal Coomassie Brilliant Blue G‐250 (Dyballa & Metzger, 2009) or were subjected to western blotting (semi‐dry method; Trans‐Blot Bio‐Rad, blotting buffer: 25 mM Tris, 192 mM glycine, 20% (v/v) methanol) on 0.45 μm Protran nitrocellulose membranes (Amersham). Overnight blocking was performed using blocking buffer (5% (w/v) BSA and 5% (w/v) milk powder in TBS) followed by immunodetection via green fluorescent protein (GFP) antibody (rabbit‐anti‐GFP antibody; Thermo Scientific (Schwerte, Germany) (A10260), 1 : 5000 in TBST (TBS with 0.1% (v/v) Tween 20)) conjugated to horseradish peroxidase. Sequential washing steps were performed (TBS with 0.1% Tween 20) before the addition of Pierce ECL Western Blotting Substrate (Thermo Scientific) and detection in a Fusion FX Imaging system (Thermo Scientific).

### Culture‐solvent two phase cultivations and heterologous terpenoid quantification

Transformants were individually cultivated for 6 d at 350 μmol photons m^−2^ s^−1^ in 4.5‐ml TAP medium supplemented with 10% (v/v) dodecane (Sigma Aldrich) as an organic solvent overlay, as described previously (Lauersen *et al*., 2016; Wichmann *et al*., 2018). Dodecane fractions were harvested by centrifugation and separated via gas chromatography, and product amounts were quantified via flame ionization detector as described previously (Einhaus *et al*., 2021).

## Results and Discussion

### Short homology arms assist in scar‐less homology‐directed transgene integration

Expression constructs typically consist of two separate open reading frames (ORF) for (I) expression of a selectable marker and (II) for expression of a protein of interest (Fig. 1a). After integration, each ORF is subject to individual aspects of eukaryotic transcriptional regulation, which consequently results in highly variable protein accumulation in regenerated transformants. Although considerable transformation efficiencies can be achieved using current protocols (Jacobebbinghaus *et al*., 2024), present transgene silencing mechanisms reduce expression rates in most of the regenerated transformants (Fig. 1b). We tested an established protocol for nuclear nontargeted transgene insertion of *C. reinhardtii* UVM4 and obtained 347 ± 13 colonies/μg DNA (*n* = 3) without further optimization. However, up to 50 ± 5% of all regenerated transformants exhibit fluorescence below the detection limit (Fig. 1b; screening of 288 random transformants, *n* = 4), indicating sufficient expression of the selection marker *APHVIII* for survival on antibiotic‐containing media but poor expression intensity of the second ORF, likely due to silencing at the respective integration locus (e.g. position effects) and partial integration of the YFP gene. Another factor influencing transgene expression is the rearrangement and mutation of integrated DNA, which is not feasible to identify in the random insertion strategy. Only 3 ± 2% of regenerated transformants exhibit strong fluorescence signals suitable for subsequent investigation and biotechnological application (Fig. 1b). A labor‐intensive high‐throughput screening procedure is essential for the identification of these outliers with sufficient expression levels, which is a significant limitation for the use of *C. reinhardtii* as a green cell factory.

Recent developments in genome editing technology allow the precise introduction of DSB at desired positions and targeted integration of recombinant DNA (by use of donor DNA during electroporation of RNPs). Addition of terminal sequences at the donor DNA, homologous to the target locus (HA), was shown to support HDR and integration at desired positions. To allow efficient HDR, we tested different homology arm lengths for scar‐less integration. The endogenous *STA6* locus was selected for the integration of a hygromycin resistance cassette (*APHVII*; Fig. 1c) due to the ease of screening for transformants with impaired starch biosynthesis (in the presence of iodine, successful genome editing results in a green, starch‐less phenotype, while parental strains exhibit a stained, starch‐containing phenotype; Fig. 1d). The donor DNA was flanked by either no HAs or HAs with lengths ranging from 10 to 300 bp (Fig. 1c) complementary molarities for each repair template were used. The selection was performed based on resistance against hygromycin, and up to 426 individual transformants from three independent transformations were subjected to iodine vapor in a starch assay, if applicable (Fig. S4). Transformants exhibiting antibiotic resistance but intact starch biosynthesis (Fig. 1e, red column) likely contain an integration of the *APHVII* resistance cassette at random positions and an unaltered *STA6* locus. For all tested HAs, comparable editing frequencies of *c*. 30–45% of preselected transformants were observed based on starch‐less phenotypes (Fig. 1e, sum of green and yellow bars) and a respective control without the addition of Cas9 protein and sgRNA (Fig. 1e, no RNPs) did not result in any observed edit of the *STA6* locus. These results are consistent with the previous reports of equally high editing efficiencies (Freudenberg *et al*., 2022), likely due to suitable sgRNA design and efficient HDR at this locus despite the difference in genome editing protocols used.

To distinguish correct HDR‐mediated DNA integrations from NHEJ‐based repair events (e.g. small sequences insertions or deletions accompanied by *APHVII* integration at random positions), selected starch‐less mutants (*n* = 10 for each HA length) were subjected to PCR‐based sequence characterization of both junctions of the inserted DNA (Fig. S4C,D). Complete scar‐less edits without sequence alterations were identified and separated from the transformants exhibiting undesired alterations in one or both of the genomic junctions, which are classified as imperfect (Fig. 1e). This verification method displays another important advantage of targeted integration compared with random insertion and allows feasible detection of mutations and rearrangements in the transgene.

Donor DNA without HAs led to imperfect integration events, similar to previous findings using genome editing approaches without HAs (Song *et al*., 2020; Kneip *et al*., 2024). During NHEJ, donor DNA can be integrated in bidirectional orientation and is frequently processed until terminal microhomologies stabilize DNA ends, a process called microhomology‐mediated end joining (MMEJ; McVey & Lee, 2008; Pannunzio *et al*., 2018), which is typically accompanied by undesired sequence alterations at the target locus. These additional genomic changes may influence gene expression and are undesired for biotechnological application. Scar‐less integration was only observed when HAs were included, and our data suggest that their length influences the frequency of successful genome editing.

In 45 ± 4% of all antibiotic‐resistant transformants, an integration of donor DNA was observed when the length of HAs was *c*. 50 bp homologous to the target locus (green and yellow columns; 170 of 378 transformants). In 27% of resistant transformants, error‐free HDR was observed (Fig. 1e, green column) while 18% exhibited imperfect edits (Fig. 1e, yellow column). HAs of only 10 bp resulted in scar‐less integration in 8% of resistant transformants, while 31% carried imperfect integrations. It is very likely that short HAs are insufficient to mediate correct attachment of donor DNA at the correct locus or are subject to rapid degradation preventing HDR‐based repair (e.g. by endogenous exonucleases). Longer HAs (300 bp) resulted in 12% scar‐less and 28% imperfect integrations. During HDR, 5′‐ends of a Cas9‐mediated DSB undergo end processing to expose a single‐strand overhang, followed by D‐loop formation and assembly of homologous regions (Baidukova *et al*., 2023). Long complementary DNA ends are likely more prone to imperfect recombination due to alternative binding sites or steric hindrances of remaining overhangs during end processing. In addition, Ferenczi *et al*. (2021) showed that longer HAs than 45 nt (e.g. 60 and 75 nt) decreased the editing efficiency when using single stranded repair templates. In this study, the optimal length of HAs was found to be *c*. 50 bp, which was successfully applied previously (Ferenczi *et al*., 2021; Nievergelt *et al*., 2023) and allowed frequent scar‐less HDR and efficient genome editing in *C. reinhardtii*.

### Integration of designed transgenes affects endogenous transcription but allows independent translation

Genetic engineering of Chlamydomonas is currently limited by inefficient transcription of introduced transgenes (Lumbreras & Purton, 1998; Barahimipour *et al*., 2015; Einhaus *et al*., 2024). Additionally, transcription strength is dictated by the genomic integration site (Schroda, 2019) due to epigenetic regulations, for example, histone modifications or DNA methylation resulting in position effects and potentially in heterochromatin formation and transgene silencing. Current advances in genome editing enable scar‐less integration of target DNA at any desired locus and suitable positions for engineered bioproduction. We selected several potential integration sites at the C‐terminus of highly expressed endogenous genes under mixotrophic conditions (Fig. 2a) for heterologous co‐expression of a reporter construct containing the viral 2A peptide, the fluorescence reporter mVenus and the selection marker aadA (Fig. 2b). The selected target sites include three ribosomal subunit proteins (RPS8, RPL3 and RPL10A) and two proteins involved in photosynthesis (RBCS2 and LHCBM1). It is reported that transcriptionally active DNA is more accessible for Cas9‐RNP binding, which would support genome editing efforts (Jain *et al*., 2021) due to lower histone abundance and reduced DNA condensation (Nightingale *et al*., 1998; Tse *et al*., 1998; Ishii *et al*., 2024). In addition, these gene positions natively offer a suitable gene expression regulation, which can be hijacked to establish efficient transgene co‐expression. Genome integrations were performed in fusion to the C‐terminal end to avoid interference with N‐terminal sequence motifs, such as targeting peptides important for posttranslational protein transport (e.g. LHCBM1 and RBCS2 contain N‐terminal chloroplast targeting peptide (CTP) sequences) and in frame with the native CDS. However, we also included the FMDV 2A peptide for protein separation by ribosome skipping for target protein separation and maintaining their function. To confirm correct sgRNA‐mediated targeting to the desired genomic locus, an *in vitro* digest was performed using amplification products for all investigated targets. The results indicate successful DSB at all selected sgRNA binding sites and successful Cas9 activity (Fig. S1). *In vivo* genome editing was performed for integration of donor DNA at respective positions, and regenerated transformants were selected based on spectinomycin antibiotic resistance and fluorescence signals (Fig. S5A). Subsequent colony PCR and sequencing confirmed successful donor DNA integration in selected mutants (Fig. S5B). Targeting RPS8 and RPL3 did not result in any transformant exhibiting successful edits, likely due to reduced cellular fitness when modifications were made at the C‐terminus of these proteins. It is important to note that after 2A‐mediated ribosome skipping, a 39 amino acid (aa) long extension remains attached to the protein chassis. A 6 aa long spacer (GS‐linker) was included for spatial separation of this peptide extension; however, sequence additions may interfere with functional ribosome assembly and consequently hinder translation. In future attempts, alternative fusion strategies may be designed to allow co‐expression using these targets specifically. However, screening was successful for three selected genomic loci: RPL10A, RBCS2 and LHCBM1. When RPL10A was targeted, 3 of 15 preselected transformants carried the desired genotype, 5 of 20 for RBCS2 and 11 of 66 for LHCBM1 (Fig. S5C). Similar to our previous experiment, the HA length was tested on the LHCBM1 locus (10, 50, 100, 500 and 1000 bp): The highest scar‐less editing efficiency could be observed for 50 bp (2.5%), which is decreasing gradually with longer HAs (Fig. S6). Interestingly, the fluorescing transformants often showed additional integration of DNA on the downstream end of the DSB, which is in line with previous studies (Freudenberg *et al*., 2022; Kneip *et al*., 2024). All selected candidates carried scar‐less additions of 2254 bp (1674 bp on mRNA level) at the target locus with unknown effects on global genome architecture. Relative transcript levels of RPL10A and RBCS2 were strongly decreased compared with amounts in parental cell line UVM4 by 72 ± 3% and 25 ± 10%, respectively, in three independently tested mutants (Fig. 2c). By contrast, the expression of LHCBM1 was found to be increased by 61 ± 41% in selected mutants. The relative transcript abundance is likely affected by changes in endogenous regulation of promoter strength, influenced by epigenetic factors at introduced DNA, additional alterations in gene expression regulation and likely mRNA stability. The observed effects from DNA integrations emphasize that the expression of native genes may also be affected by downstream located sequences and that increased mRNA complexity is not generally associated with the downregulation of expression. Increased expression can partially result from the application of endogenous introns in donor DNA inducing intron‐mediated enhancement previously reported (Baier *et al*., 2020) or from endogenous factors upregulating expression of LHCBM1 to allow nonphotochemical quenching and efficient light harvesting (Ferrante *et al*., 2012; Liu *et al*., 2024).

**Fig. 3:**
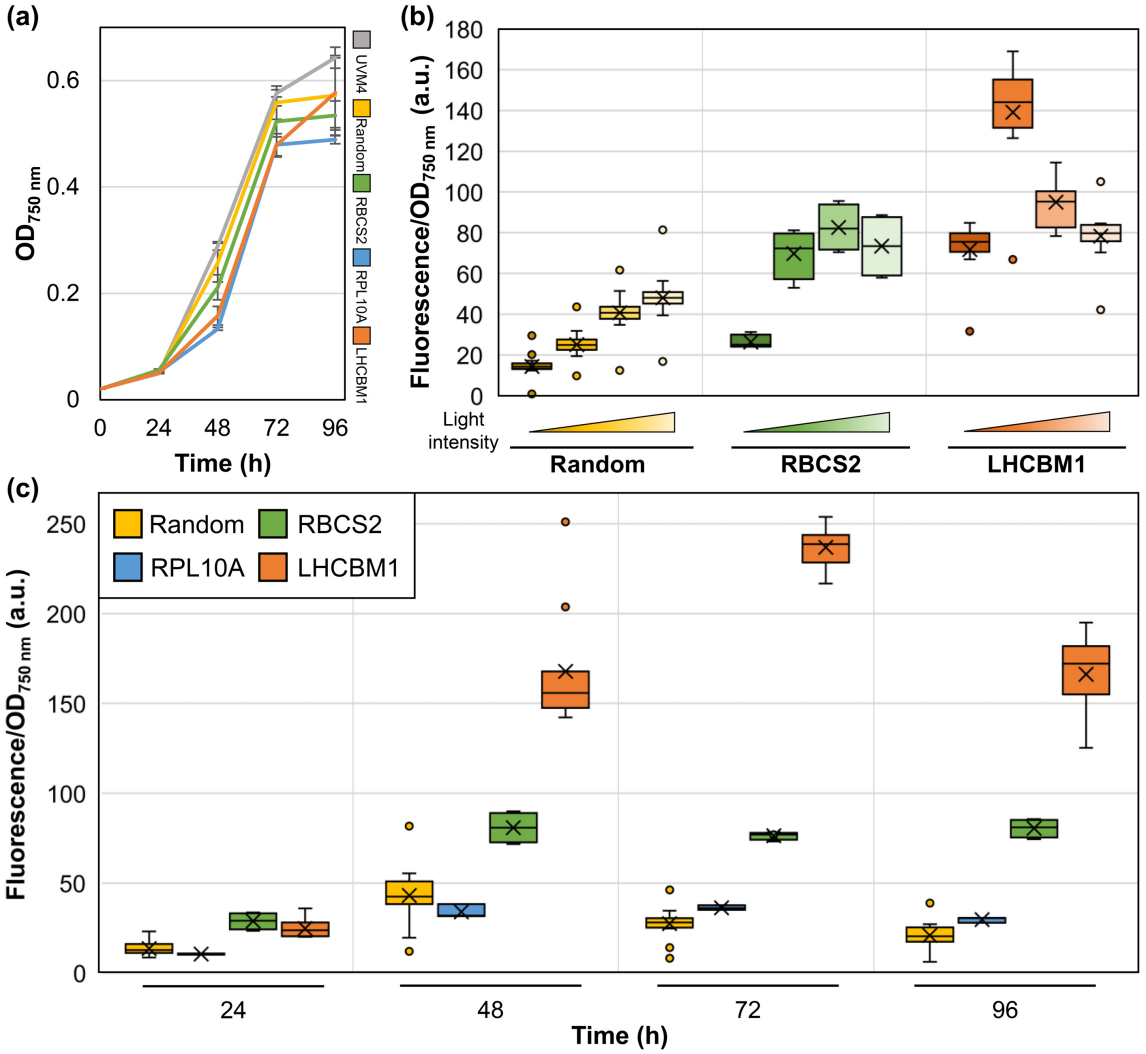
Growth and expression characterization. (a) Optical density of individual *Chlamydomonas reinhardtii* transformants for mixotrophic cultivations using Tris‐Acetate‐Phosphate (TAP) medium for 96 h at 350 μmol photons m^−2^ s^−1^. Error bars represent SD. (b) Gene expression was analyzed by Fluorescence/OD_750 nm_ measurements at different light intensities after 24 h for random integrated (*n* = 20) and targeted integration of yellow fluorescent protein (YFP) (Ribulose‐1,5‐bisphosphate carboxylase/oxygenase small subunit 2 (RBCS2) (*n* = 4), Light harvesting Chl a/b binding protein of LHCII (LHCBM1) (*n* = 11)), respectively (from dark to bright: 0, 15, 120 and 350 μmol photons m^−2^ s^−1^). (c) Fluorescence/OD_750 nm_ was measured for 96 h at 350 μmol photons m^−2^ s^−1^ for the best 20 out of 288 random integrated YFP transformants (yellow), as well as all scar‐less YFP integrated transformants for each integration site: RPL10A (blue, *n* = 3), RBCS2 (green, *n* = 4) and LHCBM1 (orange, *n* = 11). Horizontal line marks the median, the x denotes the mean, and the box displays the lower and upper quartile values. Minimum and maximum are depicted as short horizontal lines connected to the box. Outliers are represented as single data points.

Application of the FMDV 2A peptide was performed to allow ribosome skipping, consequently separating native proteins from the downstream located reporter construct (Fig. 2a,d,e) and to reduce the footprint from co‐expression during translation. Western blot signals suggest that the majority of reporter proteins are separated from the native protein and that RPL10A, RBCS2 and LHCBM1 remain independent (signals at 57 kDa, while fusion proteins would be found at *c*. 82–89 kDa; Fig. 2d). Exact cleavage efficiencies (70% for RPL10A, 89% for RBCS2 and 100% for LHCBM1; Fig. S7) are similar to previously reported efficiencies (85–90% cleavage efficiency; Donnelly *et al*., 1997, 2001; Ryan & Drew, 1994). Single‐cell fluorescence microscopy was performed to identify correct protein localization and to confirm independent protein accumulation. Control constructs were designed using a mVenus reporter without targeting peptide for cytosolic localization, while chloroplast import was induced by the application of the previously described CTP from the chloroplastic Photosystem I reaction center subunit II (PSAD gene (Fischer & Rochaix, 2001; Fig. 2e). For RPL10A targeted mutants, fluorescence was only found in the cytosol; however, these signals may overlap with remaining fusion proteins. For RBCS2 targeted mutants, fluorescence was found only in the cytosol; however, signals at the pyrenoid indicate residual protein fusions still allowing condensation into mature RuBisCO protein complexes. For LHCBM1 targeted mutants, YFP fluorescence was also detected only in the cytosol, while remaining fusion proteins would be expected in the chloroplast after posttranslational import using the native CTP. We conclude that co‐transcription of GOIs from these loci is possible in *C. reinhardtii*, that DNA integrations have diverse impacts on endogenous gene expression, and that protein separation can efficiently be designed by application of the FMDV 2A peptide, which can influence targeted proteins function or cellular fitness (Fig. 3); however, it efficiently results in independent protein accumulation.

### Enhanced transgene expression and its application in basic research

In eukaryotes, nuclear gene expression underlies several aspects of complex regulation, including diverse structural features (e.g. chromatin state) and interactions with DNA‐binding protein factors (e.g. transcription factors and enhancers; Bacova *et al*., 2020; Kronholm *et al*., 2017; Strenkert *et al*., 2022). Each gene is regulated in response to environmental, physiological or metabolic changes, but the abundance of target gene expression is difficult to investigate *in vivo* and typically quantified via quantitative PCR or western blotting at certain time points during cultivations. Protein tagging for co‐expression of fluorescence proteins fused to a native target can be performed by Cas9‐mediated integration for continuous protein characterization (Nievergelt *et al*., 2023). The complex regulation and specific genetic architecture at the respective gene loci are altered by integrating heterologous DNA, which affects transgene expression and may result in changes in cellular fitness. Growth of generated mutants was compared with parental strain UVM4, and moderate differences were detected for all generated cell lines (Fig. 3a). Under mixotrophic conditions and when cultivated in microtiter plates, *C. reinhardtii* UVM4 typically exhibits a rapid logarithmic growth phase until 72 h past inoculation and reached a final optical density of OD_750 nm_ = 0.64 ± 0.03 in this study. Transformants carrying a random integration of the reporter construct showed a slight reduction in growth (final OD_750 nm_ = 0.57 ± 0.13, −11%) compared with UVM4, which was also observed for mutants with a targeted integration site: RPL10A (OD_750 nm_ = 0.49 ± 0.02, −23%), RBCS2 (OD_750 nm_ = 0.53 ± 0.05, −17%) and LHCBM1 (OD_750 nm_ = 0.58 ± 0.11, −9%). The pronounced decrease for RPL10A is likely correlated with the low transcript abundance of this locus (Fig. 2c), indicating that this specific target is sensitive to alterations; however, no additional phenotypical changes were observed (unchanged maximum PSII yields compared with UVM4; Fig. S8). Slight reductions in final optical densities may also be correlated with genomic alteration after transformation found in all investigated cell lines, which were not further investigated. Targeted integration allows *in vivo* monitoring of expression by fluorescence measurements under various conditions and was quantified under different light conditions here. Fluorescence intensity in transformants with a random integration site correlated with applied light intensities and was highest in high‐light intensities (Fig. 3b). During cultivations in the dark, expression of RBCS2 was present; however, it was elevated by 3.1‐fold under illumination with 120 μmol photons m^−2^ s^−1^. Higher light intensities (350 μmol photons m^−2^ s^−1^) led to a 2.8‐fold increase in expression compared with expression levels in the dark. RuBisCO is involved in carbon fixation in the Calvin–Benson–Bassham (CBB) cycle and is highly expressed in the dark, as well as during the light phase (Goldschmidt‐Clermont & Rahire, 1986), and our results suggest that gene expression may be fine‐tuned by the applied light intensity during mixotrophic cultivations.

For LHCBM1 targeted mutants, relatively high fluorescence levels were observed, which were found to be maximal under low‐light conditions (15 μmol photons m^−2^ s^−1^) and decreased under high light (350 μmol photons m^−2^ s^−1^, Fig. 3b). These results are similar to reports indicating that LHCBM1 expression is typically downregulated under high‐light intensities to balance the abundance of light harvesting complexes and photoprotection (Anderson *et al*., 1988; Bailey *et al*., 2001).

Conventional genetic engineering in *C. reinhardtii* often results in low expression rates and efficient time‐dependent silencing in selected transformants (Schroda, 2019; Neupert *et al*., 2020). Direct fusion of transgenes to native genes circumvents low expression rates and leads to high and predictable gene expression. The expression dynamics at selected loci were quantified by fluorescence measurements over the course of a cultivation at 350 μmol photons m^−2^ s^−1^ (Fig. 3c). Expression was associated with the exponential growth phase and decreased after 48 h past inoculation. Mean YFP expression from the RBCS2 locus was increased up to 2.8‐fold after 72 h of cultivation compared with transformants with a random integration site. Highest expression was obtained for LHCBM1 mutants, with an 8.6‐fold increase in expression after 72 h. Fluorescence measurements were repeated after 18 months and no silencing could be detected (Fig. S9). Since the applied UVM4 strain already shows reduced transgene silencing, the diminished silencing could also be attributed to the inactive Sir2‐type histone deacetylase, SRTA (Neupert *et al*., 2020). However, additional factors acting on transgene silencing are still active in this strain. To verify that the extinguished silencing effect can be ascribed to the transgene fusion to endogenous genes, similar experiments should be conducted in wild‐type strains in a future study. This would also increase the robustness of the strain in larger cultivations, as UVM4 inherits a reduced cell wall. However, these observations demonstrate the large expression capacity from RBCS2 and LHCBM1 loci and depict them as safe harbors for high transgene expression.

### Application for efficient terpenoid bioproduction

**Fig. 4:**
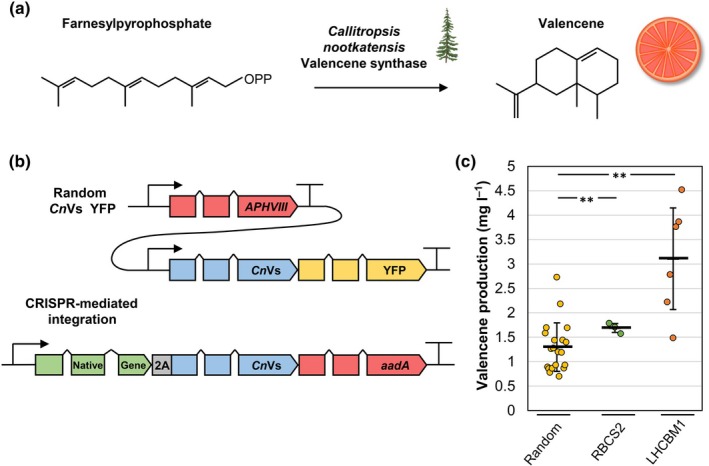
Biotechnological approach. (a) Reaction from Farnesylpyrophosphate (FPP; originating from methylerythritol phosphate pathway) to valencene through the *Callitropsis nootkatensis* Valencene synthase. (b) Schematic constructs for random insertion and scar‐less targeted integration of the *Cn*Vs in between native coding sequence and 3′‐UTR. (c) Valencene production by *Chlamydomonas reinhardtii* is presented for best random integrated (*n* = 20) and scar‐less integrated *Cn*Vs after Ribulose‐1,5‐bisphosphate carboxylase/oxygenase small subunit 2 (RBCS2) (*n* = 3) and Light harvesting Chl a/b binding protein of LHCII (LHCBM1) native loci (*n* = 6). For random integrated *Cn*Vs, best 20 expressing transformants were chosen based on yellow fluorescent protein (YFP) fluorescence out of 288 random isolated transformants. − displays mean production of each group and error bars represent the SD. Asterisks (*) indicate the significance level of an unpaired, two‐tail Students *t*‐test for mean production assuming unequal variances (**, *P* < 0.01).

Green microalgae are suitable hosts for sustainable bioproduction of high‐value terpenoids due to their efficient carbon flux through the MEP pathway; however, heterologous expression of functional terpene synthases from the nuclear genome remains challenging and is a key bottleneck in their application (Einhaus *et al*., 2022). We selected the previously described *Callitropsis nootkatensis* Valencene synthase (*Cn*Vs (Beekwilder *et al*., 2014)) to investigate strategies for optimized expression and tested targeted integration at suitable positions for efficient co‐expression and establishment of valencene biosynthesis, the native precursor of the aroma compound nootkatone (Fig. 4a). Conventional expression strategies (Fig. 4b) employ fusions of the CDS with fluorescence reporters and DNA integrations at random positions, which require labor‐intensive and inefficient screening for transformants with high expression. We identified the best 20 candidates from a transformant population with random integration sites and quantified volumetric valencene production according to established protocols (Einhaus *et al*., 2021; Fig. 4c). Similar to previous efforts (Lauersen *et al*., 2016; Wichmann *et al*., 2018; Einhaus *et al*., 2022), a large variation in terpenoid production was observed for selected transformants, which typically correlates with respective expression intensity (Lauersen *et al*., 2016). Valencene production was found to be within the range of 0.7–2.7 mg l^−1^ (mean 1.3 mg l^−1^) after 6 d in mixotrophic conditions. In a second approach, we successfully established integration and co‐expression of the *Cn*Vs at the C‐terminus of RBCS2, which resulted in volumetric valencene production of 1.7 ± 0.1 mg l^−1^ and a significant 1.3‐fold increase compared with transformants with random integrations. Production was further increased by using the integration site at the LHCBM1 C‐terminus and present endogenous expression regulations. Respective mutants showed a mean production of 3.1 ± 1.0 mg l^−1^ (2.4‐fold increase). Interestingly, relatively high expression was observed for a single outlier with a random genomic integration site (2.7 mg l^−1^); however, maximal production was observed for a candidate with integration at the LHCBM1 locus (4.5 mg l^−1^). The variation in valencene production rates ranging from 1.5 to 4.5 mg l^−1^ cannot be affiliated with the corresponding *Cn*Vs mRNA expression rates, which is equal in between the biological replicates (Fig. S10). In addition, nanopore sequencing identified a single integration at the LHCBM1 locus for three individual transformants (Figs S11–S13). We conclude that the LHCBM1 locus is in a suitable position to establish heterologous gene expression; however, the nuclear genome may contain additional, yet undescribed loci suitable for high transgene expression. Consequently, this work was able to demonstrate fine‐tuned genetic engineering and achieved a 60‐fold increase in valencene production compared with previous attempts (0.052 mg l^−1^ (Gutiérrez *et al*., 2024)) and similar yields compared with individual *Cn*Vs overexpression in *Saccharomyces cerevisiae* (Beekwilder *et al*., 2014). The targeted integration concept offers an alternative engineering strategy, which allows controlled integration of DNA without off‐target effects and will assist in the investigation of present regulation at specific gene positions across the nuclear genome.

## Competing interests

None declared.

## Author contributions

NJ, OK and TB contributed to the conceptualization. NJ, WH and TB contributed to the methodology. NJ, FB, MS and WH contributed to the investigation. NJ and TB contributed to the writing – original draft. NJ, FB, MS, WH, OK and TB contributed to the writing – review and editing. OK contributed to the funding acquisition.

## Disclaimer

The New Phytologist Foundation remains neutral with regard to jurisdictional claims in maps and in any institutional affiliations.

## Supporting information


**Fig. S1** Results for Cas9 *in vitro* digest for tested loci.
**Fig. S2** Schematic repair template design.
**Fig. S3** Amplified fragments for RNA quantification via RT‐qPCR.
**Fig. S4** Optimized length of homology arms screening process.
**Fig. S5** Target loci screening.
**Fig. S6** Homology arm length effect on LHCBM1 locus.
**Fig. S7** Cleavage efficiency of 2A peptide.
**Fig. S8** Pulse amplitude modulated fluorometry measurement.
**Fig. S9** Long‐term silencing.
**Fig. S10** *Cn*Vs expression on target loci.
**Fig. S11** Nanopore sequencing results.
**Fig. S12** Nanopore sequencing results.
**Fig. S13** Nanopore sequencing results.
**Notes S1** Expression vectors for nuclear transformation of *Chlamydomonas reinhardtii*.
**Notes S2** Native and edited genes.

**Table S1** Oligonucleotides.

**Table S2** Main figure data.
Please note: Wiley is not responsible for the content or functionality of any Supporting Information supplied by the authors. Any queries (other than missing material) should be directed to the *New Phytologist* Central Office.

## Data Availability

The data that support the findings of this study are available in the Supporting Information of this article (Notes S1, S2, Figs S1–S13 and Tables S1, S2).
