# Supplementary material for: Transcriptional gene fusions via targeted integration at safe harbors for high transgene expression in Chlamydomonas reinhardtii
Source: New Phytol. 2025 Jul 8;247(6):2665–77. doi: 10.1111/nph.70368 (PMC12371178; doi:10.1111/nph.70368)
Supplement: Supplementary file 1 — Fig. S1 Results for Cas9 in vitro digest for tested loci. Fig. S2 Schematic repair template design. Fig. S3 Amplified fragments for RNA quantification via RT‐qPCR. Fig. S4 Optimized length of homology arms screening process. Fig. S5 Target loci screening. Fig. S6 Homology arm length effect on LHCBM1 locus. Fig. S7 Cleavage efficiency of 2A peptide. Fig. S8 Pulse amplitude modulated fluorometry measurement. Fig. S9 Long‐term silencing. Fig. S10 CnVs expression on target loci. Fig. S11 Nanopore sequencing results. Fig. S12 Nanopore sequencing results. Fig. S13 Nanopore sequencing results. Notes S1 Expression vectors for nuclear transformation of Chlamydomonas reinhardtii. Notes S2 Native and edited genes. [file NPH-247-2665-s001.docx]

## *New Phytologist* Supporting Information

Article title: **Transcriptional gene fusions via targeted integration at safe harbors for high transgene expression in *Chlamydomonas reinhardtii***

Authors: Nick Jacobebbinghaus, Florian Bigge, Merve Saudhof, Wolfgang Hübner, Olaf Kruse, Thomas Baier

Article acceptance date: 19 June 2025

The following Supporting Information is available for this article:

**Fig. S1** **Results for Cas9 *In Vitro* Digest for tested loci**

**Fig. S2** **Schematic repair template design**

**Fig. S3** **Amplified fragments for RNA quantification via RTqPCR**

**Fig. S4** **Optimized length of homology arms screening process**

**Fig. S5** **Target loci screening**

**Fig. S6** **Homology arm length effect on LHCBM1 locus**

**Fig. S7** **Cleavage efficiency of 2A peptide**

**Fig. S8** **PAM measurement**

**Fig. S9** **Longterm silencing**

**Fig. S10** ***Cn*Vs expression on target loci**

**Fig. S11** **Nanopore sequencing results**

**Fig. S12** **Nanopore sequencing results**

**Fig. S13** **Nanopore sequencing results**

**Table S1 Oligonucleotides**

**Table S2 Main figure data**

**Notes S1** **Expression vectors for nuclear transformation of *Chlamydomonas reinhardtii***

**Notes S2** **Native and edited genes**

**Fig. S1 Figure S1 Results for Cas9 *In Vitro* Digest for tested loci**. (**A**) The targets with the amplified full PCR fragment [bp] and the expected Cas9 restricted fragments after *in vitro* digest are displayed for each sgRNA, respectively. Agarose gels are displayed with the amplified PCR product and the respective fragments for the *in vitro* digests for (**B**) RBCS2 (digested products are 476 bp and 266 bp (sgRNA 1), 466 bp and 276 bp (sgRNA 2), 454 bp and 288 bp (sgRNA 3) and 478 bp and 264 bp (sgRNA 4)), (**C**) for LHCBM1 (digested products are 332 bp and 281 bp (sgRNA 1) and 385 bp and 228 bp (sgRNA 2)), (**D**) for RPS8 (digested products are 149 bp and 391 bp (sgRNA 1) and 152 bp and 388 bp (sgRNA 2)), (**E**) for RPL10A (digested products are 156 bp and 426 bp (sgRNA 1), 210 bp and 372 bp (sgRNA 2) and 209 bp and 373 bp (sgRNA 3)) and (**F**) for RPL3 (digested products are 215 bp and 331 bp (sgRNA 1), 235 bp and 311 bp (sgRNA 2) and 227 bp and 319 bp (sgRNA 3)).

**
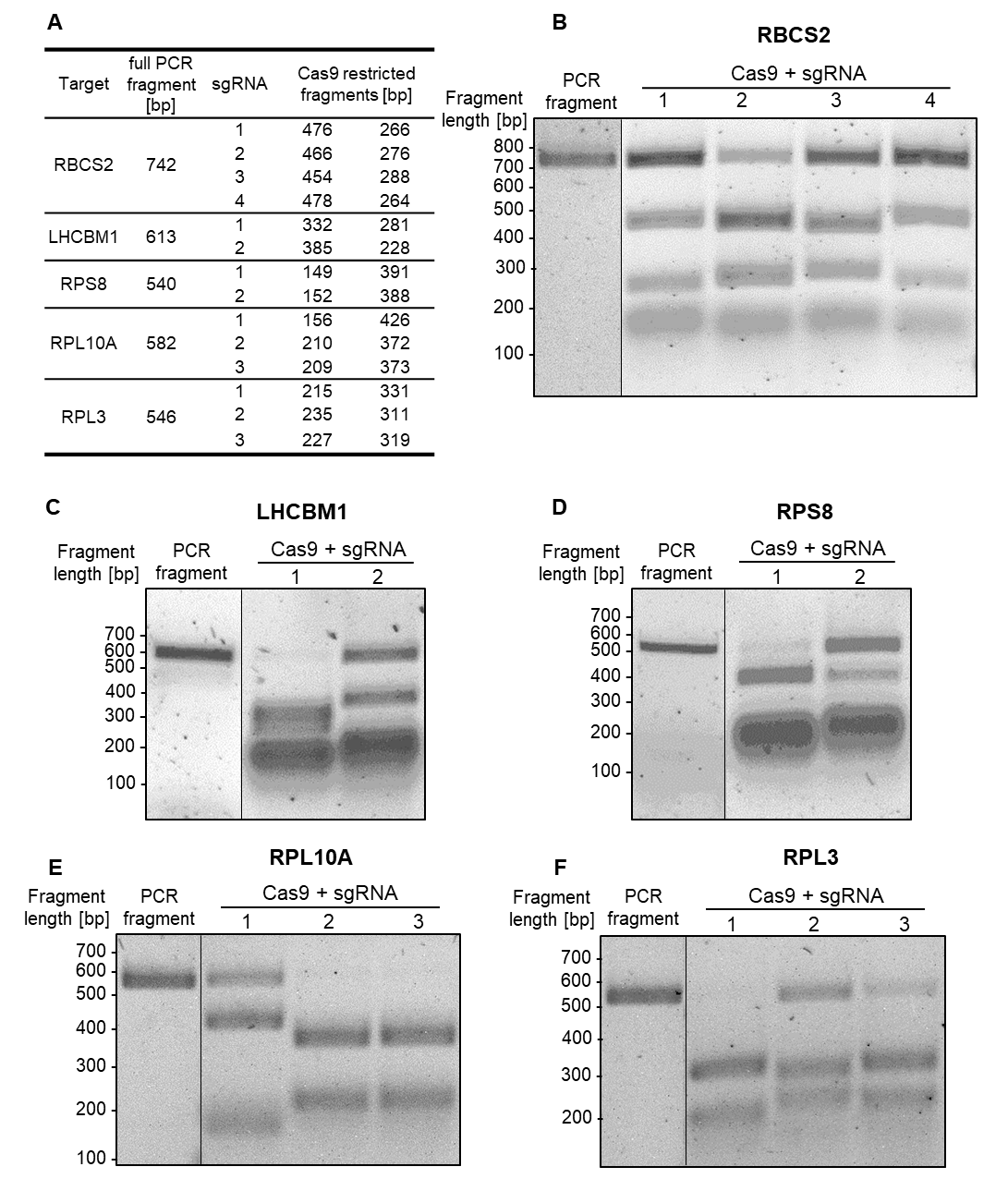
**

**Fig. S2 Schematic repair template design.** (**A**) The native gene is depicted with schematic promotor (squared arrow), exons (green boxes), introns (carets) and terminator (T) as well as start (ATG) and stop codon (TAA). Dashed vertical line symbolizes the predicted DSB (double strand break) caused by the Cas9 RNP (light blue shape). (**B**) For designing the repair template HAs are chosen as follows: 50 bp upstream of the DSB as the upstream HA and 50 bp from the 5’-end of the terminator. The repair template starts with the upstream HA and is followed by the remaining 3’-end of the coding sequence but without the stop codon to ensure readthrough during translation. The next element in the repair template the 2A peptide for protein separation flanked by two GS-linkers. The YFP (located downstream of the 2A peptide) is connecting the aadA (spectinomycin resistance) through another GS-linker ending with the stop codon (TAA). The last element of the repair template is the downstream HA. (**C**) A schematic procedure of the RNP caused DSB in the native gene with the homology directed repair through the (**i**) YFP repair template and (**ii**) *Cn*Vs repair template is displayed. Homologous parts from the repair template to the native gene are marked with two vertical lines. The native gene is then scar-lessly connected to the 2A peptide, either the YFP or *Cn*Vs, and the *aadA*.

**
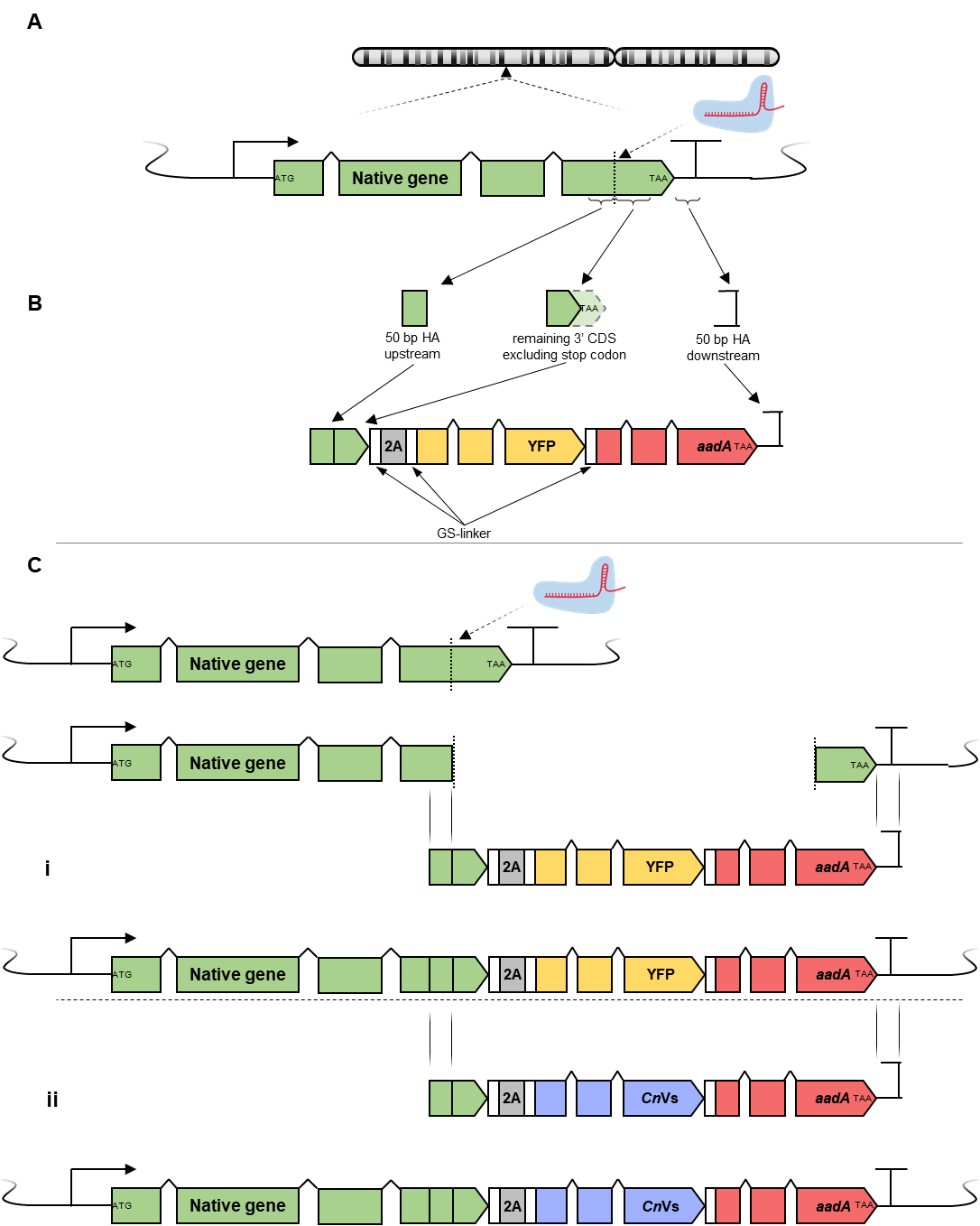
**

**Fig. S3 Amplified fragments for RNA quantification via RTqPCR**. (**A**) RTqPCR products are shown for (**i**) LHCBM1 (oligo for 5’-TCGACCGTCAAGGTCGAGGC-3’ and rev 5’-CGTGGATCAGCTCCAGCTCG-3’) resulting in 229 bp amplified DNA fragment, (**ii**) RBCS2 (oligo for 5’-CCAGGTCGACTACATTGTCG-3’ and rev 5’-TCTCGCGCAGCACCTGCATG-3’) resulting in 194 bp amplified DNA fragment and (**iii**) RPL10A (oligo for 5’-TTCTCCGCGAGAGTGTCTCC-3’ and rev: 5’-CATCACCCAGCACGCACACG-3’) resulting in 186 bp amplified DNA fragment for three individual transformants with altered respective native gene. Controls were UVM4 as positive control and H_2_O and –RT (no reverse transcriptase) as negative controls. (**B**) Housekeeping gene was 18s RNA (oligo for 5’-ACCTGGTTGATCCTGCCAG-3’ and rev 5’-TGATCCTTCGCAGGTTCAC-3’) applied for all transformants as well as the controls. (**C**) Native length and mRNA length with insertion is shown for each target loci.

**
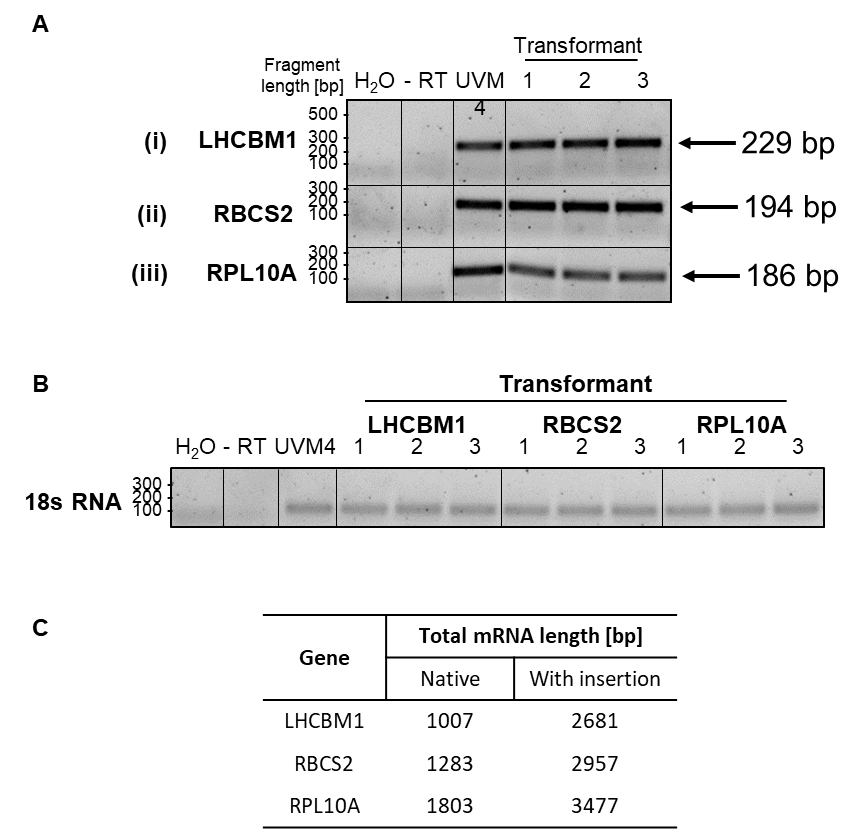
**

**Fig. S4 Optimized length of homology arms screening process**. (**A**) Editing efficiencies (unspecific and scar-less edit) with integration of APHVII Hygromycin resistance cassette in STA6 gene for respective homology arm lengths. For each transformation round (3 rounds) 142 transformants (if applicable) were submitted to the starch assay. Standard deviation (SDV) was calculated for each starch assay round. (**B**) Calculation of the proportion of perfect edited transformants. (**C**) Starch assay results: Nitrogen starved mutants were incubated with iodine for 10 min. Dark stained transformants contain starch and have no STA6 edit, while unstained transformants show STA6 edit. Starch-less mutants are selected for colony PCR. (**C**) Colony PCR was performed for 50 bp homology arm construct obtained transformants for (**i**) upstream homology arm (STA6 oligo for 5’-ATTTGATGCTTCCGGACTCGTCGATGTGACCGTCCCGCCATCTCG-3’ and PSAD oligo rev 5’-TTGTTGAGGCATGCTGAGAGCGCCTGGG-3’ resulting in a fragment of 336 bp and (**ii**) downstream homology arm (FDX1 oligo for 5’-GGGGAGCACCACTGTGAGCAGTTGC-3’ and STA6 oligo rev 5’-ATCTTGGTGACATTGCTGTTCAGGCAGTTGCTAACGGGAATATCG-3’) resulting in a fragment of 350 bp, if the repair mechanism homology directed repair was flawless. (**D**) Colony PCR was performed for 300 bp homology arm construct obtained transformants for (**i**) upstream homology arm (STA6 oligo for 5’-CAGTGGTGCGCAAGGCCGTGAGC-3’ and PSAD oligo rev 5’-GTTGTTGAGGCATGCTGAGAGCGCCTGGG-3’ resulting in a fragment of 595 bp and (**ii**) downstream homology arm (FDX1 oligo for 5’-GGGGAGCACCACTGTGAGCAGTTGC-3’ and STA6 oligo rev 5’-GCAGCCCCGCCCTTGTGTCCCTTCC-3’) resulting in a fragment of 619 bp, if the repair mechanism homology directed repair was flawless.

**
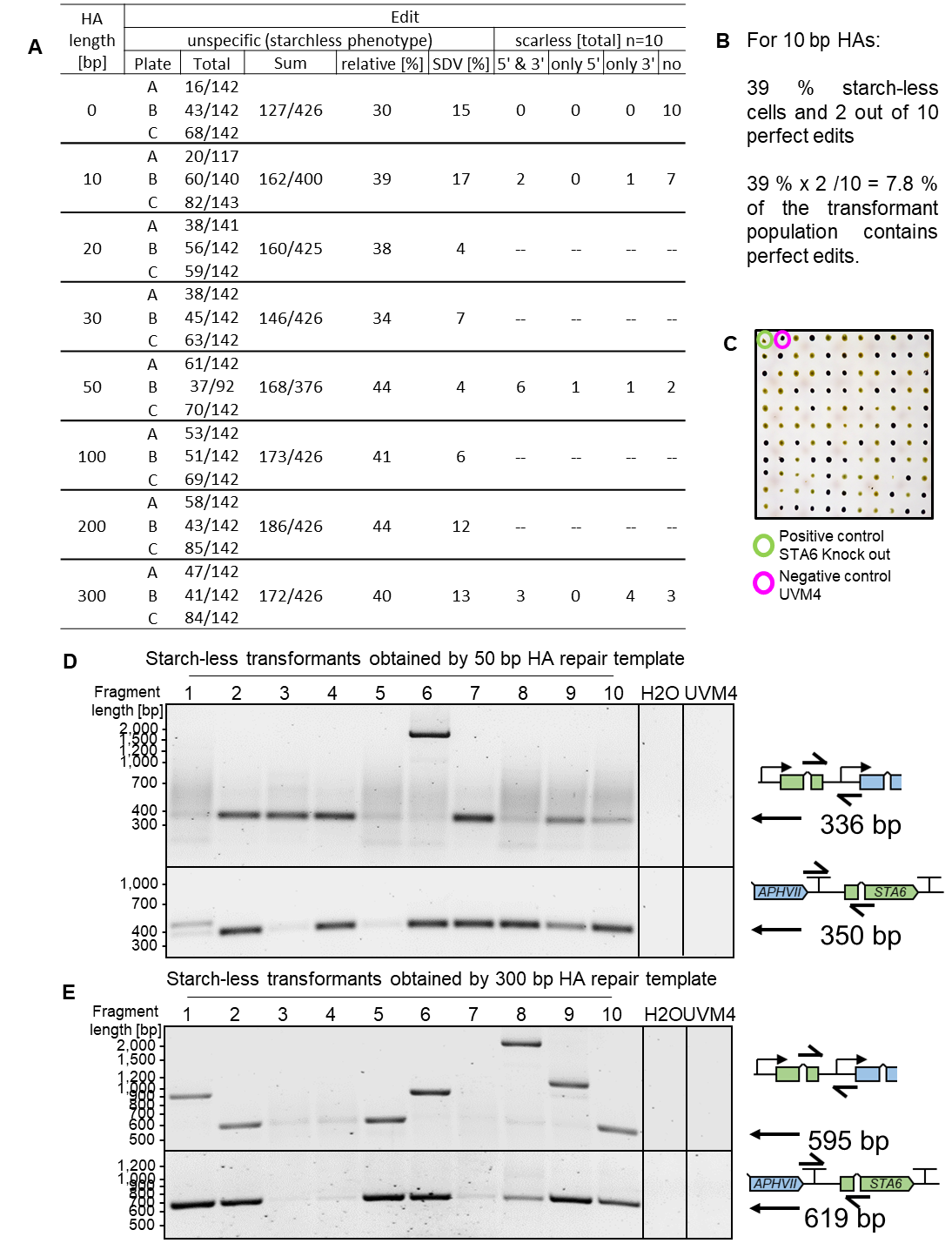
**

**Fig. S5 Target loci screening.** Analysis is shown for LHCBM1 as a representative. All target loci were screened using this procedure. (**A**) Transformation plate targeting LHCBM1 after recovery phase of 10 days under light (left) and with YFP excitation at 504/10 nm nm and emission at 530/20 nm (right). (**B**) Results for colony PCR using different primer pairs: Flanking upstream Homology arm: LHCBM1 for (5’-GTGGCTCGTTCGACCCCCTGGGCCTGGC-3’) and YFP rev (5’-CCACGCCGGTGAACAGCTCC-3’) resulting in 589 bp amplified DNA fragment. Flanking downstream homology arm: AADA for (5’-GTGATCCTGGAGGCCCGCCAGGCCTACCTG-3’) and LHCBM1 3’UTR rev (5’-GTCTTCTCTCAAAATTTACAAACGTTGACAAGTCCCAGCGCAGC-3’) resulting in 337 bp amplified DNA fragment. Flanking whole edit: LHCBM1 for (5’-GTGGCTCGTTCGACCCCCTGGGCCTGGC-3’) and LHCBM1 3’UTR rev (5’-GTCTTCTCTCAAAATTTACAAACGTTGACAAGTCCCAGCGCAGC-3’) resulting in 2867 bp amplified DNA fragment (UVM4 control 613 bp fragment). (**C**) Transformation efficiency and corresponding editing efficiencies for integration of YFP_AADA insert in between coding sequence and 3’UTR for respective loci are depicted as absolute counts and the respective ratio to obtained transformants in clamps. 50 bp HAs were flanking the repair template.

**
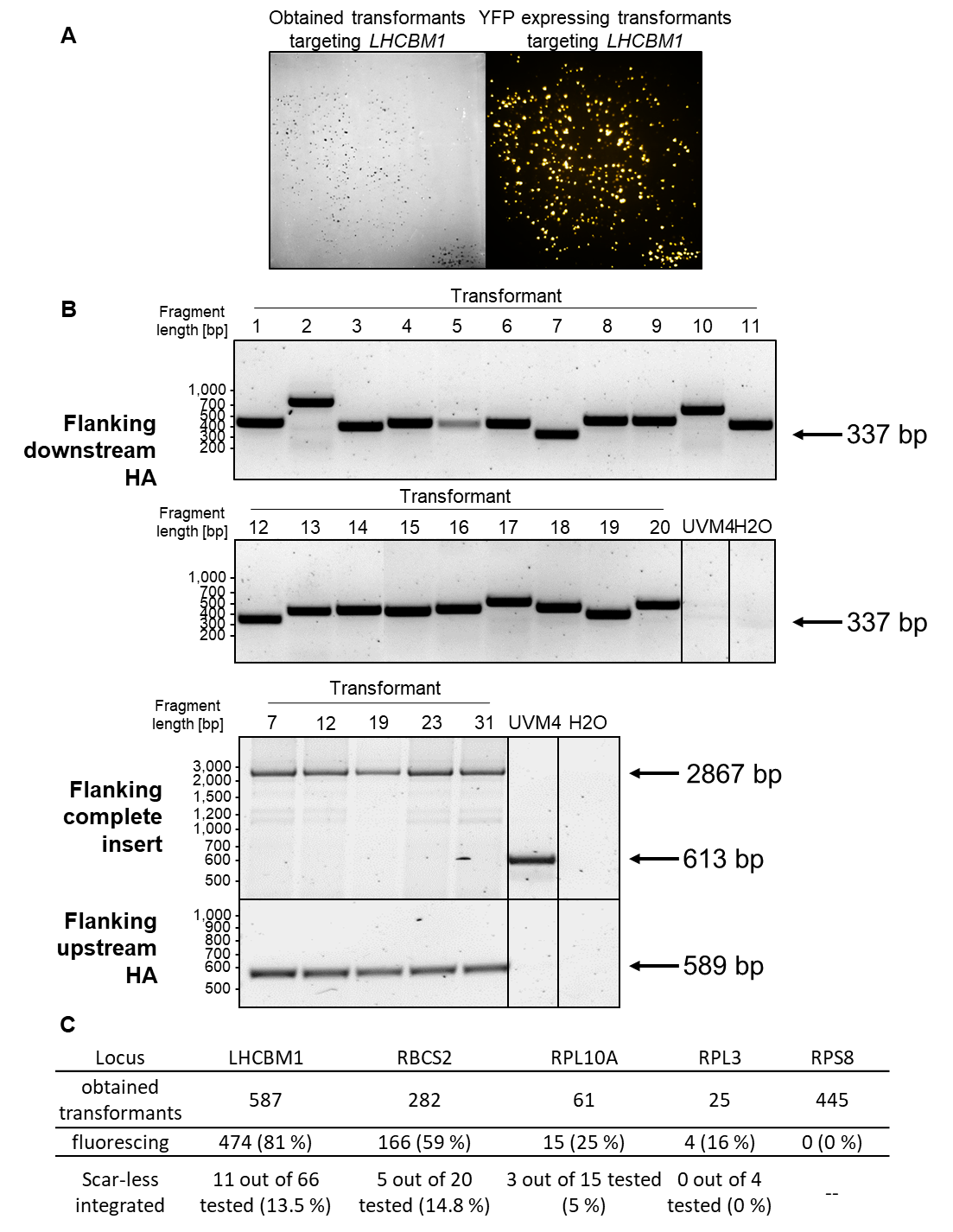
**

**Fig. S6 Homology arm length effect on LHCBM1 locus**. The repair template shown in Figure 2B was used to edit the LHCBM1 locus with altering homology arm lengths (10 bp, 50 bp, 100 bp, 500 bp and 1000 bp). (**A**) Similar to the the STA6 locus regenerated transformants were counted and compared to the amount of fluorescing transformants. Fluorescing transformants were subjected to colony PCR and subsequent sequencing to identify scar-less edited transformants. (**B**) Calculations of the scar-less edited transformants were extrapolated onto the whole transformant population. (**C**) The editing efficiency for scarred (fluorescing) transformants and scar-less edited transformants are displayed for the respective HA lengths extrapolated for the whole transformant population. The results show the highest scar-less editing efficiency for 50 bp HAs although the most fluorescing transformants could be detected for 100 bp HAs. The scar-less editing efficiency is gradually decreasing from 2.5 % (50 bp HA) over 1.8 % and 1.1 % (100 bp and 500 bp HAs) to no scar-less edited transformants (1000 bp HA). Also shorter HAs than 50 bp (10 bp) resulted in no scar-less transformants. Together with the results from the STA6 locus and previous work (Ferenczi et al., 2021) this confirms 50 bp HAs are enhancing scar-less editing efficiencies.

**
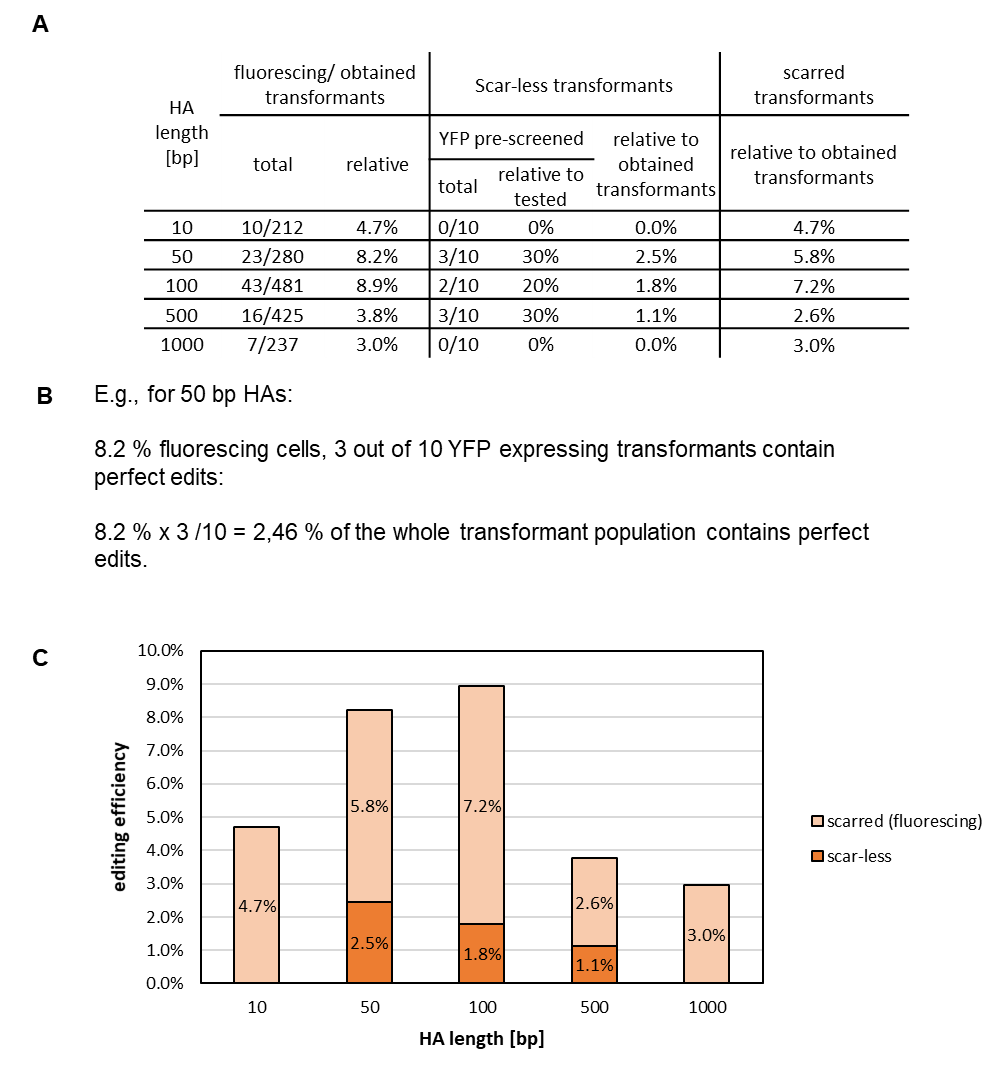
**

**Fig. S7 Cleavage efficiency of 2A peptide.** Cleavage efficiency of 2A peptide was analyzed for different loci on the basis of the signal intensity observed via immunodetection (Figure 2). ImagJ was used to determine signal intensities for each transformant. Mean relative efficiencies from biological triplicates are displayed for the RPL10A, RBCS2 and LHCBM1 loci with corresponding SDV.

**
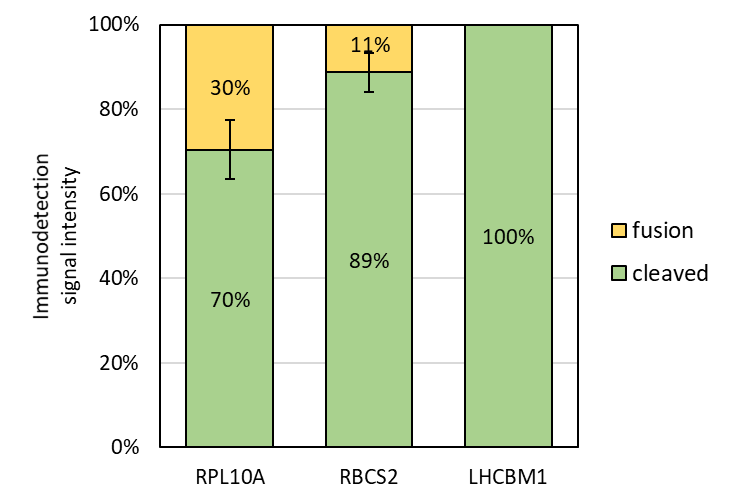
**

**Fig. S8 Pulse amplitude modulated (PAM) fluorometry measurement.** F_v_/F_m_ values were measured using the FluorCam 800MF (PSI (Photon Systems Instruments), Drásov, Czech Republic). Individual data points for each 2A-YPF-aadA transformant were summarized in box blots. Cells were cultivated in HSM media (Sueoka, 1960) at 100 µmol photons m^-2^ s^-1^ for 48 h and 3% CO_2_ supply. Subsequently, cells were incubated in the dark for 1 h until measuring maximum PSII yield (F_v_/F_m_). Results show, values for parental strain UVM4 is lower than for all transformants. This indicates, transformants are not stressed in these conditions and genetic alterations have no negative effect on the maximum PSII yield.

**
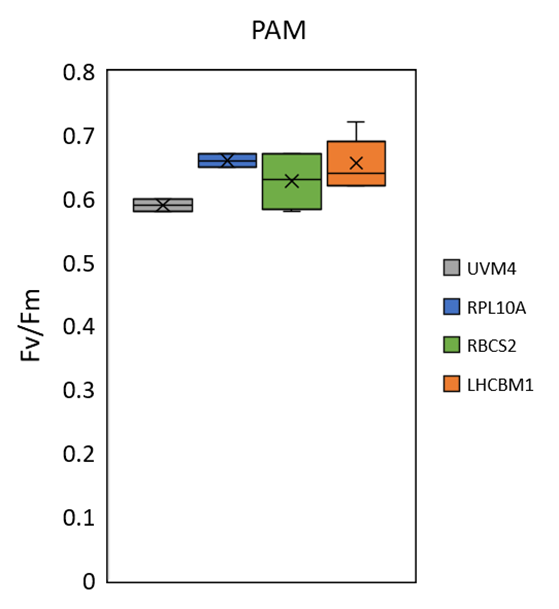
**

**Fig. S9 Longterm silencing**. Random YFP transformants as well as targeted YFP transformants were analyzed by fluorescence measurements after 48 h of cultivation after 1 month after regeneration from the transformation plate. The transformants were transferred on fresh TAP agar plates with spectinomycin weekly and the fluorescence measurement was repeated 18 months after regeneration from the transformation plate to see if the YFP expression is at the same level. The fluorescence data shows only lowered fluorescence levels for the different transformants of LHCBM1. However, light intensities and growth phase play an enormous role for the targeted expression as shown in Figure 3B and 3C. Therefore, reductions in fluorescence can be pointed e.g., to the time point of the measurement.

**
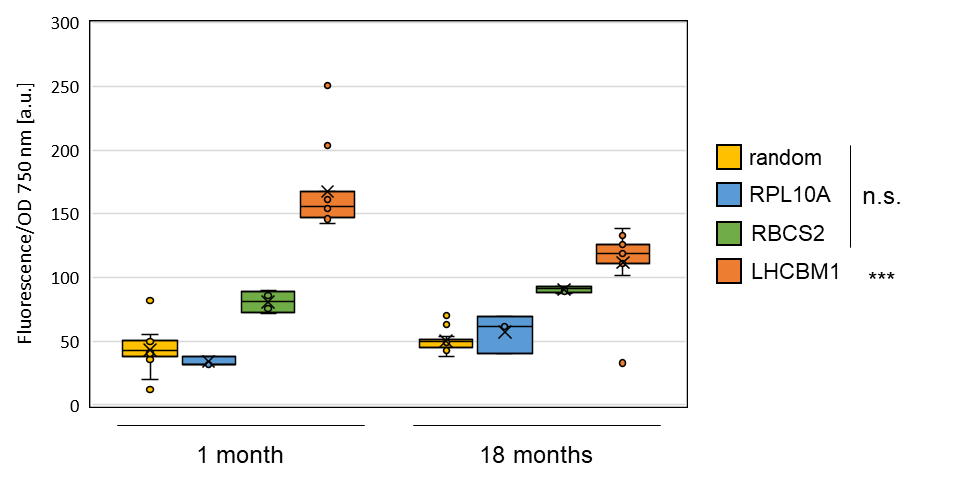
**

**Fig. S10 *Cn*Vs expression on target loci**. RNA was isolated from LHCBM1 and RBCS2 targeted *Cn*Vs transformants and RTqPCR was performed with oligonucleotides binding to *Cn*Vs (oligo for 5’-GCGAGGAGGGCCAGTTCAGC-3’ and oligo reverse 5’-GTCGCCGTACTCCTTGATCACGTGC-3’) with the housekeeping gene RPL13 (oligo for 5’GGCCAGACCCTGAAGTACAA-3’ and oligo reverse 5’-GCTCTCCAGCGACTTGTTG-3’) as expression control. The results show no alterations in expression of *Cn*Vs within the RBCS2 expression lines as well as the LHCBM1 expression lines.

**
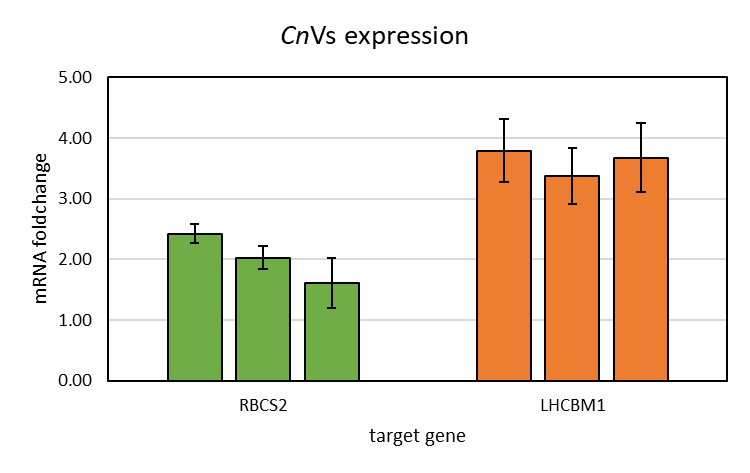
**

**Fig. S11 Nanopore sequencing results.** Whole genome nanopore sequencing was performed for LHCBM1 targeted expression strains expressing the *Cn*Vs and reads with oligos inside the insert were mapped on the *C. reinhardtii* genome (v5.6). Insertion was only found in the desired position (3’end of the LHCBM1 coding sequence) highlighted on the annotated LHCBM1 gene.

**
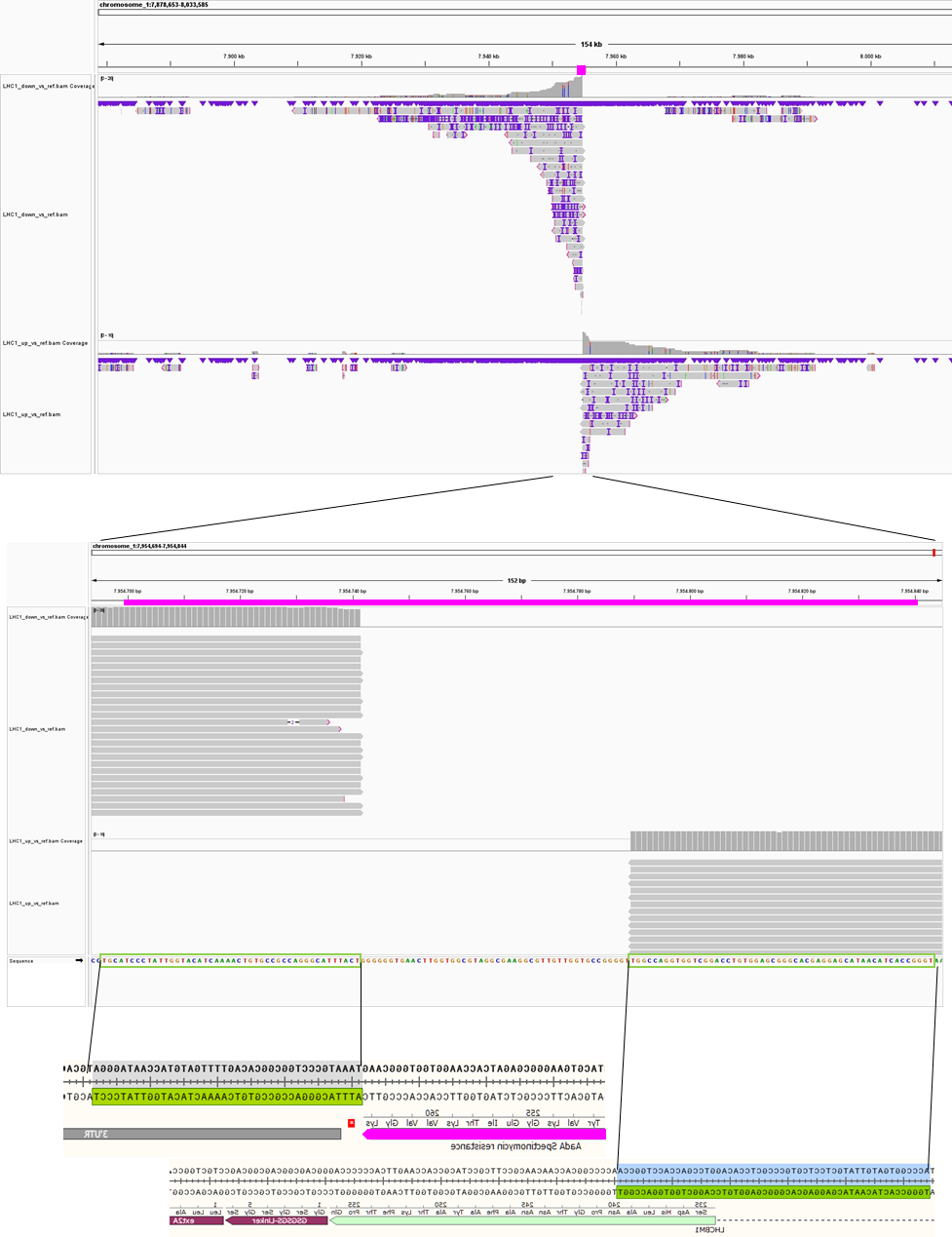
**

**Fig. S12 Nanopore sequencing results.** Whole genome nanopore sequencing was performed for LHCBM1 targeted expression strains expressing the *Cn*Vs and reads with oligos inside the insert were mapped on the *C. reinhardtii* genome (v5.6). Insertion was only found in the desired position (3’end of the LHCBM1 coding sequence) highlighted on the annotated LHCBM1 gene.

**
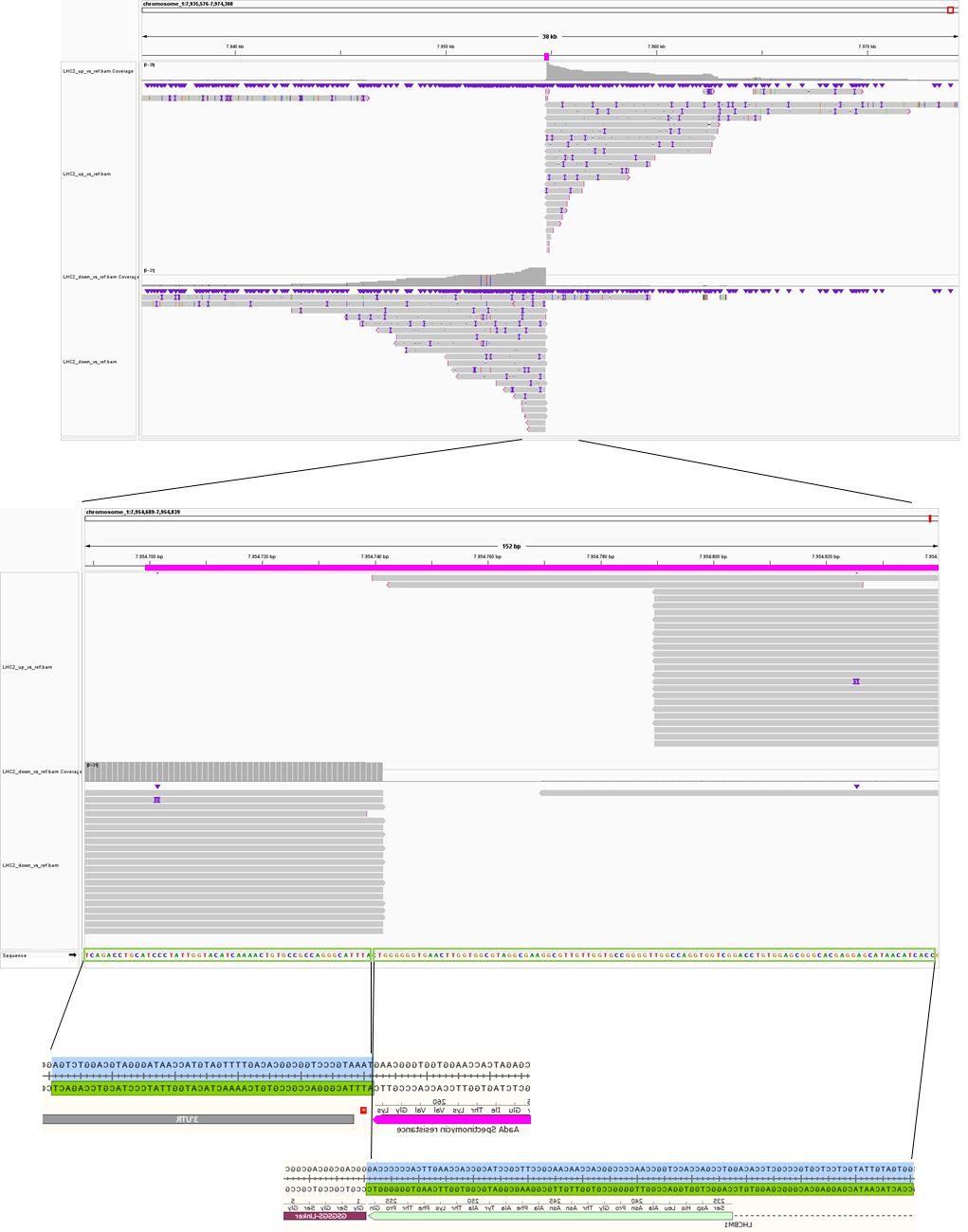
**

**Fig. S13 Nanopore sequencing results.** Whole genome nanopore sequencing was performed for LHCBM1 targeted expression strains expressing the *Cn*Vs and reads with oligos inside the insert were mapped on the *C. reinhardtii* genome (v5.6). Insertion was only found in the desired position (3’end of the LHCBM1 coding sequence) highlighted on the annotated LHCBM1 gene.

**
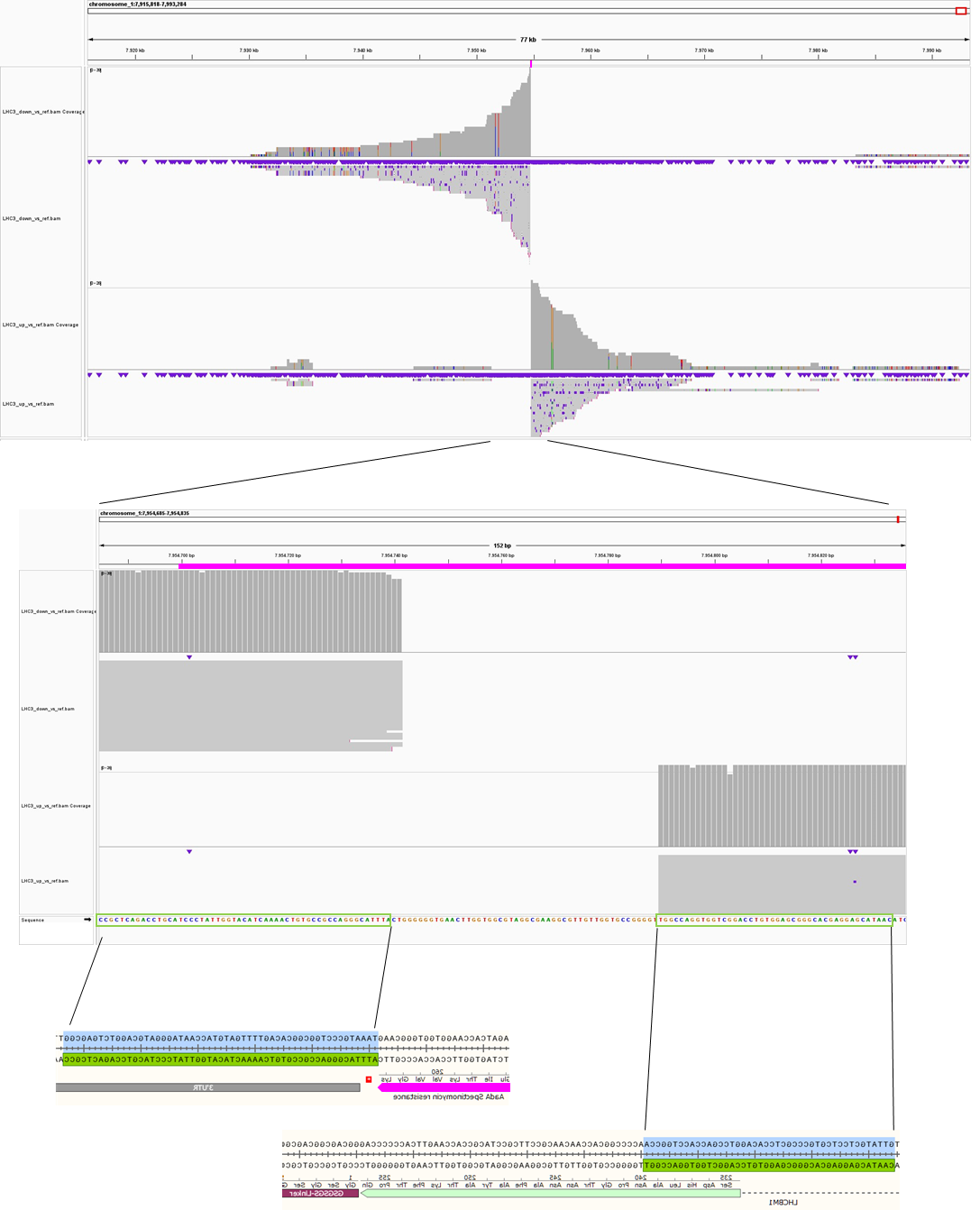
**

**Notes S1** **Expression vectors for nuclear transformation of *Chlamydomonas reinhardtii***

>Expression vectors for nuclear transformation of Chlamydomonas reinhardtii

>Traditional Expression vectors for nuclear transformation of Chlamydomonas reinhardtii

>Traditional expression of mVenus

LOCUS Exported 8364 bp ds-DNA circular SYN 02-AUG-2024

DEFINITION synthetic circular DNA

ACCESSION .

VERSION .

KEYWORDS .

SOURCE synthetic DNA construct

ORGANISM recombinant plasmid

REFERENCE 1 (bases 1 to 8364)

AUTHORS Thomas Baier

TITLE Direct Submission

JOURNAL Exported Friday, Aug 2, 2024 from SnapGene Viewer 4.3.11

https://www.snapgene.com

FEATURES Location/Qualifiers

source 1..8364

/organism="recombinant plasmid"

/mol_type="other DNA"

misc_feature 31..37

/label=Insertion Event

misc_feature 54..57

/label=fusion site

promoter 58..324

/label=HSP70Ap promoter

/label=HSP70Ap

promoter 331..522

/label=P-bTUB2

5'UTR join(523..598,744..798)

/label=5UTR bTUB2

intron 599..743

/label=RBCS2i

/label=RBCS2i(1)

misc_feature 799..802

/label=fusion site

CDS 803..820

/label=GSGSGS-Linker

gene join(821..1019,1165..1481,1627..1824)

/label=mVenus

intron 1020..1164

/label=rbcS2 intron 1

intron 1482..1626

/label=rbcS2 intron 1

CDS 1825..1842

/codon_start=1

/label=GSGSGS-Linker

/translation="GSGSGS"

misc_feature 1843..1846

/label=fusion site

CDS 1849..1866

/codon_start=1

/label=GSGSGS-Linker

/translation="GSGSGS"

CDS join(1867..2124,2270..2623,2769..2942)

/codon_start=1

/label=AadA Spectinomycin resistance

/translation="REAVIAEVSTQLSEVVGVIERHLEPTLLAVHLYGSAVDGGLKPHS

DIDLLVTVTVRLDETTRRALINDLLETSASPGESEILRAVEVTIVVHDDIIPWRYPAKR

ELQFGEWQRNDILAGIFEPATIDIDLAILLTKAREHSVALVGPAAEELFDPVPEQDLFE

ALNETLTLWNSPPDWAGDERNVVLTLSRIWYSAVTGKIAPKDVAADWAMERLPAQYQPV

ILEARQAYLGQEEDRLASRADQLEEFVHYVKGEITKVVGK"

intron 2125..2269

/label=rbcS2 intron 1

intron 2624..2768

/label=rbcS2 intron 1

CDS 2943..2960

/codon_start=1

/label=GSGSGS-Linker

/translation="GSGSGS"

misc_feature 2963..2966

/label=fusion site

misc_feature join(2967..2985,3315..3316)

/label=GSGS-Linker

intron 2986..3314

/label=rbcS2 intron 2

CDS 3317..3340

/codon_start=1

/product="peptide that binds Strep-Tactin(R), an engineered

form of streptavidin"

/label=Strep-Tag II

/translation="WSHPQFEK"

misc_feature 3344..3347

/label=fusion site

3'UTR 3348..4070

/label=FDX1 3'UTR

/note="FDX1 3'UTR with mutated SapI site"

misc_feature 4075..4098

/label=link1eb

/note="/vntifkey=21"

misc_feature 4139..4163

/label=RB T-DNA repeat

/note="right border repeat from nopaline C58 T-DNA"

rep_origin 4250..4961

/label=oriV

/note="incP origin of replication"

promoter 4982..5086

/gene="bla"

/label=AmpR promoter

CDS 5087..5902

/codon_start=1

/gene="aph(3')-Ia"

/product="aminoglycoside phosphotransferase"

/label=KanR

/note="confers resistance to kanamycin in bacteria or G418

(Geneticin(R)) in eukaryotes"

/translation="MSHIQRETSCSRPRLNSNMDADLYGYKWARDNVGQSGATIYRLYG

KPDAPELFLKHGKGSVANDVTDEMVRLNWLTEFMPLPTIKHFIRTPDDAWLLTTAIPGK

TAFQVLEEYPDSGENIVDALAVFLRRLHSIPVCNCPFNSDRVFRLAQAQSRMNNGLVDA

SDFDDERNGWPVEQVWKEMHKLLPFSPDSVVTHGDFSLDNLIFDEGKLIGCIDVGRVGI

ADRYQDLAILWNCLGEFSPSLQKRLFQKYGIDNPDMNKLQFHLMLDEFF"

rep_origin 6073..6661

/direction=RIGHT

/label=ori

/note="high-copy-number ColE1/pMB1/pBR322/pUC origin of

replication"

CDS 6997..8145

/codon_start=1

/product="trans-acting replication protein that binds to

and activates oriV"

/label=trfA

/translation="MNRTFDRKAYRQELIDAGFSAEDAETIASRTVMRAPRETFQSVGS

MVQQATAKIERDSVQLAPPALPAPSAAVERSRRLEQEAAGLAKSMTIDTRGTMTTKKRK

TAGEDLAKQVSEAKQAALLKHTKQQIKEMQLSLFDIAPWPDTMRAMPNDTARSALFTTR

NKKIPREALQNKVIFHVNKDVKITYTGVELRADDDELVWQQVLEYAKRTPIGEPITFTF

YELCQDLGWSINGRYYTKAEECLSRLQATAMGFTSDRVGHLESVSLLHRFRVLDRGKKT

SRCQVLIDEEIVVLFAGDHYTKFIWEKYRKLSPTARRMFDYFSSHREPYPLKLETFRLM

CGSDSTRVKKWREQVGEACEELRGSGLVEHAWVNDDLVHCKR"

misc_feature 8291..8315

/label=LB T-DNA repeat

/note="left border repeat from nopaline C58 T-DNA"

ORIGIN

1 actggggttg aaaatattcg atcgattgcc tgaggcctgc cgaattcgga tccggaggct

61 gaggcttgac atgattggtg cgtatgtttg tatgaagcta caggactgat ttggcgggct

121 atgagggcgg gggaagctct ggaagggccg cgatggggcg cgcggcgtcc agaaggcgcc

181 atacggcccg ctggcggcac ccatccggta taaaagcccg cgaccccgaa cggtgacctc

241 cactttcagc gacaaacgag cacttataca tacgcgacta ttctgccgct atacataacc

301 actcagctag cttaagatcc catcaccggt ctggcacttt cttgcgctat gacacttcca

361 gcaaaaggta gggcgggctg cgagacggct tcccggcgct gcatgcaaca ccgatgatac

421 ttatgcttcg accccccgaa gctccttcgg ggctgcatgg gcgctccgat gccgctccag

481 ggcgagcgct gtttaaatag ccaggccccc gactgcaaag acattatagc gagctaccaa

541 agccatactt caaacaccta gatcactacc acttctacac aggccactcg agcttgtggt

601 gagtcgacga gcaagcccgg cggatcaggc agcgtgcttg cagatttgac ttgcaacgcc

661 cgcattgtgt cgacgaaggc ttttggctcc tctgtcgctg tctcaagcag catctaaccc

721 tgcgtcgccg tttccatttg cagatcgcac tccgctaagg gggcgcctct tcctcttcgt

781 ttcagtcaca acccgcaaaa tgggcagcgg cagcggcagc gtgagcaagg gcgaggagct

841 gttcaccggc gtggtgccca tcctggtgga gctggacggc gacgtgaacg gccacaagtt

901 cagcgtgagc ggcgagggcg agggcgacgc cacctacggc aagctgaccc tgaagctgat

961 ctgcaccacc ggcaagctgc ccgtgccctg gcccaccctg gtgaccaccc tgggctacgg

1021 tgagtcgacg agcaagcccg gcggatcagg cagcgtgctt gcagatttga cttgcaacgc

1081 ccgcattgtg tcgacgaagg cttttggctc ctctgtcgct gtctcaagca gcatctaacc

1141 ctgcgtcgcc gtttccattt gcaggcctgc agtgcttcgc ccgctacccc gaccacatga

1201 agcagcacga cttcttcaag agcgccatgc ccgagggcta cgtgcaggag cgcaccatct

1261 tcttcaagga cgacggtaac tacaagaccc gcgccgaggt gaagttcgag ggcgacaccc

1321 tggtgaaccg catcgagctg aagggcatcg acttcaagga ggacggcaac atcctgggcc

1381 acaagctgga gtacaactac aacagccaca acgtgtacat caccgccgac aagcagaaga

1441 acggcatcaa ggccaacttc aagatccgcc acaacatcga ggtgagtcga cgagcaagcc

1501 cggcggatca ggcagcgtgc ttgcagattt gacttgcaac gcccgcattg tgtcgacgaa

1561 ggcttttggc tcctctgtcg ctgtctcaag cagcatctaa ccctgcgtcg ccgtttccat

1621 ttgcaggacg gcggcgtgca gctggccgac cactaccagc agaacacccc catcggcgac

1681 ggccccgtgc tgctgcccga caaccactac ctgagctacc agagcaagct gagcaaggac

1741 cccaacgaga agcgcgacca catggtgctg ctggagttcg tgaccgccgc cggcatcacc

1801 ctgggcatgg acgagctgta caagggcagc ggcagcggca gcaggtcggg cagcggcagc

1861 ggcagccgcg aggccgtgat cgccgaggtg agcacccagc tgagcgaggt ggtgggcgtg

1921 atcgagcgcc acctggagcc caccctgctg gccgtgcacc tgtacggcag cgccgtggac

1981 ggcggcctga agccccacag cgacatcgac ctgctggtga ccgtgaccgt gcgcctggac

2041 gagacgaccc gccgcgccct gatcaacgac ctgctggaga cgagcgccag ccccggcgag

2101 agcgagatcc tgcgcgccgt ggaggtgagt cgacgagcaa gcccggcgga tcaggcagcg

2161 tgcttgcaga tttgacttgc aacgcccgca ttgtgtcgac gaaggctttt ggctcctctg

2221 tcgctgtctc aagcagcatc taaccctgcg tcgccgtttc catttgcagg tgaccatcgt

2281 ggtgcacgac gacatcatcc cctggcgcta ccccgccaag cgcgagctgc agttcggcga

2341 gtggcagcgc aacgacatcc tggccggcat cttcgagccc gccaccatcg acatcgacct

2401 ggccatcctg ctgaccaagg cccgcgagca cagcgtggcc ctggtgggcc ccgccgccga

2461 ggagctgttc gaccccgtgc ccgagcagga cctgttcgag gccctgaacg agacgctgac

2521 cctgtggaac agcccccccg actgggccgg cgacgagcgc aacgtggtgc tgaccctgag

2581 ccgcatctgg tacagcgccg tgaccggcaa gatcgccccc aaggtgagtc gacgagcaag

2641 cccggcggat caggcagcgt gcttgcagat ttgacttgca acgcccgcat tgtgtcgacg

2701 aaggcttttg gctcctctgt cgctgtctca agcagcatct aaccctgcgt cgccgtttcc

2761 atttgcagga cgtggccgcc gactgggcca tggagcgcct gcccgcccag taccagcccg

2821 tgatcctgga ggcccgccag gcctacctgg gccaggagga ggaccgcctg gccagccgcg

2881 ccgaccagct ggaggagttc gtgcactacg tgaagggcga gatcaccaag gtggtgggca

2941 agggcagcgg cagcggcagc gcttcgggca gcggcagcgg ctcaggtgag cttgcggggt

3001 tgcgagcaac actccagcaa cgaacagtgc ccaagtcagg aatctgcagt cagcctgggc

3061 tttcggcggc tttttcttgg gcaaacagct tgcactcatg ccagcgcggc ttgtccagcc

3121 tcacttgagc tttccagctg ctaccagccg ggctatacga cagcgacaga gccatagcgt

3181 ggaatcactt atttgggttg ccgaagtagc ggtcggagcg tgagttcttg gtcaagccgc

3241 cccttatccg gttcctgtcc gtgtctttgt ccctcgttca cccttcgcgg cacccttcat

3301 ccccttgctt gcaggttgga gccacccgca gttcgagaag taagcttgcg ctttcgccat

3361 ctgcgggggt cgtaggctag aactggggtt ggggatcggg ctgcttgcat agccaagcaa

3421 ttttccatct ggccagcaat ggcctagcac tatgagcggt tcaagtgtct cttgtgtgtt

3481 gtgtcgcatt gcatggccgt ggtgacctgc aattttctgt aaccggacat gcagaagctt

3541 cggttcgcgt cctttcctcg cttgctacgg gatcgggagt cggcagggct agaagtcttg

3601 ggtaacacgc gcaattcagc aatacaggcc agccagggca gcgaaggggg acttcagcaa

3661 ggacctctcg ggaatagtgg atgagctagg aggggtacag cagtagagat cgaggggtcc

3721 agctcacagt tctattacgt cgttcgtggg gacgaactgg gtcgaggcgc tacggactcg

3781 aaatgacggc agagggtggc aaggaagggg agcaccactg tgagcagttg cagcggcaca

3841 tacactacgt ctcttggcct taagcacagc cagcacactt gtacggggca acagtagccc

3901 cgaagcagcc tgatgcagtc acaccgtgcc gggccagtgt taacaaggaa gggcaggcac

3961 cagggcgagg gcaggcgcgg caaaactcgc cggttcctga cacggtgaca cgcaggtata

4021 cggtgacagc tcagctagtg ataccagctg ctccgcgttc tgaggagagg cgctgcaaga

4081 ggatgcacat gtgaccgagg gatacgatcg aatattatcc gtttaaacta tcagtgtttg

4141 acaggatata ttggcgggta aacctaagag aaaagagcgt ttattagaat aatcggatat

4201 ttaaaagggc gtgaaaaggt ttatccgttc gtccatttgt atgtgccagc cgcctttgcg

4261 acgctcaccg ggctggttgc cctcgccgct gggctggcgg ccgtctatgg ccctgcaaac

4321 gcgccagaaa cgccgtcgaa gccgtgtgcg agacaccgcg gccgccggcg ttgtggatac

4381 ctcgcggaaa acttggccct cactgacaga tgaggggcgg acgttgacac ttgaggggcc

4441 gactcacccg gcgcggcgtt gacagatgag gggcaggctc gatttcggcc ggcgacgtgg

4501 agctggccag cctcgcaaat cggcgaaaac gcctgatttt acgcgagttt cccacagatg

4561 atgtggacaa gcctggggat aagtgccctg cggtattgac acttgagggg cgcgactact

4621 gacagatgag gggcgcgatc cttgacactt gaggggcaga gtgctgacag atgaggggcg

4681 cacctattga catttgaggg gctgtccaca ggcagaaaat ccagcatttg caagggtttc

4741 cgcccgtttt tcggccaccg ctaacctgtc ttttaacctg cttttaaacc aatatttata

4801 aaccttgttt ttaaccaggg ctgcgccctg tgcgcgtgac cgcgcacgcc gaaggggggt

4861 gccccccctt ctcgaaccct cccggcccgc taacgcgggc ctcccatccc cccaggggct

4921 gcgcccctcg gccgcgaacg gcctcacccc aaaaatggca gcgctggcca attcccgagt

4981 gcgcggaacc cctatttgtt tatttttcta aatacattca aatatgtatc cgctcatgag

5041 acaataaccc tgataaatgc ttcaataata ttgaaaaagg aagagtatga gccatattca

5101 acgggaaacg tcttgctcta ggccgcgatt aaattccaac atggatgctg atttatatgg

5161 gtataaatgg gctcgcgata atgtcgggca atcaggtgcg acaatctatc gattgtatgg

5221 gaagcccgat gcgccagagt tgtttctgaa acatggcaaa ggtagcgttg ccaatgatgt

5281 tacagatgag atggtcagac taaactggct gacggaattt atgcctcttc cgaccatcaa

5341 gcattttatc cgtactcctg atgatgcatg gttactcacc actgcgatcc ccgggaaaac

5401 agcattccag gtattagaag aatatcctga ttcaggtgaa aatattgttg atgcgctggc

5461 agtgttcctg cgccggttgc attcgattcc tgtttgtaat tgtcctttta acagcgatcg

5521 cgtatttcgt ctcgctcagg cgcaatcacg aatgaataac ggtttggttg atgcgagtga

5581 ttttgatgac gagcgtaatg gctggcctgt tgaacaagtc tggaaagaaa tgcataaact

5641 tttgccattc tcaccggatt cagtcgtcac tcatggtgat ttctcacttg ataaccttat

5701 ttttgacgag gggaaattaa taggttgtat tgatgttgga cgagtcggaa tcgcagaccg

5761 ataccaggat cttgccatcc tatggaactg cctcggtgag ttttctcctt cattacagaa

5821 acggcttttt caaaaatatg gtattgataa tcctgatatg aataaattgc agtttcattt

5881 gatgctcgat gagtttttct aactgtcaga ccaagtttac tcatatatac tttagattga

5941 tttaaaactt catttttaat ttaaaaggat ctaggtgaag atcctttttg ataatctcat

6001 gaccaaaatc ccttaacgtg agttttcgtt ccactgagcg tcagaccccg tagaaaagat

6061 caaaggatct tcttgagatc ctttttttct gcgcgtaatc tgctgcttgc aaacaaaaaa

6121 accaccgcta ccagcggtgg tttgtttgcc ggatcaagag ctaccaactc tttttccgaa

6181 ggtaactggc ttcagcagag cgcagatacc aaatactgtc cttctagtgt agccgtagtt

6241 aggccaccac ttcaagaact ctgtagcacc gcctacatac ctcgctctgc taatcctgtt

6301 accagtggct gctgccagtg gcgataagtc gtgtcttacc gggttggact caagacgata

6361 gttaccggat aaggcgcagc ggtcgggctg aacggggggt tcgtgcacac agcccagctt

6421 ggagcgaacg acctacaccg aactgagata cctacagcgt gagctatgag aaagcgccac

6481 gcttcccgaa gggagaaagg cggacaggta tccggtaagc ggcagggtcg gaacaggaga

6541 gcgcacgagg gagcttccag ggggaaacgc ctggtatctt tatagtcctg tcgggtttcg

6601 ccacctctga cttgagcgtc gatttttgtg atgctcgtca ggggggcgga gcctatggaa

6661 aaacgccagc aacgcggcct ttttacggtt cctggcagat cctagatgtg gcgcaacgat

6721 gccggcgaca agcaggagcg caccgacttc ttccgcatca agtgttttgg ctctcaggcc

6781 gaggcccacg gcaagtattt gggcaagggg tcgctggtat tcgtgcaggg caagattcgg

6841 aataccaagt acgagaagga cggccagacg gtctacggga ccgacttcat tgccgataag

6901 gtggattatc tggacaccaa ggcaccaggc gggtcaaatc aggaataagg gcacattgcc

6961 ccggcgtgag tcggggcaat cccgcaagga gggtgaatga atcggacgtt tgaccggaag

7021 gcatacaggc aagaactgat cgacgcgggg ttttccgccg aggatgccga aaccatcgca

7081 agccgcaccg tcatgcgtgc gccccgcgaa accttccagt ccgtcggctc gatggtccag

7141 caagctacgg ccaagatcga gcgcgacagc gtgcaactgg ctccccctgc cctgcccgcg

7201 ccatcggccg ccgtggagcg ttcgcgtcgt cttgaacagg aggcggcagg tttggcgaag

7261 tcgatgacca tcgacacgcg aggaactatg acgaccaaga agcgaaaaac cgccggcgag

7321 gacctggcaa aacaggtcag cgaggccaag caggccgcgt tgctgaaaca cacgaagcag

7381 cagatcaagg aaatgcagct ttccttgttc gatattgcgc cgtggccgga cacgatgcga

7441 gcgatgccaa acgacacggc ccgctctgcc ctgttcacca cgcgcaacaa gaaaatcccg

7501 cgcgaggcgc tgcaaaacaa ggtcattttc cacgtcaaca aggacgtgaa gatcacctac

7561 accggcgtcg agctgcgggc cgacgatgac gaactggtgt ggcagcaggt gttggagtac

7621 gcgaagcgca cccctatcgg cgagccgatc accttcacgt tctacgagct ttgccaggac

7681 ctgggctggt cgatcaatgg ccggtattac acgaaggccg aggaatgcct gtcgcgccta

7741 caggcgacgg cgatgggctt cacgtccgac cgcgttgggc acctggaatc ggtgtcgctg

7801 ctgcaccgct tccgcgtcct ggaccgtggc aagaaaacgt cccgttgcca ggtcctgatc

7861 gacgaggaaa tcgtcgtgct gtttgctggc gaccactaca cgaaattcat atgggagaag

7921 taccgcaagc tgtcgccgac ggcccgacgg atgttcgact atttcagctc gcaccgggag

7981 ccgtacccgc tcaagctgga aaccttccgc ctcatgtgcg gatcggattc cacccgcgtg

8041 aagaagtggc gcgagcaggt cggcgaagcc tgcgaagagt tgcgaggcag cggcctggtg

8101 gaacacgcct gggtcaatga tgacctggtg cattgcaaac gctagggcct tgtggggtca

8161 gttccggctg ggggttcagc agcccctgct cggatctgtt ggaccggaca gtagtcatgg

8221 ttgatgggct gcctgtatcg agtggtgatt ttgtgccgag ctgccggtcg gggagctgtt

8281 ggctggctgg tggcaggata tattgtggtg taaacaaatt gacgcttaga caacttaata

8341 acacattgcg gacgttttta atgt

//

>Traditional expression of CnVs

LOCUS Exported 11253 bp ds-DNA circular SYN 02-AUG-2024

DEFINITION synthetic circular DNA

ACCESSION .

VERSION .

KEYWORDS .

SOURCE synthetic DNA construct

ORGANISM recombinant plasmid

REFERENCE 1 (bases 1 to 11253)

AUTHORS Thomas Baier

TITLE Direct Submission

JOURNAL Exported Friday, Aug 2, 2024 from SnapGene Viewer 4.3.11

https://www.snapgene.com

FEATURES Location/Qualifiers

source 1..11253

/organism="recombinant plasmid"

/mol_type="other DNA"

promoter 28..294

/label=P-HSP70A

promoter 306..498

/label=P-RBCS2

5'UTR 499..521

/label=5UTR CrRBCS2

CDS 523..1323

/codon_start=1

/label=AphVIII

/translation="MDDALRALRGRYPGCEWVVVEDGASGAGVYRLRGGGRELFVKVAA

LGAGVGLLGEAERLVWLAEVGIPVPRVVEGGGDERVAWLVTEAVPGRPASARWPREQRL

DVAVALAGLARSLHALDWERCPFDRSLAVTVPQAARAVAEGSVDLEDLDEERKGWSGER

LLAELERTRPADEDLAVCHGDLCPDNVLLDPRTCEVTGLIDVGRVGRADRHSDLALVLR

ELAHEEDPWFGPECSAAFLREYGRGWDGAVSEEKLAFYRLLDEFF"

terminator 1331..1564

/label=T-RBCS2

misc_feature 1577..1580

/label=fusion site

promoter 1581..1847

/label=HSP70Ap promoter

/label=HSP70Ap

promoter 1854..2045

/label=P-bTUB2

misc_feature 1940..1945

/label=inserted ATANTT motif

5'UTR join(2046..2121,2267..2321)

/label=5UTR bTUB2

misc_feature 2068..2073

/label=rebuild ATANTT from ATATT

intron 2122..2266

/label=RBCS2i

/label=RBCS2i(1)

misc_feature 2322..2325

/label=fusion site

CDS join(2326..2423,2569..2956,3102..3503,3649..4060,4206..4592,

4738..4814)

/codon_start=1

/product="AFN21429.1 terpene synthase Valencene synthase

[Callitropsis nootkatensis] Beekwilder 2014"

/label=AFN21429.1 terpene synthase Valencene synthase

/label=AFN21429.1 terpene synthase Valencene synthase

[Callitropsis nootkatensis] Beekwilder 2014__CDS

/translation="AEMFNGNSSNDGSSCMPVKDALRRTGNHHPNLWTDDFIQSLNSPY

SDSSYHKHREILIDEIRDMFSNGEGDEFGVLENIWFVDVVQRLGIDRHFQEEIKTALDY

IYKFWNHDSIFGDLNMVALGFRILRLNRYVASSDVFKKFKGEEGQFSGFESSDQDAKLE

MMLNLYKASELDFPDEDILKEARAFASMYLKHVIKEYGDIQESKNPLLMEIEYTFKYPW

RCRLPRLEAWNFIHIMRQQDCNISLANNLYKIPKIYMKKILELAILDFNILQSQHQHEM

KLISTWWKNSSAIQLDFFRHRHIESYFWWASPLFEPEFSTCRINCTKLSTKMFLLDDIY

DTYGTVEELKPFTTTLTRWDVSTVDNHPDYMKIAFNFSYEIYKEIASEAERKHGPFVYK

YLQSCWKSYIEAYMQEAEWIASNHIPGFDEYLMNGVKSSGMRILMIHALILMDTPLSDE

ILEQLDIPSSKSQALLSLITRLVDDVKDFEDEQAHGEMASSIECYMKDNHGSTREDALN

YLKIRIESCVQELNKELLEPSNMHGSFRNLYLNVGMRVIFFMLNDGDLFTHSNRKEIQD

AITKFFVEPIIP"

intron 2424..2568

/label=intron

intron 2957..3101

/label=intron

intron 3504..3648

/label=intron

intron 4061..4205

/label=intron

intron 4593..4737

/label=intron

CDS 4815..4832

/codon_start=1

/label=GSGSGS-Linker

/translation="GSGSGS"

misc_feature 4833..4836

/label=fusion site

CDS 4839..4856

/label=GSGSGS-Linker

gene join(4857..5055,5201..5517,5663..5860)

/label=mVenus

misc_feature 4969..4991

/label=sgRNA-target site

misc_feature 5028..5050

/label=sgRNA-target site

intron 5056..5200

/label=rbcS2 intron 1

misc_feature 5202..5224

/label=sgRNA-target-exclusive

intron 5518..5662

/label=rbcS2 intron 1

CDS 5861..5878

/codon_start=1

/label=GSGSGS-Linker

/translation="GSGSGS"

misc_feature 5881..5884

/label=fusion site

misc_feature join(5885..5903,6233..6234)

/label=GSGS-Linker

intron 5904..6232

/label=rbcS2 intron 2

CDS 6235..6258

/codon_start=1

/product="peptide that binds Strep-Tactin(R), an engineered

form of streptavidin"

/label=Strep-Tag II

/translation="WSHPQFEK"

misc_feature 6262..6265

/label=fusion site

3'UTR 6266..6988

/label=FDX1 3'UTR

misc_feature 7027..7051

/label=RB T-DNA repeat

/note="right border repeat from nopaline C58 T-DNA"

rep_origin 7139..7849

/label=oriV

/note="incP origin of replication"

promoter 7870..7974

/gene="bla"

/label=AmpR promoter

CDS 7975..8790

/codon_start=1

/gene="aph(3')-Ia"

/product="aminoglycoside phosphotransferase"

/label=KanR

/note="confers resistance to kanamycin in bacteria or G418

(Geneticin(R)) in eukaryotes"

/translation="MSHIQRETSCSRPRLNSNMDADLYGYKWARDNVGQSGATIYRLYG

KPDAPELFLKHGKGSVANDVTDEMVRLNWLTEFMPLPTIKHFIRTPDDAWLLTTAIPGK

TAFQVLEEYPDSGENIVDALAVFLRRLHSIPVCNCPFNSDRVFRLAQAQSRMNNGLVDA

SDFDDERNGWPVEQVWKEMHKLLPFSPDSVVTHGDFSLDNLIFDEGKLIGCIDVGRVGI

ADRYQDLAILWNCLGEFSPSLQKRLFQKYGIDNPDMNKLQFHLMLDEFF"

rep_origin 8961..9549

/direction=RIGHT

/label=ori

/note="high-copy-number ColE1/pMB1/pBR322/pUC origin of

replication"

CDS 9885..11033

/codon_start=1

/product="trans-acting replication protein that binds to

and activates oriV"

/label=trfA

/translation="MNRTFDRKAYRQELIDAGFSAEDAETIASRTVMRAPRETFQSVGS

MVQQATAKIERDSVQLAPPALPAPSAAVERSRRLEQEAAGLAKSMTIDTRGTMTTKKRK

TAGEDLAKQVSEAKQAALLKHTKQQIKEMQLSLFDIAPWPDTMRAMPNDTARSALFTTR

NKKIPREALQNKVIFHVNKDVKITYTGVELRADDDELVWQQVLEYAKRTPIGEPITFTF

YELCQDLGWSINGRYYTKAEECLSRLQATAMGFTSDRVGHLESVSLLHRFRVLDRGKKT

SRCQVLIDEEIVVLFAGDHYTKFIWEKYRKLSPTARRMFDYFSSHREPYPLKLETFRLM

CGSDSTRVKKWREQVGEACEELRGSGLVEHAWVNDDLVHCKR"

misc_feature 11179..11203

/label=LB T-DNA repeat

/note="left border repeat from nopaline C58 T-DNA"

ORIGIN

1 ctggggttga aaatattcga tcgtgccgct gaggcttgac atgattggtg cgtatgtttg

61 tatgaagcta caggactgat ttggcgggct atgagggcgg gggaagctct ggaagggccg

121 cgatggggcg cgcggcgtcc agaaggcgcc atacggcccg ctggcggcac ccatccggta

181 taaaagcccg cgaccccgaa cggtgacctc cactttcagc gacaaacgag cacttataca

241 tacgcgacta ttctgccgct atacataacc actcagctag cttaagatcc catcaagctt

301 gcatgccggg cgcgccagaa ggagcgcagc caaaccagga tgatgtttga tggggtattt

361 gagcacttgc aacccttatc cggaagcccc ctggcccaca aaggctaggc gccaatgcaa

421 gcagttcgca tgcagcccct ggagcggtgc cctcctgata aaccggccag ggggcctatg

481 ttctttactt ttttacaaga gaagtcactc aacatcttaa aaatggacga tgcgttgcgt

541 gcactgcggg gtcggtatcc cggttgtgag tgggttgttg tggaggatgg ggcctcgggg

601 gctggtgttt atcggcttcg gggtggtggg cgggagttgt ttgtcaaggt ggcagctctg

661 ggggccgggg tgggcttgtt gggtgaggct gagcggctgg tgtggttggc ggaggtgggg

721 attcccgtac ctcgtgttgt ggagggtggt ggggacgaga gggtcgcctg gttggtcacc

781 gaagcggttc cggggcgtcc ggccagtgcg cggtggccgc gggagcagcg gctggacgtg

841 gcggtggcgc tcgcggggct cgctcgttcg ctgcacgcgc tggactggga gcggtgtccg

901 ttcgatcgca gtctcgcggt gacggtgccg caggcggccc gtgctgtcgc tgaagggagc

961 gtcgacttgg aggatctgga cgaggagcgg aaggggtggt cgggggagcg gcttctcgcc

1021 gagctggagc ggactcggcc tgcggacgag gatctggcgg tttgccacgg tgacctgtgc

1081 ccggacaacg tgctgctcga ccctcgtacc tgcgaggtga ccgggctgat cgacgtgggg

1141 cgggtcggcc gtgcggaccg gcactccgat ctcgcgctgg tgctgcgcga gctggcccac

1201 gaggaggacc cgtggttcgg gccggagtgt tccgcggcgt tcctgcggga gtacgggcgc

1261 gggtgggatg gggcggtatc ggaggaaaag ctggcgtttt accggctgtt ggacgagttc

1321 ttctgagctt ccgctccgtg taaatggagg cgctcgttga tctgagcctt gccccctgac

1381 gaacggcggt ggatggaaga tactgctctc aagtgctgaa gcggtagctt agctccccgt

1441 ttcgtgctga tcagtctttt tcaacacgta aaaagcggag gagttttgca attttgttgg

1501 ttgtaacgat cctccgttga ttttggcctc tttctccatg ggcgggctgg gcgtatttga

1561 agcggaattc aagcttggag gctgaggctt gacatgattg gtgcgtatgt ttgtatgaag

1621 ctacaggact gatttggcgg gctatgaggg cgggggaagc tctggaaggg ccgcgatggg

1681 gcgcgcggcg tccagaaggc gccatacggc ccgctggcgg cacccatccg gtataaaagc

1741 ccgcgacccc gaacggtgac ctccactttc agcgacaaac gagcacttat acatacgcga

1801 ctattctgcc gctatacata accactcagc tagcttaaga tcccatcacc ggtctggcac

1861 tttcttgcgc tatgacactt ccagcaaaag gtagggcggg ctgcgagacg gcttcccggc

1921 gctgcatgca acaccgatga tacttatgct tcgacccccc gaagctcctt cggggctgca

1981 tgggcgctcc gatgccgctc cagggcgagc gctgtttaaa tagccaggcc cccgactgca

2041 aagacattat agcgagctac caaagccata cttcaaacac ctagatcact accacttcta

2101 cacaggccac tcgagcttgt ggtgagtcga cgagcaagcc cggcggatca ggcagcgtgc

2161 ttgcagattt gacttgcaac gcccgcattg tgtcgacgaa ggcttttggc tcctctgtcg

2221 ctgtctcaag cagcatctaa ccctgcgtcg ccgtttccat ttgcagatcg cactccgcta

2281 agggggcgcc tcttcctctt cgtttcagtc acaacccgca aaatggccga gatgttcaac

2341 ggcaacagca gcaacgacgg cagcagctgc atgcccgtga aggacgccct gcgccgcacc

2401 ggcaaccacc accccaacct gtggtgagtc gacgagcaag cccggcggat caggcagcgt

2461 gcttgcagat ttgacttgca acgcccgcat tgtgtcgacg aaggcttttg gctcctctgt

2521 cgctgtctca agcagcatct aaccctgcgt cgccgtttcc atttgcagga ccgacgactt

2581 catccagagc ctgaacagcc cctacagcga cagcagctac cacaagcacc gcgagatcct

2641 gatcgacgag atccgcgaca tgttcagcaa cggcgagggc gacgagttcg gcgtgctgga

2701 gaacatctgg ttcgtggacg tggtgcagcg cctgggcatc gaccgccact tccaggagga

2761 gatcaagacc gccctggact acatctacaa gttctggaac cacgacagca tcttcggcga

2821 cctgaacatg gtggccctgg gcttccgcat cctgcgcctg aaccgctacg tggccagcag

2881 cgacgtgttc aagaagttca agggcgagga gggccagttc agcggcttcg agagcagcga

2941 ccaggacgcc aagctggtga gtcgacgagc aagcccggcg gatcaggcag cgtgcttgca

3001 gatttgactt gcaacgcccg cattgtgtcg acgaaggctt ttggctcctc tgtcgctgtc

3061 tcaagcagca tctaaccctg cgtcgccgtt tccatttgca ggagatgatg ctgaacctgt

3121 acaaggccag cgagctggac ttccccgacg aggacatcct gaaggaggcc cgcgccttcg

3181 ccagcatgta cctgaagcac gtgatcaagg agtacggcga catccaggag agcaagaacc

3241 ccctgctgat ggagatcgag tacaccttca agtacccctg gcgctgccgc ctgccccgcc

3301 tggaggcctg gaacttcatc cacatcatgc gccagcagga ctgcaacatc agcctggcca

3361 acaacctgta caagatcccc aagatttaca tgaagaagat cctggagctg gccatcctgg

3421 acttcaacat cctgcagagc cagcaccagc acgagatgaa gctgatcagc acctggtgga

3481 agaacagcag cgccatccag ctggtgagtc gacgagcaag cccggcggat caggcagcgt

3541 gcttgcagat ttgacttgca acgcccgcat tgtgtcgacg aaggcttttg gctcctctgt

3601 cgctgtctca agcagcatct aaccctgcgt cgccgtttcc atttgcagga cttcttccgc

3661 caccgccaca tcgagagcta cttctggtgg gccagccccc tgttcgagcc cgagttcagc

3721 acctgccgca tcaactgcac caagctgagc accaagatgt tcctgctgga cgacatctac

3781 gacacctacg gcaccgtgga ggagctgaag cccttcacca ccaccctgac ccgctgggac

3841 gtgagcaccg tggacaacca ccccgactac atgaagatcg ccttcaactt cagctacgag

3901 atttacaagg agatcgccag cgaggccgag cgcaagcacg gccccttcgt gtacaagtac

3961 ctgcagagct gctggaagag ctacatcgag gcctacatgc aggaggccga gtggatcgcc

4021 agcaaccaca tccccggctt cgacgagtac ctgatgaacg gtgagtcgac gagcaagccc

4081 ggcggatcag gcagcgtgct tgcagatttg acttgcaacg cccgcattgt gtcgacgaag

4141 gcttttggct cctctgtcgc tgtctcaagc agcatctaac cctgcgtcgc cgtttccatt

4201 tgcaggcgtg aagagcagcg gcatgcgcat cctgatgatc cacgccctga tcctgatgga

4261 cacccccctg agcgacgaga tcctggagca gctggacatc cccagcagca agagccaggc

4321 cctgctgagc ctgatcaccc gcctggtgga cgacgtgaag gacttcgagg acgagcaggc

4381 ccacggcgag atggccagca gcatcgagtg ctacatgaag gacaaccacg gcagcacccg

4441 cgaggacgcc ctgaactacc tgaagatccg catcgagagc tgcgtgcagg agctgaacaa

4501 ggagctgctg gagcccagca acatgcacgg cagcttccgc aacctgtacc tgaacgtggg

4561 catgcgcgtg atcttcttca tgctgaacga cggtgagtcg acgagcaagc ccggcggatc

4621 aggcagcgtg cttgcagatt tgacttgcaa cgcccgcatt gtgtcgacga aggcttttgg

4681 ctcctctgtc gctgtctcaa gcagcatcta accctgcgtc gccgtttcca tttgcaggcg

4741 acctgttcac ccacagcaac cgcaaggaga tccaggacgc catcaccaag ttcttcgtgg

4801 agcccatcat ccccggcagc ggcagcggca gcaggtcggg cagcggcagc ggcagcgtga

4861 gcaagggcga ggagctgttc accggcgtgg tgcccatcct ggtggagctg gacggcgacg

4921 tgaacggcca caagttcagc gtgagcggcg agggcgaggg cgacgccacc tacggcaagc

4981 tgaccctgaa gctgatctgc accaccggca agctgcccgt gccctggccc accctggtga

5041 ccaccctggg ctacggtgag tcgacgagca agcccggcgg atcaggcagc gtgcttgcag

5101 atttgacttg caacgcccgc attgtgtcga cgaaggcttt tggctcctct gtcgctgtct

5161 caagcagcat ctaaccctgc gtcgccgttt ccatttgcag gcctgcagtg cttcgcccgc

5221 taccccgacc acatgaagca gcacgacttc ttcaagagcg ccatgcccga gggctacgtg

5281 caggagcgca ccatcttctt caaggacgac ggtaactaca agacccgcgc cgaggtgaag

5341 ttcgagggcg acaccctggt gaaccgcatc gagctgaagg gcatcgactt caaggaggac

5401 ggcaacatcc tgggccacaa gctggagtac aactacaaca gccacaacgt gtacatcacc

5461 gccgacaagc agaagaacgg catcaaggcc aacttcaaga tccgccacaa catcgaggtg

5521 agtcgacgag caagcccggc ggatcaggca gcgtgcttgc agatttgact tgcaacgccc

5581 gcattgtgtc gacgaaggct tttggctcct ctgtcgctgt ctcaagcagc atctaaccct

5641 gcgtcgccgt ttccatttgc aggacggcgg cgtgcagctg gccgaccact accagcagaa

5701 cacccccatc ggcgacggcc ccgtgctgct gcccgacaac cactacctga gctaccagag

5761 caagctgagc aaggacccca acgagaagcg cgaccacatg gtgctgctgg agttcgtgac

5821 cgccgccggc atcaccctgg gcatggacga gctgtacaag ggcagcggca gcggcagcgc

5881 ttcgggcagc ggcagcggct caggtgagct tgcggggttg cgagcaacac tccagcaacg

5941 aacagtgccc aagtcaggaa tctgcagtca gcctgggctt tcggcggctt tttcttgggc

6001 aaacagcttg cactcatgcc agcgcggctt gtccagcctc acttgagctt tccagctgct

6061 accagccggg ctatacgaca gcgacagagc catagcgtgg aatcacttat ttgggttgcc

6121 gaagtagcgg tcggagcgtg agttcttggt caagccgccc cttatccggt tcctgtccgt

6181 gtctttgtcc ctcgttcacc cttcgcggca cccttcatcc ccttgcttgc aggttggagc

6241 cacccgcagt tcgagaagta agcttgcgct ttcgccatct gcgggggtcg taggctagaa

6301 ctggggttgg ggatcgggct gcttgcatag ccaagcaatt ttccatctgg ccagcaatgg

6361 cctagcacta tgagcggttc aagtgtctct tgtgtgttgt gtcgcattgc atggccgtgg

6421 tgacctgcaa ttttctgtaa ccggacatgc agaagcttcg gttcgcgtcc tttcctcgct

6481 tgctacggga tcgggagtcg gcagggctag aagtcttggg taacacgcgc aattcagcaa

6541 tacaggccag ccagggcagc gaagggggac ttcagcaagg acctctcggg aatagtggaa

6601 gagctaggag gggtacagca gtagagatcg aggggtccag ctcacagttc tattacgtcg

6661 ttcgtgggga cgaactgggt cgaggcgcta cggactcgaa atgacggcag agggtggcaa

6721 ggaaggggag caccactgtg agcagttgca gcggcacata cactacgtct cttggcctta

6781 agcacagcca gcacacttgt acggggcaac agtagccccg aagcagcctg atgcagtcac

6841 accgtgccgg gccagtgtta acaaggaagg gcaggcacca gggcgagggc aggcgcggca

6901 aaactcgccg gttcctgaca cggtgacacg caggtatacg gtgacagctc agctagtgat

6961 accagctgct ccgcgttctg aggagaggcg ctcgatcgaa tattatccgt ttaaactatc

7021 agtgtttgac aggatatatt ggcgggtaaa cctaagagaa aagagcgttt attagaataa

7081 tcggatattt aaaagggcgt gaaaaggttt atccgttcgt ccatttgtat gtgccagccg

7141 cctttgcgac gctcaccggg ctggttgccc tcgccgctgg gctggcggcc gtctatggcc

7201 ctgcaaacgc gccagaaacg ccgtcgaagc cgtgtgcgag acaccgcggc cgccggcgtt

7261 gtggatacct cgcggaaaac ttggccctca ctgacagatg aggggcggac gttgacactt

7321 gaggggccga ctcacccggc gcggcgttga cagatgaggg gcaggctcga tttcggccgg

7381 cgacgtggag ctggccagcc tcgcaaatcg gcgaaaacgc ctgattttac gcgagtttcc

7441 cacagatgat gtggacaagc ctggggataa gtgccctgcg gtattgacac ttgaggggcg

7501 cgactactga cagatgaggg gcgcgatcct tgacacttga ggggcagagt gctgacagat

7561 gaggggcgca cctattgaca tttgaggggc tgtccacagg cagaaaatcc agcatttgca

7621 agggtttccg cccgtttttc ggccaccgct aacctgtctt ttaacctgct tttaaaccaa

7681 tatttataaa ccttgttttt aaccagggct gcgccctgtg cgcgtgaccg cgcacgccga

7741 aggggggtgc ccccccttct cgaaccctcc cggcccgcta acgcgggcct cccatccccc

7801 caggggctgc gcccctcggc cgcgaacggc ctcaccccaa aaatggcagc gctggccaat

7861 tcccgagtgc gcggaacccc tatttgttta tttttctaaa tacattcaaa tatgtatccg

7921 ctcatgagac aataaccctg ataaatgctt caataatatt gaaaaaggaa gagtatgagc

7981 catattcaac gggaaacgtc ttgctctagg ccgcgattaa attccaacat ggatgctgat

8041 ttatatgggt ataaatgggc tcgcgataat gtcgggcaat caggtgcgac aatctatcga

8101 ttgtatggga agcccgatgc gccagagttg tttctgaaac atggcaaagg tagcgttgcc

8161 aatgatgtta cagatgagat ggtcagacta aactggctga cggaatttat gcctcttccg

8221 accatcaagc attttatccg tactcctgat gatgcatggt tactcaccac tgcgatcccc

8281 gggaaaacag cattccaggt attagaagaa tatcctgatt caggtgaaaa tattgttgat

8341 gcgctggcag tgttcctgcg ccggttgcat tcgattcctg tttgtaattg tccttttaac

8401 agcgatcgcg tatttcgtct cgctcaggcg caatcacgaa tgaataacgg tttggttgat

8461 gcgagtgatt ttgatgacga gcgtaatggc tggcctgttg aacaagtctg gaaagaaatg

8521 cataaacttt tgccattctc accggattca gtcgtcactc atggtgattt ctcacttgat

8581 aaccttattt ttgacgaggg gaaattaata ggttgtattg atgttggacg agtcggaatc

8641 gcagaccgat accaggatct tgccatccta tggaactgcc tcggtgagtt ttctccttca

8701 ttacagaaac ggctttttca aaaatatggt attgataatc ctgatatgaa taaattgcag

8761 tttcatttga tgctcgatga gtttttctaa ctgtcagacc aagtttactc atatatactt

8821 tagattgatt taaaacttca tttttaattt aaaaggatct aggtgaagat cctttttgat

8881 aatctcatga ccaaaatccc ttaacgtgag ttttcgttcc actgagcgtc agaccccgta

8941 gaaaagatca aaggatcttc ttgagatcct ttttttctgc gcgtaatctg ctgcttgcaa

9001 acaaaaaaac caccgctacc agcggtggtt tgtttgccgg atcaagagct accaactctt

9061 tttccgaagg taactggctt cagcagagcg cagataccaa atactgtcct tctagtgtag

9121 ccgtagttag gccaccactt caagaactct gtagcaccgc ctacatacct cgctctgcta

9181 atcctgttac cagtggctgc tgccagtggc gataagtcgt gtcttaccgg gttggactca

9241 agacgatagt taccggataa ggcgcagcgg tcgggctgaa cggggggttc gtgcacacag

9301 cccagcttgg agcgaacgac ctacaccgaa ctgagatacc tacagcgtga gctatgagaa

9361 agcgccacgc ttcccgaagg gagaaaggcg gacaggtatc cggtaagcgg cagggtcgga

9421 acaggagagc gcacgaggga gcttccaggg ggaaacgcct ggtatcttta tagtcctgtc

9481 gggtttcgcc acctctgact tgagcgtcga tttttgtgat gctcgtcagg ggggcggagc

9541 ctatggaaaa acgccagcaa cgcggccttt ttacggttcc tggcagatcc tagatgtggc

9601 gcaacgatgc cggcgacaag caggagcgca ccgacttctt ccgcatcaag tgttttggct

9661 ctcaggccga ggcccacggc aagtatttgg gcaaggggtc gctggtattc gtgcagggca

9721 agattcggaa taccaagtac gagaaggacg gccagacggt ctacgggacc gacttcattg

9781 ccgataaggt ggattatctg gacaccaagg caccaggcgg gtcaaatcag gaataagggc

9841 acattgcccc ggcgtgagtc ggggcaatcc cgcaaggagg gtgaatgaat cggacgtttg

9901 accggaaggc atacaggcaa gaactgatcg acgcggggtt ttccgccgag gatgccgaaa

9961 ccatcgcaag ccgcaccgtc atgcgtgcgc cccgcgaaac cttccagtcc gtcggctcga

10021 tggtccagca agctacggcc aagatcgagc gcgacagcgt gcaactggct ccccctgccc

10081 tgcccgcgcc atcggccgcc gtggagcgtt cgcgtcgtct tgaacaggag gcggcaggtt

10141 tggcgaagtc gatgaccatc gacacgcgag gaactatgac gaccaagaag cgaaaaaccg

10201 ccggcgagga cctggcaaaa caggtcagcg aggccaagca ggccgcgttg ctgaaacaca

10261 cgaagcagca gatcaaggaa atgcagcttt ccttgttcga tattgcgccg tggccggaca

10321 cgatgcgagc gatgccaaac gacacggccc gctctgccct gttcaccacg cgcaacaaga

10381 aaatcccgcg cgaggcgctg caaaacaagg tcattttcca cgtcaacaag gacgtgaaga

10441 tcacctacac cggcgtcgag ctgcgggccg acgatgacga actggtgtgg cagcaggtgt

10501 tggagtacgc gaagcgcacc cctatcggcg agccgatcac cttcacgttc tacgagcttt

10561 gccaggacct gggctggtcg atcaatggcc ggtattacac gaaggccgag gaatgcctgt

10621 cgcgcctaca ggcgacggcg atgggcttca cgtccgaccg cgttgggcac ctggaatcgg

10681 tgtcgctgct gcaccgcttc cgcgtcctgg accgtggcaa gaaaacgtcc cgttgccagg

10741 tcctgatcga cgaggaaatc gtcgtgctgt ttgctggcga ccactacacg aaattcatat

10801 gggagaagta ccgcaagctg tcgccgacgg cccgacggat gttcgactat ttcagctcgc

10861 accgggagcc gtacccgctc aagctggaaa ccttccgcct catgtgcgga tcggattcca

10921 cccgcgtgaa gaagtggcgc gagcaggtcg gcgaagcctg cgaagagttg cgaggcagcg

10981 gcctggtgga acacgcctgg gtcaatgatg acctggtgca ttgcaaacgc tagggccttg

11041 tggggtcagt tccggctggg ggttcagcag cccctgctcg gatctgttgg accggacagt

11101 agtcatggtt gatgggctgc ctgtatcgag tggtgatttt gtgccgagct gccggtcggg

11161 gagctgttgg ctggctggtg gcaggatata ttgtggtgta aacaaattga cgcttagaca

11221 acttaataac acattgcgga cgtttttaat gta

//

>Targeted integration of mVenus on LHCBM1 locus

LOCUS Exported 6722 bp ds-DNA circular SYN 02-AUG-2024

DEFINITION synthetic circular DNA

ACCESSION .

VERSION .

KEYWORDS .

SOURCE synthetic DNA construct

ORGANISM recombinant plasmid

REFERENCE 1 (bases 1 to 6722)

AUTHORS Thomas Baier

TITLE Direct Submission

JOURNAL Exported Friday, Aug 2, 2024 from SnapGene Viewer 4.3.11

https://www.snapgene.com

FEATURES Location/Qualifiers

source 1..6722

/organism="recombinant plasmid"

/mol_type="other DNA"

misc_feature 50..56

/label=Insertion Event

misc_feature 64..110

/label=HA EcoRV

misc_feature 105..107

/label=PAM

misc_feature 108..127

/label=sgRNA1 rev

CDS 111..177

/codon_start=1

/label=LHCBM1

/translation="SDHLANPGTNNAFAYATKFTPQ"

CDS 178..195

/codon_start=1

/label=GSGSGS-Linker

/translation="GSGSGS"

CDS 196..313

/codon_start=1

/product="unnamed_input_seq"

/label=ext2A

/label=unnamed_input_seq__CDS

/translation="LLAIHPTEARHKQKIVAPVKQTLNFDLLKLAGDVESNPG"

CDS 316..333

/codon_start=1

/label=GSGSGS-Linker

/translation="GSGSGS"

CDS join(334..532,678..994,1140..1337)

/codon_start=1

/label=mVenus

/translation="VSKGEELFTGVVPILVELDGDVNGHKFSVSGEGEGDATYGKLTLK

LICTTGKLPVPWPTLVTTLGYGLQCFARYPDHMKQHDFFKSAMPEGYVQERTIFFKDDG

NYKTRAEVKFEGDTLVNRIELKGIDFKEDGNILGHKLEYNYNSHNVYITADKQKNGIKA

NFKIRHNIEDGGVQLADHYQQNTPIGDGPVLLPDNHYLSYQSKLSKDPNEKRDHMVLLE

FVTAAGITLGMDELYK"

intron 533..677

/label=rbcS2 intron 1

intron 995..1139

/label=rbcS2 intron 1

CDS 1338..1355

/codon_start=1

/label=GSGSGS-Linker

/translation="GSGSGS"

CDS join(1356..1613,1759..2112,2258..2431)

/codon_start=1

/label=AadA Spectinomycin resistance

/translation="REAVIAEVSTQLSEVVGVIERHLEPTLLAVHLYGSAVDGGLKPHS

DIDLLVTVTVRLDETTRRALINDLLETSASPGESEILRAVEVTIVVHDDIIPWRYPAKR

ELQFGEWQRNDILAGIFEPATIDIDLAILLTKAREHSVALVGPAAEELFDPVPEQDLFE

ALNETLTLWNSPPDWAGDERNVVLTLSRIWYSAVTGKIAPKDVAADWAMERLPAQYQPV

ILEARQAYLGQEEDRLASRADQLEEFVHYVKGEITKVVGK"

intron 1614..1758

/label=rbcS2 intron 1

intron 2113..2257

/label=rbcS2 intron 1

3'UTR 2435..2472

misc_feature 2435..2472

/label=HA2 EcoRV

misc_feature 2516..2540

/label=RB T-DNA repeat

/note="right border repeat from nopaline C58 T-DNA"

rep_origin 2627..3338

/label=oriV

/note="incP origin of replication"

promoter 3359..3463

/gene="bla"

/label=AmpR promoter

CDS 3464..4279

/codon_start=1

/gene="aph(3')-Ia"

/product="aminoglycoside phosphotransferase"

/label=KanR

/note="confers resistance to kanamycin in bacteria or G418

(Geneticin(R)) in eukaryotes"

/translation="MSHIQRETSCSRPRLNSNMDADLYGYKWARDNVGQSGATIYRLYG

KPDAPELFLKHGKGSVANDVTDEMVRLNWLTEFMPLPTIKHFIRTPDDAWLLTTAIPGK

TAFQVLEEYPDSGENIVDALAVFLRRLHSIPVCNCPFNSDRVFRLAQAQSRMNNGLVDA

SDFDDERNGWPVEQVWKEMHKLLPFSPDSVVTHGDFSLDNLIFDEGKLIGCIDVGRVGI

ADRYQDLAILWNCLGEFSPSLQKRLFQKYGIDNPDMNKLQFHLMLDEFF"

rep_origin 4450..5038

/direction=RIGHT

/label=ori

/note="high-copy-number ColE1/pMB1/pBR322/pUC origin of

replication"

CDS 5374..6522

/codon_start=1

/product="trans-acting replication protein that binds to

and activates oriV"

/label=trfA

/translation="MNRTFDRKAYRQELIDAGFSAEDAETIASRTVMRAPRETFQSVGS

MVQQATAKIERDSVQLAPPALPAPSAAVERSRRLEQEAAGLAKSMTIDTRGTMTTKKRK

TAGEDLAKQVSEAKQAALLKHTKQQIKEMQLSLFDIAPWPDTMRAMPNDTARSALFTTR

NKKIPREALQNKVIFHVNKDVKITYTGVELRADDDELVWQQVLEYAKRTPIGEPITFTF

YELCQDLGWSINGRYYTKAEECLSRLQATAMGFTSDRVGHLESVSLLHRFRVLDRGKKT

SRCQVLIDEEIVVLFAGDHYTKFIWEKYRKLSPTARRMFDYFSSHREPYPLKLETFRLM

CGSDSTRVKKWREQVGEACEELRGSGLVEHAWVNDDLVHCKR"

misc_feature 6668..6692

/label=LB T-DNA repeat

/note="left border repeat from nopaline C58 T-DNA"

ORIGIN

1 ttgcggacgt ttttaatgta ctggggttga aaatattcga tcgattgcct gaggcctgcc

61 gatatcttag tttacccggt gatgttatgc tcctcgtgcc cgctccacag gtccgaccac

121 ctggccaacc ccggcaccaa caacgccttc gcctacgcca ccaagttcac cccccagggc

181 agcggcagcg gcagcctgct ggccatccac cccaccgagg cccgccacaa gcagaagatc

241 gtggcccccg tgaagcagac cctgaacttc gacctgctga agctggccgg cgacgtggag

301 agcaaccccg gccccggcag cggcagcggc agcgtgagca agggcgagga gctgttcacc

361 ggcgtggtgc ccatcctggt ggagctggac ggcgacgtga acggccacaa gttcagcgtg

421 agcggcgagg gcgagggcga cgccacctac ggcaagctga ccctgaagct gatctgcacc

481 accggcaagc tgcccgtgcc ctggcccacc ctggtgacca ccctgggcta cggtgagtcg

541 acgagcaagc ccggcggatc aggcagcgtg cttgcagatt tgacttgcaa cgcccgcatt

601 gtgtcgacga aggcttttgg ctcctctgtc gctgtctcaa gcagcatcta accctgcgtc

661 gccgtttcca tttgcaggcc tgcagtgctt cgcccgctac cccgaccaca tgaagcagca

721 cgacttcttc aagagcgcca tgcccgaggg ctacgtgcag gagcgcacca tcttcttcaa

781 ggacgacggt aactacaaga cccgcgccga ggtgaagttc gagggcgaca ccctggtgaa

841 ccgcatcgag ctgaagggca tcgacttcaa ggaggacggc aacatcctgg gccacaagct

901 ggagtacaac tacaacagcc acaacgtgta catcaccgcc gacaagcaga agaacggcat

961 caaggccaac ttcaagatcc gccacaacat cgaggtgagt cgacgagcaa gcccggcgga

1021 tcaggcagcg tgcttgcaga tttgacttgc aacgcccgca ttgtgtcgac gaaggctttt

1081 ggctcctctg tcgctgtctc aagcagcatc taaccctgcg tcgccgtttc catttgcagg

1141 acggcggcgt gcagctggcc gaccactacc agcagaacac ccccatcggc gacggccccg

1201 tgctgctgcc cgacaaccac tacctgagct accagagcaa gctgagcaag gaccccaacg

1261 agaagcgcga ccacatggtg ctgctggagt tcgtgaccgc cgccggcatc accctgggca

1321 tggacgagct gtacaagggc agcggcagcg gcagccgcga ggccgtgatc gccgaggtga

1381 gcacccagct gagcgaggtg gtgggcgtga tcgagcgcca cctggagccc accctgctgg

1441 ccgtgcacct gtacggcagc gccgtggacg gcggcctgaa gccccacagc gacatcgacc

1501 tgctggtgac cgtgaccgtg cgcctggacg agacgacccg ccgcgccctg atcaacgacc

1561 tgctggagac gagcgccagc cccggcgaga gcgagatcct gcgcgccgtg gaggtgagtc

1621 gacgagcaag cccggcggat caggcagcgt gcttgcagat ttgacttgca acgcccgcat

1681 tgtgtcgacg aaggcttttg gctcctctgt cgctgtctca agcagcatct aaccctgcgt

1741 cgccgtttcc atttgcaggt gaccatcgtg gtgcacgacg acatcatccc ctggcgctac

1801 cccgccaagc gcgagctgca gttcggcgag tggcagcgca acgacatcct ggccggcatc

1861 ttcgagcccg ccaccatcga catcgacctg gccatcctgc tgaccaaggc ccgcgagcac

1921 agcgtggccc tggtgggccc cgccgccgag gagctgttcg accccgtgcc cgagcaggac

1981 ctgttcgagg ccctgaacga gacgctgacc ctgtggaaca gcccccccga ctgggccggc

2041 gacgagcgca acgtggtgct gaccctgagc cgcatctggt acagcgccgt gaccggcaag

2101 atcgccccca aggtgagtcg acgagcaagc ccggcggatc aggcagcgtg cttgcagatt

2161 tgacttgcaa cgcccgcatt gtgtcgacga aggcttttgg ctcctctgtc gctgtctcaa

2221 gcagcatcta accctgcgtc gccgtttcca tttgcaggac gtggccgccg actgggccat

2281 ggagcgcctg cccgcccagt accagcccgt gatcctggag gcccgccagg cctacctggg

2341 ccaggaggag gaccgcctgg ccagccgcgc cgaccagctg gaggagttcg tgcactacgt

2401 gaagggcgag atcaccaagg tggtgggcaa gtaaatgccc tggcggcaca gttttgatgt

2461 accaataggg atatcgggat acgatcgaat attatccgtt taaactatca gtgtttgaca

2521 ggatatattg gcgggtaaac ctaagagaaa agagcgttta ttagaataat cggatattta

2581 aaagggcgtg aaaaggttta tccgttcgtc catttgtatg tgccagccgc ctttgcgacg

2641 ctcaccgggc tggttgccct cgccgctggg ctggcggccg tctatggccc tgcaaacgcg

2701 ccagaaacgc cgtcgaagcc gtgtgcgaga caccgcggcc gccggcgttg tggatacctc

2761 gcggaaaact tggccctcac tgacagatga ggggcggacg ttgacacttg aggggccgac

2821 tcacccggcg cggcgttgac agatgagggg caggctcgat ttcggccggc gacgtggagc

2881 tggccagcct cgcaaatcgg cgaaaacgcc tgattttacg cgagtttccc acagatgatg

2941 tggacaagcc tggggataag tgccctgcgg tattgacact tgaggggcgc gactactgac

3001 agatgagggg cgcgatcctt gacacttgag gggcagagtg ctgacagatg aggggcgcac

3061 ctattgacat ttgaggggct gtccacaggc agaaaatcca gcatttgcaa gggtttccgc

3121 ccgtttttcg gccaccgcta acctgtcttt taacctgctt ttaaaccaat atttataaac

3181 cttgttttta accagggctg cgccctgtgc gcgtgaccgc gcacgccgaa ggggggtgcc

3241 cccccttctc gaaccctccc ggcccgctaa cgcgggcctc ccatcccccc aggggctgcg

3301 cccctcggcc gcgaacggcc tcaccccaaa aatggcagcg ctggccaatt cccgagtgcg

3361 cggaacccct atttgtttat ttttctaaat acattcaaat atgtatccgc tcatgagaca

3421 ataaccctga taaatgcttc aataatattg aaaaaggaag agtatgagcc atattcaacg

3481 ggaaacgtct tgctctaggc cgcgattaaa ttccaacatg gatgctgatt tatatgggta

3541 taaatgggct cgcgataatg tcgggcaatc aggtgcgaca atctatcgat tgtatgggaa

3601 gcccgatgcg ccagagttgt ttctgaaaca tggcaaaggt agcgttgcca atgatgttac

3661 agatgagatg gtcagactaa actggctgac ggaatttatg cctcttccga ccatcaagca

3721 ttttatccgt actcctgatg atgcatggtt actcaccact gcgatccccg ggaaaacagc

3781 attccaggta ttagaagaat atcctgattc aggtgaaaat attgttgatg cgctggcagt

3841 gttcctgcgc cggttgcatt cgattcctgt ttgtaattgt ccttttaaca gcgatcgcgt

3901 atttcgtctc gctcaggcgc aatcacgaat gaataacggt ttggttgatg cgagtgattt

3961 tgatgacgag cgtaatggct ggcctgttga acaagtctgg aaagaaatgc ataaactttt

4021 gccattctca ccggattcag tcgtcactca tggtgatttc tcacttgata accttatttt

4081 tgacgagggg aaattaatag gttgtattga tgttggacga gtcggaatcg cagaccgata

4141 ccaggatctt gccatcctat ggaactgcct cggtgagttt tctccttcat tacagaaacg

4201 gctttttcaa aaatatggta ttgataatcc tgatatgaat aaattgcagt ttcatttgat

4261 gctcgatgag tttttctaac tgtcagacca agtttactca tatatacttt agattgattt

4321 aaaacttcat ttttaattta aaaggatcta ggtgaagatc ctttttgata atctcatgac

4381 caaaatccct taacgtgagt tttcgttcca ctgagcgtca gaccccgtag aaaagatcaa

4441 aggatcttct tgagatcctt tttttctgcg cgtaatctgc tgcttgcaaa caaaaaaacc

4501 accgctacca gcggtggttt gtttgccgga tcaagagcta ccaactcttt ttccgaaggt

4561 aactggcttc agcagagcgc agataccaaa tactgtcctt ctagtgtagc cgtagttagg

4621 ccaccacttc aagaactctg tagcaccgcc tacatacctc gctctgctaa tcctgttacc

4681 agtggctgct gccagtggcg ataagtcgtg tcttaccggg ttggactcaa gacgatagtt

4741 accggataag gcgcagcggt cgggctgaac ggggggttcg tgcacacagc ccagcttgga

4801 gcgaacgacc tacaccgaac tgagatacct acagcgtgag ctatgagaaa gcgccacgct

4861 tcccgaaggg agaaaggcgg acaggtatcc ggtaagcggc agggtcggaa caggagagcg

4921 cacgagggag cttccagggg gaaacgcctg gtatctttat agtcctgtcg ggtttcgcca

4981 cctctgactt gagcgtcgat ttttgtgatg ctcgtcaggg gggcggagcc tatggaaaaa

5041 cgccagcaac gcggcctttt tacggttcct ggcagatcct agatgtggcg caacgatgcc

5101 ggcgacaagc aggagcgcac cgacttcttc cgcatcaagt gttttggctc tcaggccgag

5161 gcccacggca agtatttggg caaggggtcg ctggtattcg tgcagggcaa gattcggaat

5221 accaagtacg agaaggacgg ccagacggtc tacgggaccg acttcattgc cgataaggtg

5281 gattatctgg acaccaaggc accaggcggg tcaaatcagg aataagggca cattgccccg

5341 gcgtgagtcg gggcaatccc gcaaggaggg tgaatgaatc ggacgtttga ccggaaggca

5401 tacaggcaag aactgatcga cgcggggttt tccgccgagg atgccgaaac catcgcaagc

5461 cgcaccgtca tgcgtgcgcc ccgcgaaacc ttccagtccg tcggctcgat ggtccagcaa

5521 gctacggcca agatcgagcg cgacagcgtg caactggctc cccctgccct gcccgcgcca

5581 tcggccgccg tggagcgttc gcgtcgtctt gaacaggagg cggcaggttt ggcgaagtcg

5641 atgaccatcg acacgcgagg aactatgacg accaagaagc gaaaaaccgc cggcgaggac

5701 ctggcaaaac aggtcagcga ggccaagcag gccgcgttgc tgaaacacac gaagcagcag

5761 atcaaggaaa tgcagctttc cttgttcgat attgcgccgt ggccggacac gatgcgagcg

5821 atgccaaacg acacggcccg ctctgccctg ttcaccacgc gcaacaagaa aatcccgcgc

5881 gaggcgctgc aaaacaaggt cattttccac gtcaacaagg acgtgaagat cacctacacc

5941 ggcgtcgagc tgcgggccga cgatgacgaa ctggtgtggc agcaggtgtt ggagtacgcg

6001 aagcgcaccc ctatcggcga gccgatcacc ttcacgttct acgagctttg ccaggacctg

6061 ggctggtcga tcaatggccg gtattacacg aaggccgagg aatgcctgtc gcgcctacag

6121 gcgacggcga tgggcttcac gtccgaccgc gttgggcacc tggaatcggt gtcgctgctg

6181 caccgcttcc gcgtcctgga ccgtggcaag aaaacgtccc gttgccaggt cctgatcgac

6241 gaggaaatcg tcgtgctgtt tgctggcgac cactacacga aattcatatg ggagaagtac

6301 cgcaagctgt cgccgacggc ccgacggatg ttcgactatt tcagctcgca ccgggagccg

6361 tacccgctca agctggaaac cttccgcctc atgtgcggat cggattccac ccgcgtgaag

6421 aagtggcgcg agcaggtcgg cgaagcctgc gaagagttgc gaggcagcgg cctggtggaa

6481 cacgcctggg tcaatgatga cctggtgcat tgcaaacgct agggccttgt ggggtcagtt

6541 ccggctgggg gttcagcagc ccctgctcgg atctgttgga ccggacagta gtcatggttg

6601 atgggctgcc tgtatcgagt ggtgattttg tgccgagctg ccggtcgggg agctgttggc

6661 tggctggtgg caggatatat tgtggtgtaa acaaattgac gcttagacaa cttaataaca

6721 ca

//

>Targeted integration of CnVs on LHCBM1 locus

LOCUS Exported 8207 bp ds-DNA circular SYN 02-AUG-2024

DEFINITION synthetic circular DNA

ACCESSION .

VERSION .

KEYWORDS .

SOURCE synthetic DNA construct

ORGANISM recombinant plasmid

REFERENCE 1 (bases 1 to 8207)

AUTHORS Trial User

TITLE Direct Submission

JOURNAL Exported Friday, Aug 2, 2024 from SnapGene Viewer 4.3.11

https://www.snapgene.com

FEATURES Location/Qualifiers

source 1..8207

/organism="recombinant plasmid"

/mol_type="other DNA"

misc_feature 37..43

/label=Insertion Event

misc_feature 51..97

/label=HA1 EcoRV

misc_feature 92..94

/label=PAM

misc_feature 95..114

/label=sgRNA1 rev

CDS 98..164

/codon_start=1

/label=LHCBM1

/translation="SDHLANPGTNNAFAYATKFTPQ"

CDS 165..182

/codon_start=1

/label=GSGSGS-Linker

/translation="GSGSGS"

CDS 183..300

/codon_start=1

/product="unnamed_input_seq"

/label=ext2A

/label=unnamed_input_seq__CDS

/translation="LLAIHPTEARHKQKIVAPVKQTLNFDLLKLAGDVESNPG"

CDS 303..320

/codon_start=1

/label=GSGSGS-Linker

/translation="GSGSGS"

CDS join(321..418,564..951,1097..1498,1644..2055,2201..2587,

2733..2809)

/codon_start=1

/product="AFN21429.1 terpene synthase Valencene synthase

[Callitropsis nootkatensis] Beekwilder 2014"

/label=AFN21429.1 terpene synthase Valencene synthase

/label=AFN21429.1 terpene synthase Valencene synthase

[Callitropsis nootkatensis] Beekwilder 2014__CDS

/translation="AEMFNGNSSNDGSSCMPVKDALRRTGNHHPNLWTDDFIQSLNSPY

SDSSYHKHREILIDEIRDMFSNGEGDEFGVLENIWFVDVVQRLGIDRHFQEEIKTALDY

IYKFWNHDSIFGDLNMVALGFRILRLNRYVASSDVFKKFKGEEGQFSGFESSDQDAKLE

MMLNLYKASELDFPDEDILKEARAFASMYLKHVIKEYGDIQESKNPLLMEIEYTFKYPW

RCRLPRLEAWNFIHIMRQQDCNISLANNLYKIPKIYMKKILELAILDFNILQSQHQHEM

KLISTWWKNSSAIQLDFFRHRHIESYFWWASPLFEPEFSTCRINCTKLSTKMFLLDDIY

DTYGTVEELKPFTTTLTRWDVSTVDNHPDYMKIAFNFSYEIYKEIASEAERKHGPFVYK

YLQSCWKSYIEAYMQEAEWIASNHIPGFDEYLMNGVKSSGMRILMIHALILMDTPLSDE

ILEQLDIPSSKSQALLSLITRLVDDVKDFEDEQAHGEMASSIECYMKDNHGSTREDALN

YLKIRIESCVQELNKELLEPSNMHGSFRNLYLNVGMRVIFFMLNDGDLFTHSNRKEIQD

AITKFFVEPIIP"

intron 419..563

/label=intron

intron 952..1096

/label=intron

intron 1499..1643

/label=intron

unsure 1988^1989

/label=bis hier hin sequenziert

intron 2056..2200

/label=intron

intron 2588..2732

/label=intron

CDS 2810..2827

/codon_start=1

/label=GSGSGS-Linker

/translation="GSGSGS"

CDS join(2828..3085,3231..3584,3730..3903)

/codon_start=1

/label=AadA Spectinomycin resistance

/translation="REAVIAEVSTQLSEVVGVIERHLEPTLLAVHLYGSAVDGGLKPHS

DIDLLVTVTVRLDETTRRALINDLLETSASPGESEILRAVEVTIVVHDDIIPWRYPAKR

ELQFGEWQRNDILAGIFEPATIDIDLAILLTKAREHSVALVGPAAEELFDPVPEQDLFE

ALNETLTLWNSPPDWAGDERNVVLTLSRIWYSAVTGKIAPKDVAADWAMERLPAQYQPV

ILEARQAYLGQEEDRLASRADQLEEFVHYVKGEITKVVGK"

intron 3086..3230

/label=rbcS2 intron 1

intron 3585..3729

/label=rbcS2 intron 1

3'UTR 3907..3944

misc_feature 3907..3944

/label=HA2 EcoRV

misc_feature 3988..4012

/label=RB T-DNA repeat

/note="right border repeat from nopaline C58 T-DNA"

rep_origin 4099..4810

/label=oriV

/note="incP origin of replication"

promoter 4831..4935

/gene="bla"

/label=AmpR promoter

CDS 4936..5751

/codon_start=1

/gene="aph(3')-Ia"

/product="aminoglycoside phosphotransferase"

/label=KanR

/note="confers resistance to kanamycin in bacteria or G418

(Geneticin(R)) in eukaryotes"

/translation="MSHIQRETSCSRPRLNSNMDADLYGYKWARDNVGQSGATIYRLYG

KPDAPELFLKHGKGSVANDVTDEMVRLNWLTEFMPLPTIKHFIRTPDDAWLLTTAIPGK

TAFQVLEEYPDSGENIVDALAVFLRRLHSIPVCNCPFNSDRVFRLAQAQSRMNNGLVDA

SDFDDERNGWPVEQVWKEMHKLLPFSPDSVVTHGDFSLDNLIFDEGKLIGCIDVGRVGI

ADRYQDLAILWNCLGEFSPSLQKRLFQKYGIDNPDMNKLQFHLMLDEFF"

rep_origin 5922..6510

/direction=RIGHT

/label=ori

/note="high-copy-number ColE1/pMB1/pBR322/pUC origin of

replication"

CDS 6846..7994

/codon_start=1

/product="trans-acting replication protein that binds to

and activates oriV"

/label=trfA

/translation="MNRTFDRKAYRQELIDAGFSAEDAETIASRTVMRAPRETFQSVGS

MVQQATAKIERDSVQLAPPALPAPSAAVERSRRLEQEAAGLAKSMTIDTRGTMTTKKRK

TAGEDLAKQVSEAKQAALLKHTKQQIKEMQLSLFDIAPWPDTMRAMPNDTARSALFTTR

NKKIPREALQNKVIFHVNKDVKITYTGVELRADDDELVWQQVLEYAKRTPIGEPITFTF

YELCQDLGWSINGRYYTKAEECLSRLQATAMGFTSDRVGHLESVSLLHRFRVLDRGKKT

SRCQVLIDEEIVVLFAGDHYTKFIWEKYRKLSPTARRMFDYFSSHREPYPLKLETFRLM

CGSDSTRVKKWREQVGEACEELRGSGLVEHAWVNDDLVHCKR"

misc_feature 8140..8164

/label=LB T-DNA repeat

/note="left border repeat from nopaline C58 T-DNA"

ORIGIN

1 taatgtactg gggttgaaaa tattcgatcg attgcctgag gcctgccgat atcttagttt

61 acccggtgat gttatgctcc tcgtgcccgc tccacaggtc cgaccacctg gccaaccccg

121 gcaccaacaa cgccttcgcc tacgccacca agttcacccc ccagggcagc ggcagcggca

181 gcctgctggc catccacccc accgaggccc gccacaagca gaagatcgtg gcccccgtga

241 agcagaccct gaacttcgac ctgctgaagc tggccggcga cgtggagagc aaccccggcc

301 ccggcagcgg cagcggcagc gccgagatgt tcaacggcaa cagcagcaac gacggcagca

361 gctgcatgcc cgtgaaggac gccctgcgcc gcaccggcaa ccaccacccc aacctgtggt

421 gagtcgacga gcaagcccgg cggatcaggc agcgtgcttg cagatttgac ttgcaacgcc

481 cgcattgtgt cgacgaaggc ttttggctcc tctgtcgctg tctcaagcag catctaaccc

541 tgcgtcgccg tttccatttg caggaccgac gacttcatcc agagcctgaa cagcccctac

601 agcgacagca gctaccacaa gcaccgcgag atcctgatcg acgagatccg cgacatgttc

661 agcaacggcg agggcgacga gttcggcgtg ctggagaaca tctggttcgt ggacgtggtg

721 cagcgcctgg gcatcgaccg ccacttccag gaggagatca agaccgccct ggactacatc

781 tacaagttct ggaaccacga cagcatcttc ggcgacctga acatggtggc cctgggcttc

841 cgcatcctgc gcctgaaccg ctacgtggcc agcagcgacg tgttcaagaa gttcaagggc

901 gaggagggcc agttcagcgg cttcgagagc agcgaccagg acgccaagct ggtgagtcga

961 cgagcaagcc cggcggatca ggcagcgtgc ttgcagattt gacttgcaac gcccgcattg

1021 tgtcgacgaa ggcttttggc tcctctgtcg ctgtctcaag cagcatctaa ccctgcgtcg

1081 ccgtttccat ttgcaggaga tgatgctgaa cctgtacaag gccagcgagc tggacttccc

1141 cgacgaggac atcctgaagg aggcccgcgc cttcgccagc atgtacctga agcacgtgat

1201 caaggagtac ggcgacatcc aggagagcaa gaaccccctg ctgatggaga tcgagtacac

1261 cttcaagtac ccctggcgct gccgcctgcc ccgcctggag gcctggaact tcatccacat

1321 catgcgccag caggactgca acatcagcct ggccaacaac ctgtacaaga tccccaagat

1381 ttacatgaag aagatcctgg agctggccat cctggacttc aacatcctgc agagccagca

1441 ccagcacgag atgaagctga tcagcacctg gtggaagaac agcagcgcca tccagctggt

1501 gagtcgacga gcaagcccgg cggatcaggc agcgtgcttg cagatttgac ttgcaacgcc

1561 cgcattgtgt cgacgaaggc ttttggctcc tctgtcgctg tctcaagcag catctaaccc

1621 tgcgtcgccg tttccatttg caggacttct tccgccaccg ccacatcgag agctacttct

1681 ggtgggccag ccccctgttc gagcccgagt tcagcacctg ccgcatcaac tgcaccaagc

1741 tgagcaccaa gatgttcctg ctggacgaca tctacgacac ctacggcacc gtggaggagc

1801 tgaagccctt caccaccacc ctgacccgct gggacgtgag caccgtggac aaccaccccg

1861 actacatgaa gatcgccttc aacttcagct acgagattta caaggagatc gccagcgagg

1921 ccgagcgcaa gcacggcccc ttcgtgtaca agtacctgca gagctgctgg aagagctaca

1981 tcgaggccta catgcaggag gccgagtgga tcgccagcaa ccacatcccc ggcttcgacg

2041 agtacctgat gaacggtgag tcgacgagca agcccggcgg atcaggcagc gtgcttgcag

2101 atttgacttg caacgcccgc attgtgtcga cgaaggcttt tggctcctct gtcgctgtct

2161 caagcagcat ctaaccctgc gtcgccgttt ccatttgcag gcgtgaagag cagcggcatg

2221 cgcatcctga tgatccacgc cctgatcctg atggacaccc ccctgagcga cgagatcctg

2281 gagcagctgg acatccccag cagcaagagc caggccctgc tgagcctgat cacccgcctg

2341 gtggacgacg tgaaggactt cgaggacgag caggcccacg gcgagatggc cagcagcatc

2401 gagtgctaca tgaaggacaa ccacggcagc acccgcgagg acgccctgaa ctacctgaag

2461 atccgcatcg agagctgcgt gcaggagctg aacaaggagc tgctggagcc cagcaacatg

2521 cacggcagct tccgcaacct gtacctgaac gtgggcatgc gcgtgatctt cttcatgctg

2581 aacgacggtg agtcgacgag caagcccggc ggatcaggca gcgtgcttgc agatttgact

2641 tgcaacgccc gcattgtgtc gacgaaggct tttggctcct ctgtcgctgt ctcaagcagc

2701 atctaaccct gcgtcgccgt ttccatttgc aggcgacctg ttcacccaca gcaaccgcaa

2761 ggagatccag gacgccatca ccaagttctt cgtggagccc atcatccccg gcagcggcag

2821 cggcagccgc gaggccgtga tcgccgaggt gagcacccag ctgagcgagg tggtgggcgt

2881 gatcgagcgc cacctggagc ccaccctgct ggccgtgcac ctgtacggca gcgccgtgga

2941 cggcggcctg aagccccaca gcgacatcga cctgctggtg accgtgaccg tgcgcctgga

3001 cgagacgacc cgccgcgccc tgatcaacga cctgctggag acgagcgcca gccccggcga

3061 gagcgagatc ctgcgcgccg tggaggtgag tcgacgagca agcccggcgg atcaggcagc

3121 gtgcttgcag atttgacttg caacgcccgc attgtgtcga cgaaggcttt tggctcctct

3181 gtcgctgtct caagcagcat ctaaccctgc gtcgccgttt ccatttgcag gtgaccatcg

3241 tggtgcacga cgacatcatc ccctggcgct accccgccaa gcgcgagctg cagttcggcg

3301 agtggcagcg caacgacatc ctggccggca tcttcgagcc cgccaccatc gacatcgacc

3361 tggccatcct gctgaccaag gcccgcgagc acagcgtggc cctggtgggc cccgccgccg

3421 aggagctgtt cgaccccgtg cccgagcagg acctgttcga ggccctgaac gagacgctga

3481 ccctgtggaa cagccccccc gactgggccg gcgacgagcg caacgtggtg ctgaccctga

3541 gccgcatctg gtacagcgcc gtgaccggca agatcgcccc caaggtgagt cgacgagcaa

3601 gcccggcgga tcaggcagcg tgcttgcaga tttgacttgc aacgcccgca ttgtgtcgac

3661 gaaggctttt ggctcctctg tcgctgtctc aagcagcatc taaccctgcg tcgccgtttc

3721 catttgcagg acgtggccgc cgactgggcc atggagcgcc tgcccgccca gtaccagccc

3781 gtgatcctgg aggcccgcca ggcctacctg ggccaggagg aggaccgcct ggccagccgc

3841 gccgaccagc tggaggagtt cgtgcactac gtgaagggcg agatcaccaa ggtggtgggc

3901 aagtaaatgc cctggcggca cagttttgat gtaccaatag ggatatcggg atacgatcga

3961 atattatccg tttaaactat cagtgtttga caggatatat tggcgggtaa acctaagaga

4021 aaagagcgtt tattagaata atcggatatt taaaagggcg tgaaaaggtt tatccgttcg

4081 tccatttgta tgtgccagcc gcctttgcga cgctcaccgg gctggttgcc ctcgccgctg

4141 ggctggcggc cgtctatggc cctgcaaacg cgccagaaac gccgtcgaag ccgtgtgcga

4201 gacaccgcgg ccgccggcgt tgtggatacc tcgcggaaaa cttggccctc actgacagat

4261 gaggggcgga cgttgacact tgaggggccg actcacccgg cgcggcgttg acagatgagg

4321 ggcaggctcg atttcggccg gcgacgtgga gctggccagc ctcgcaaatc ggcgaaaacg

4381 cctgatttta cgcgagtttc ccacagatga tgtggacaag cctggggata agtgccctgc

4441 ggtattgaca cttgaggggc gcgactactg acagatgagg ggcgcgatcc ttgacacttg

4501 aggggcagag tgctgacaga tgaggggcgc acctattgac atttgagggg ctgtccacag

4561 gcagaaaatc cagcatttgc aagggtttcc gcccgttttt cggccaccgc taacctgtct

4621 tttaacctgc ttttaaacca atatttataa accttgtttt taaccagggc tgcgccctgt

4681 gcgcgtgacc gcgcacgccg aaggggggtg cccccccttc tcgaaccctc ccggcccgct

4741 aacgcgggcc tcccatcccc ccaggggctg cgcccctcgg ccgcgaacgg cctcacccca

4801 aaaatggcag cgctggccaa ttcccgagtg cgcggaaccc ctatttgttt atttttctaa

4861 atacattcaa atatgtatcc gctcatgaga caataaccct gataaatgct tcaataatat

4921 tgaaaaagga agagtatgag ccatattcaa cgggaaacgt cttgctctag gccgcgatta

4981 aattccaaca tggatgctga tttatatggg tataaatggg ctcgcgataa tgtcgggcaa

5041 tcaggtgcga caatctatcg attgtatggg aagcccgatg cgccagagtt gtttctgaaa

5101 catggcaaag gtagcgttgc caatgatgtt acagatgaga tggtcagact aaactggctg

5161 acggaattta tgcctcttcc gaccatcaag cattttatcc gtactcctga tgatgcatgg

5221 ttactcacca ctgcgatccc cgggaaaaca gcattccagg tattagaaga atatcctgat

5281 tcaggtgaaa atattgttga tgcgctggca gtgttcctgc gccggttgca ttcgattcct

5341 gtttgtaatt gtccttttaa cagcgatcgc gtatttcgtc tcgctcaggc gcaatcacga

5401 atgaataacg gtttggttga tgcgagtgat tttgatgacg agcgtaatgg ctggcctgtt

5461 gaacaagtct ggaaagaaat gcataaactt ttgccattct caccggattc agtcgtcact

5521 catggtgatt tctcacttga taaccttatt tttgacgagg ggaaattaat aggttgtatt

5581 gatgttggac gagtcggaat cgcagaccga taccaggatc ttgccatcct atggaactgc

5641 ctcggtgagt tttctccttc attacagaaa cggctttttc aaaaatatgg tattgataat

5701 cctgatatga ataaattgca gtttcatttg atgctcgatg agtttttcta actgtcagac

5761 caagtttact catatatact ttagattgat ttaaaacttc atttttaatt taaaaggatc

5821 taggtgaaga tcctttttga taatctcatg accaaaatcc cttaacgtga gttttcgttc

5881 cactgagcgt cagaccccgt agaaaagatc aaaggatctt cttgagatcc tttttttctg

5941 cgcgtaatct gctgcttgca aacaaaaaaa ccaccgctac cagcggtggt ttgtttgccg

6001 gatcaagagc taccaactct ttttccgaag gtaactggct tcagcagagc gcagatacca

6061 aatactgtcc ttctagtgta gccgtagtta ggccaccact tcaagaactc tgtagcaccg

6121 cctacatacc tcgctctgct aatcctgtta ccagtggctg ctgccagtgg cgataagtcg

6181 tgtcttaccg ggttggactc aagacgatag ttaccggata aggcgcagcg gtcgggctga

6241 acggggggtt cgtgcacaca gcccagcttg gagcgaacga cctacaccga actgagatac

6301 ctacagcgtg agctatgaga aagcgccacg cttcccgaag ggagaaaggc ggacaggtat

6361 ccggtaagcg gcagggtcgg aacaggagag cgcacgaggg agcttccagg gggaaacgcc

6421 tggtatcttt atagtcctgt cgggtttcgc cacctctgac ttgagcgtcg atttttgtga

6481 tgctcgtcag gggggcggag cctatggaaa aacgccagca acgcggcctt tttacggttc

6541 ctggcagatc ctagatgtgg cgcaacgatg ccggcgacaa gcaggagcgc accgacttct

6601 tccgcatcaa gtgttttggc tctcaggccg aggcccacgg caagtatttg ggcaaggggt

6661 cgctggtatt cgtgcagggc aagattcgga ataccaagta cgagaaggac ggccagacgg

6721 tctacgggac cgacttcatt gccgataagg tggattatct ggacaccaag gcaccaggcg

6781 ggtcaaatca ggaataaggg cacattgccc cggcgtgagt cggggcaatc ccgcaaggag

6841 ggtgaatgaa tcggacgttt gaccggaagg catacaggca agaactgatc gacgcggggt

6901 tttccgccga ggatgccgaa accatcgcaa gccgcaccgt catgcgtgcg ccccgcgaaa

6961 ccttccagtc cgtcggctcg atggtccagc aagctacggc caagatcgag cgcgacagcg

7021 tgcaactggc tccccctgcc ctgcccgcgc catcggccgc cgtggagcgt tcgcgtcgtc

7081 ttgaacagga ggcggcaggt ttggcgaagt cgatgaccat cgacacgcga ggaactatga

7141 cgaccaagaa gcgaaaaacc gccggcgagg acctggcaaa acaggtcagc gaggccaagc

7201 aggccgcgtt gctgaaacac acgaagcagc agatcaagga aatgcagctt tccttgttcg

7261 atattgcgcc gtggccggac acgatgcgag cgatgccaaa cgacacggcc cgctctgccc

7321 tgttcaccac gcgcaacaag aaaatcccgc gcgaggcgct gcaaaacaag gtcattttcc

7381 acgtcaacaa ggacgtgaag atcacctaca ccggcgtcga gctgcgggcc gacgatgacg

7441 aactggtgtg gcagcaggtg ttggagtacg cgaagcgcac ccctatcggc gagccgatca

7501 ccttcacgtt ctacgagctt tgccaggacc tgggctggtc gatcaatggc cggtattaca

7561 cgaaggccga ggaatgcctg tcgcgcctac aggcgacggc gatgggcttc acgtccgacc

7621 gcgttgggca cctggaatcg gtgtcgctgc tgcaccgctt ccgcgtcctg gaccgtggca

7681 agaaaacgtc ccgttgccag gtcctgatcg acgaggaaat cgtcgtgctg tttgctggcg

7741 accactacac gaaattcata tgggagaagt accgcaagct gtcgccgacg gcccgacgga

7801 tgttcgacta tttcagctcg caccgggagc cgtacccgct caagctggaa accttccgcc

7861 tcatgtgcgg atcggattcc acccgcgtga agaagtggcg cgagcaggtc ggcgaagcct

7921 gcgaagagtt gcgaggcagc ggcctggtgg aacacgcctg ggtcaatgat gacctggtgc

7981 attgcaaacg ctagggcctt gtggggtcag ttccggctgg gggttcagca gcccctgctc

8041 ggatctgttg gaccggacag tagtcatggt tgatgggctg cctgtatcga gtggtgattt

8101 tgtgccgagc tgccggtcgg ggagctgttg gctggctggt ggcaggatat attgtggtgt

8161 aaacaaattg acgcttagac aacttaataa cacattgcgg acgtttt

//l expression of mVenus

LOCUS Exported 8364 bp ds-DNA circular SYN 02-AUG-2024

DEFINITION synthetic circular DNA

ACCESSION .

VERSION .

KEYWORDS .

SOURCE synthetic DNA construct

ORGANISM recombinant plasmid

REFERENCE 1 (bases 1 to 8364)

AUTHORS Thomas Baier

TITLE Direct Submission

JOURNAL Exported Friday, Aug 2, 2024 from SnapGene Viewer 4.3.11

https://www.snapgene.com

FEATURES Location/Qualifiers

source 1..8364

/organism="recombinant plasmid"

/mol_type="other DNA"

misc_feature 31..37

/label=Insertion Event

misc_feature 54..57

/label=fusion site

promoter 58..324

/label=HSP70Ap promoter

/label=HSP70Ap

promoter 331..522

/label=P-bTUB2

5'UTR join(523..598,744..798)

/label=5UTR bTUB2

intron 599..743

/label=RBCS2i

/label=RBCS2i(1)

misc_feature 799..802

/label=fusion site

CDS 803..820

/label=GSGSGS-Linker

gene join(821..1019,1165..1481,1627..1824)

/label=mVenus

intron 1020..1164

/label=rbcS2 intron 1

intron 1482..1626

/label=rbcS2 intron 1

CDS 1825..1842

/codon_start=1

/label=GSGSGS-Linker

/translation="GSGSGS"

misc_feature 1843..1846

/label=fusion site

CDS 1849..1866

/codon_start=1

/label=GSGSGS-Linker

/translation="GSGSGS"

CDS join(1867..2124,2270..2623,2769..2942)

/codon_start=1

/label=AadA Spectinomycin resistance

/translation="REAVIAEVSTQLSEVVGVIERHLEPTLLAVHLYGSAVDGGLKPHS

DIDLLVTVTVRLDETTRRALINDLLETSASPGESEILRAVEVTIVVHDDIIPWRYPAKR

ELQFGEWQRNDILAGIFEPATIDIDLAILLTKAREHSVALVGPAAEELFDPVPEQDLFE

ALNETLTLWNSPPDWAGDERNVVLTLSRIWYSAVTGKIAPKDVAADWAMERLPAQYQPV

ILEARQAYLGQEEDRLASRADQLEEFVHYVKGEITKVVGK"

intron 2125..2269

/label=rbcS2 intron 1

intron 2624..2768

/label=rbcS2 intron 1

CDS 2943..2960

/codon_start=1

/label=GSGSGS-Linker

/translation="GSGSGS"

misc_feature 2963..2966

/label=fusion site

misc_feature join(2967..2985,3315..3316)

/label=GSGS-Linker

intron 2986..3314

/label=rbcS2 intron 2

CDS 3317..3340

/codon_start=1

/product="peptide that binds Strep-Tactin(R), an engineered

form of streptavidin"

/label=Strep-Tag II

/translation="WSHPQFEK"

misc_feature 3344..3347

/label=fusion site

3'UTR 3348..4070

/label=FDX1 3'UTR

/note="FDX1 3'UTR with mutated SapI site"

misc_feature 4075..4098

/label=link1eb

/note="/vntifkey=21"

misc_feature 4139..4163

/label=RB T-DNA repeat

/note="right border repeat from nopaline C58 T-DNA"

rep_origin 4250..4961

/label=oriV

/note="incP origin of replication"

promoter 4982..5086

/gene="bla"

/label=AmpR promoter

CDS 5087..5902

/codon_start=1

/gene="aph(3')-Ia"

/product="aminoglycoside phosphotransferase"

/label=KanR

/note="confers resistance to kanamycin in bacteria or G418

(Geneticin(R)) in eukaryotes"

/translation="MSHIQRETSCSRPRLNSNMDADLYGYKWARDNVGQSGATIYRLYG

KPDAPELFLKHGKGSVANDVTDEMVRLNWLTEFMPLPTIKHFIRTPDDAWLLTTAIPGK

TAFQVLEEYPDSGENIVDALAVFLRRLHSIPVCNCPFNSDRVFRLAQAQSRMNNGLVDA

SDFDDERNGWPVEQVWKEMHKLLPFSPDSVVTHGDFSLDNLIFDEGKLIGCIDVGRVGI

ADRYQDLAILWNCLGEFSPSLQKRLFQKYGIDNPDMNKLQFHLMLDEFF"

rep_origin 6073..6661

/direction=RIGHT

/label=ori

/note="high-copy-number ColE1/pMB1/pBR322/pUC origin of

replication"

CDS 6997..8145

/codon_start=1

/product="trans-acting replication protein that binds to

and activates oriV"

/label=trfA

/translation="MNRTFDRKAYRQELIDAGFSAEDAETIASRTVMRAPRETFQSVGS

MVQQATAKIERDSVQLAPPALPAPSAAVERSRRLEQEAAGLAKSMTIDTRGTMTTKKRK

TAGEDLAKQVSEAKQAALLKHTKQQIKEMQLSLFDIAPWPDTMRAMPNDTARSALFTTR

NKKIPREALQNKVIFHVNKDVKITYTGVELRADDDELVWQQVLEYAKRTPIGEPITFTF

YELCQDLGWSINGRYYTKAEECLSRLQATAMGFTSDRVGHLESVSLLHRFRVLDRGKKT

SRCQVLIDEEIVVLFAGDHYTKFIWEKYRKLSPTARRMFDYFSSHREPYPLKLETFRLM

CGSDSTRVKKWREQVGEACEELRGSGLVEHAWVNDDLVHCKR"

misc_feature 8291..8315

/label=LB T-DNA repeat

/note="left border repeat from nopaline C58 T-DNA"

ORIGIN

1 actggggttg aaaatattcg atcgattgcc tgaggcctgc cgaattcgga tccggaggct

61 gaggcttgac atgattggtg cgtatgtttg tatgaagcta caggactgat ttggcgggct

121 atgagggcgg gggaagctct ggaagggccg cgatggggcg cgcggcgtcc agaaggcgcc

181 atacggcccg ctggcggcac ccatccggta taaaagcccg cgaccccgaa cggtgacctc

241 cactttcagc gacaaacgag cacttataca tacgcgacta ttctgccgct atacataacc

301 actcagctag cttaagatcc catcaccggt ctggcacttt cttgcgctat gacacttcca

361 gcaaaaggta gggcgggctg cgagacggct tcccggcgct gcatgcaaca ccgatgatac

421 ttatgcttcg accccccgaa gctccttcgg ggctgcatgg gcgctccgat gccgctccag

481 ggcgagcgct gtttaaatag ccaggccccc gactgcaaag acattatagc gagctaccaa

541 agccatactt caaacaccta gatcactacc acttctacac aggccactcg agcttgtggt

601 gagtcgacga gcaagcccgg cggatcaggc agcgtgcttg cagatttgac ttgcaacgcc

661 cgcattgtgt cgacgaaggc ttttggctcc tctgtcgctg tctcaagcag catctaaccc

721 tgcgtcgccg tttccatttg cagatcgcac tccgctaagg gggcgcctct tcctcttcgt

781 ttcagtcaca acccgcaaaa tgggcagcgg cagcggcagc gtgagcaagg gcgaggagct

841 gttcaccggc gtggtgccca tcctggtgga gctggacggc gacgtgaacg gccacaagtt

901 cagcgtgagc ggcgagggcg agggcgacgc cacctacggc aagctgaccc tgaagctgat

961 ctgcaccacc ggcaagctgc ccgtgccctg gcccaccctg gtgaccaccc tgggctacgg

1021 tgagtcgacg agcaagcccg gcggatcagg cagcgtgctt gcagatttga cttgcaacgc

1081 ccgcattgtg tcgacgaagg cttttggctc ctctgtcgct gtctcaagca gcatctaacc

1141 ctgcgtcgcc gtttccattt gcaggcctgc agtgcttcgc ccgctacccc gaccacatga

1201 agcagcacga cttcttcaag agcgccatgc ccgagggcta cgtgcaggag cgcaccatct

1261 tcttcaagga cgacggtaac tacaagaccc gcgccgaggt gaagttcgag ggcgacaccc

1321 tggtgaaccg catcgagctg aagggcatcg acttcaagga ggacggcaac atcctgggcc

1381 acaagctgga gtacaactac aacagccaca acgtgtacat caccgccgac aagcagaaga

1441 acggcatcaa ggccaacttc aagatccgcc acaacatcga ggtgagtcga cgagcaagcc

1501 cggcggatca ggcagcgtgc ttgcagattt gacttgcaac gcccgcattg tgtcgacgaa

1561 ggcttttggc tcctctgtcg ctgtctcaag cagcatctaa ccctgcgtcg ccgtttccat

1621 ttgcaggacg gcggcgtgca gctggccgac cactaccagc agaacacccc catcggcgac

1681 ggccccgtgc tgctgcccga caaccactac ctgagctacc agagcaagct gagcaaggac

1741 cccaacgaga agcgcgacca catggtgctg ctggagttcg tgaccgccgc cggcatcacc

1801 ctgggcatgg acgagctgta caagggcagc ggcagcggca gcaggtcggg cagcggcagc

1861 ggcagccgcg aggccgtgat cgccgaggtg agcacccagc tgagcgaggt ggtgggcgtg

1921 atcgagcgcc acctggagcc caccctgctg gccgtgcacc tgtacggcag cgccgtggac

1981 ggcggcctga agccccacag cgacatcgac ctgctggtga ccgtgaccgt gcgcctggac

2041 gagacgaccc gccgcgccct gatcaacgac ctgctggaga cgagcgccag ccccggcgag

2101 agcgagatcc tgcgcgccgt ggaggtgagt cgacgagcaa gcccggcgga tcaggcagcg

2161 tgcttgcaga tttgacttgc aacgcccgca ttgtgtcgac gaaggctttt ggctcctctg

2221 tcgctgtctc aagcagcatc taaccctgcg tcgccgtttc catttgcagg tgaccatcgt

2281 ggtgcacgac gacatcatcc cctggcgcta ccccgccaag cgcgagctgc agttcggcga

2341 gtggcagcgc aacgacatcc tggccggcat cttcgagccc gccaccatcg acatcgacct

2401 ggccatcctg ctgaccaagg cccgcgagca cagcgtggcc ctggtgggcc ccgccgccga

2461 ggagctgttc gaccccgtgc ccgagcagga cctgttcgag gccctgaacg agacgctgac

2521 cctgtggaac agcccccccg actgggccgg cgacgagcgc aacgtggtgc tgaccctgag

2581 ccgcatctgg tacagcgccg tgaccggcaa gatcgccccc aaggtgagtc gacgagcaag

2641 cccggcggat caggcagcgt gcttgcagat ttgacttgca acgcccgcat tgtgtcgacg

2701 aaggcttttg gctcctctgt cgctgtctca agcagcatct aaccctgcgt cgccgtttcc

2761 atttgcagga cgtggccgcc gactgggcca tggagcgcct gcccgcccag taccagcccg

2821 tgatcctgga ggcccgccag gcctacctgg gccaggagga ggaccgcctg gccagccgcg

2881 ccgaccagct ggaggagttc gtgcactacg tgaagggcga gatcaccaag gtggtgggca

2941 agggcagcgg cagcggcagc gcttcgggca gcggcagcgg ctcaggtgag cttgcggggt

3001 tgcgagcaac actccagcaa cgaacagtgc ccaagtcagg aatctgcagt cagcctgggc

3061 tttcggcggc tttttcttgg gcaaacagct tgcactcatg ccagcgcggc ttgtccagcc

3121 tcacttgagc tttccagctg ctaccagccg ggctatacga cagcgacaga gccatagcgt

3181 ggaatcactt atttgggttg ccgaagtagc ggtcggagcg tgagttcttg gtcaagccgc

3241 cccttatccg gttcctgtcc gtgtctttgt ccctcgttca cccttcgcgg cacccttcat

3301 ccccttgctt gcaggttgga gccacccgca gttcgagaag taagcttgcg ctttcgccat

3361 ctgcgggggt cgtaggctag aactggggtt ggggatcggg ctgcttgcat agccaagcaa

3421 ttttccatct ggccagcaat ggcctagcac tatgagcggt tcaagtgtct cttgtgtgtt

3481 gtgtcgcatt gcatggccgt ggtgacctgc aattttctgt aaccggacat gcagaagctt

3541 cggttcgcgt cctttcctcg cttgctacgg gatcgggagt cggcagggct agaagtcttg

3601 ggtaacacgc gcaattcagc aatacaggcc agccagggca gcgaaggggg acttcagcaa

3661 ggacctctcg ggaatagtgg atgagctagg aggggtacag cagtagagat cgaggggtcc

3721 agctcacagt tctattacgt cgttcgtggg gacgaactgg gtcgaggcgc tacggactcg

3781 aaatgacggc agagggtggc aaggaagggg agcaccactg tgagcagttg cagcggcaca

3841 tacactacgt ctcttggcct taagcacagc cagcacactt gtacggggca acagtagccc

3901 cgaagcagcc tgatgcagtc acaccgtgcc gggccagtgt taacaaggaa gggcaggcac

3961 cagggcgagg gcaggcgcgg caaaactcgc cggttcctga cacggtgaca cgcaggtata

4021 cggtgacagc tcagctagtg ataccagctg ctccgcgttc tgaggagagg cgctgcaaga

4081 ggatgcacat gtgaccgagg gatacgatcg aatattatcc gtttaaacta tcagtgtttg

4141 acaggatata ttggcgggta aacctaagag aaaagagcgt ttattagaat aatcggatat

4201 ttaaaagggc gtgaaaaggt ttatccgttc gtccatttgt atgtgccagc cgcctttgcg

4261 acgctcaccg ggctggttgc cctcgccgct gggctggcgg ccgtctatgg ccctgcaaac

4321 gcgccagaaa cgccgtcgaa gccgtgtgcg agacaccgcg gccgccggcg ttgtggatac

4381 ctcgcggaaa acttggccct cactgacaga tgaggggcgg acgttgacac ttgaggggcc

4441 gactcacccg gcgcggcgtt gacagatgag gggcaggctc gatttcggcc ggcgacgtgg

4501 agctggccag cctcgcaaat cggcgaaaac gcctgatttt acgcgagttt cccacagatg

4561 atgtggacaa gcctggggat aagtgccctg cggtattgac acttgagggg cgcgactact

4621 gacagatgag gggcgcgatc cttgacactt gaggggcaga gtgctgacag atgaggggcg

4681 cacctattga catttgaggg gctgtccaca ggcagaaaat ccagcatttg caagggtttc

4741 cgcccgtttt tcggccaccg ctaacctgtc ttttaacctg cttttaaacc aatatttata

4801 aaccttgttt ttaaccaggg ctgcgccctg tgcgcgtgac cgcgcacgcc gaaggggggt

4861 gccccccctt ctcgaaccct cccggcccgc taacgcgggc ctcccatccc cccaggggct

4921 gcgcccctcg gccgcgaacg gcctcacccc aaaaatggca gcgctggcca attcccgagt

4981 gcgcggaacc cctatttgtt tatttttcta aatacattca aatatgtatc cgctcatgag

5041 acaataaccc tgataaatgc ttcaataata ttgaaaaagg aagagtatga gccatattca

5101 acgggaaacg tcttgctcta ggccgcgatt aaattccaac atggatgctg atttatatgg

5161 gtataaatgg gctcgcgata atgtcgggca atcaggtgcg acaatctatc gattgtatgg

5221 gaagcccgat gcgccagagt tgtttctgaa acatggcaaa ggtagcgttg ccaatgatgt

5281 tacagatgag atggtcagac taaactggct gacggaattt atgcctcttc cgaccatcaa

5341 gcattttatc cgtactcctg atgatgcatg gttactcacc actgcgatcc ccgggaaaac

5401 agcattccag gtattagaag aatatcctga ttcaggtgaa aatattgttg atgcgctggc

5461 agtgttcctg cgccggttgc attcgattcc tgtttgtaat tgtcctttta acagcgatcg

5521 cgtatttcgt ctcgctcagg cgcaatcacg aatgaataac ggtttggttg atgcgagtga

5581 ttttgatgac gagcgtaatg gctggcctgt tgaacaagtc tggaaagaaa tgcataaact

5641 tttgccattc tcaccggatt cagtcgtcac tcatggtgat ttctcacttg ataaccttat

5701 ttttgacgag gggaaattaa taggttgtat tgatgttgga cgagtcggaa tcgcagaccg

5761 ataccaggat cttgccatcc tatggaactg cctcggtgag ttttctcctt cattacagaa

5821 acggcttttt caaaaatatg gtattgataa tcctgatatg aataaattgc agtttcattt

5881 gatgctcgat gagtttttct aactgtcaga ccaagtttac tcatatatac tttagattga

5941 tttaaaactt catttttaat ttaaaaggat ctaggtgaag atcctttttg ataatctcat

6001 gaccaaaatc ccttaacgtg agttttcgtt ccactgagcg tcagaccccg tagaaaagat

6061 caaaggatct tcttgagatc ctttttttct gcgcgtaatc tgctgcttgc aaacaaaaaa

6121 accaccgcta ccagcggtgg tttgtttgcc ggatcaagag ctaccaactc tttttccgaa

6181 ggtaactggc ttcagcagag cgcagatacc aaatactgtc cttctagtgt agccgtagtt

6241 aggccaccac ttcaagaact ctgtagcacc gcctacatac ctcgctctgc taatcctgtt

6301 accagtggct gctgccagtg gcgataagtc gtgtcttacc gggttggact caagacgata

6361 gttaccggat aaggcgcagc ggtcgggctg aacggggggt tcgtgcacac agcccagctt

6421 ggagcgaacg acctacaccg aactgagata cctacagcgt gagctatgag aaagcgccac

6481 gcttcccgaa gggagaaagg cggacaggta tccggtaagc ggcagggtcg gaacaggaga

6541 gcgcacgagg gagcttccag ggggaaacgc ctggtatctt tatagtcctg tcgggtttcg

6601 ccacctctga cttgagcgtc gatttttgtg atgctcgtca ggggggcgga gcctatggaa

6661 aaacgccagc aacgcggcct ttttacggtt cctggcagat cctagatgtg gcgcaacgat

6721 gccggcgaca agcaggagcg caccgacttc ttccgcatca agtgttttgg ctctcaggcc

6781 gaggcccacg gcaagtattt gggcaagggg tcgctggtat tcgtgcaggg caagattcgg

6841 aataccaagt acgagaagga cggccagacg gtctacggga ccgacttcat tgccgataag

6901 gtggattatc tggacaccaa ggcaccaggc gggtcaaatc aggaataagg gcacattgcc

6961 ccggcgtgag tcggggcaat cccgcaagga gggtgaatga atcggacgtt tgaccggaag

7021 gcatacaggc aagaactgat cgacgcgggg ttttccgccg aggatgccga aaccatcgca

7081 agccgcaccg tcatgcgtgc gccccgcgaa accttccagt ccgtcggctc gatggtccag

7141 caagctacgg ccaagatcga gcgcgacagc gtgcaactgg ctccccctgc cctgcccgcg

7201 ccatcggccg ccgtggagcg ttcgcgtcgt cttgaacagg aggcggcagg tttggcgaag

7261 tcgatgacca tcgacacgcg aggaactatg acgaccaaga agcgaaaaac cgccggcgag

7321 gacctggcaa aacaggtcag cgaggccaag caggccgcgt tgctgaaaca cacgaagcag

7381 cagatcaagg aaatgcagct ttccttgttc gatattgcgc cgtggccgga cacgatgcga

7441 gcgatgccaa acgacacggc ccgctctgcc ctgttcacca cgcgcaacaa gaaaatcccg

7501 cgcgaggcgc tgcaaaacaa ggtcattttc cacgtcaaca aggacgtgaa gatcacctac

7561 accggcgtcg agctgcgggc cgacgatgac gaactggtgt ggcagcaggt gttggagtac

7621 gcgaagcgca cccctatcgg cgagccgatc accttcacgt tctacgagct ttgccaggac

7681 ctgggctggt cgatcaatgg ccggtattac acgaaggccg aggaatgcct gtcgcgccta

7741 caggcgacgg cgatgggctt cacgtccgac cgcgttgggc acctggaatc ggtgtcgctg

7801 ctgcaccgct tccgcgtcct ggaccgtggc aagaaaacgt cccgttgcca ggtcctgatc

7861 gacgaggaaa tcgtcgtgct gtttgctggc gaccactaca cgaaattcat atgggagaag

7921 taccgcaagc tgtcgccgac ggcccgacgg atgttcgact atttcagctc gcaccgggag

7981 ccgtacccgc tcaagctgga aaccttccgc ctcatgtgcg gatcggattc cacccgcgtg

8041 aagaagtggc gcgagcaggt cggcgaagcc tgcgaagagt tgcgaggcag cggcctggtg

8101 gaacacgcct gggtcaatga tgacctggtg cattgcaaac gctagggcct tgtggggtca

8161 gttccggctg ggggttcagc agcccctgct cggatctgtt ggaccggaca gtagtcatgg

8221 ttgatgggct gcctgtatcg agtggtgatt ttgtgccgag ctgccggtcg gggagctgtt

8281 ggctggctgg tggcaggata tattgtggtg taaacaaatt gacgcttaga caacttaata

8341 acacattgcg gacgttttta atgt

//

>Traditional expression of CnVs

LOCUS Exported 11253 bp ds-DNA circular SYN 02-AUG-2024

DEFINITION synthetic circular DNA

ACCESSION .

VERSION .

KEYWORDS .

SOURCE synthetic DNA construct

ORGANISM recombinant plasmid

REFERENCE 1 (bases 1 to 11253)

AUTHORS Thomas Baier

TITLE Direct Submission

JOURNAL Exported Friday, Aug 2, 2024 from SnapGene Viewer 4.3.11

https://www.snapgene.com

FEATURES Location/Qualifiers

source 1..11253

/organism="recombinant plasmid"

/mol_type="other DNA"

promoter 28..294

/label=P-HSP70A

promoter 306..498

/label=P-RBCS2

5'UTR 499..521

/label=5UTR CrRBCS2

CDS 523..1323

/codon_start=1

/label=AphVIII

/translation="MDDALRALRGRYPGCEWVVVEDGASGAGVYRLRGGGRELFVKVAA

LGAGVGLLGEAERLVWLAEVGIPVPRVVEGGGDERVAWLVTEAVPGRPASARWPREQRL

DVAVALAGLARSLHALDWERCPFDRSLAVTVPQAARAVAEGSVDLEDLDEERKGWSGER

LLAELERTRPADEDLAVCHGDLCPDNVLLDPRTCEVTGLIDVGRVGRADRHSDLALVLR

ELAHEEDPWFGPECSAAFLREYGRGWDGAVSEEKLAFYRLLDEFF"

terminator 1331..1564

/label=T-RBCS2

misc_feature 1577..1580

/label=fusion site

promoter 1581..1847

/label=HSP70Ap promoter

/label=HSP70Ap

promoter 1854..2045

/label=P-bTUB2

misc_feature 1940..1945

/label=inserted ATANTT motif

5'UTR join(2046..2121,2267..2321)

/label=5UTR bTUB2

misc_feature 2068..2073

/label=rebuild ATANTT from ATATT

intron 2122..2266

/label=RBCS2i

/label=RBCS2i(1)

misc_feature 2322..2325

/label=fusion site

CDS join(2326..2423,2569..2956,3102..3503,3649..4060,4206..4592,

4738..4814)

/codon_start=1

/product="AFN21429.1 terpene synthase Valencene synthase

[Callitropsis nootkatensis] Beekwilder 2014"

/label=AFN21429.1 terpene synthase Valencene synthase

/label=AFN21429.1 terpene synthase Valencene synthase

[Callitropsis nootkatensis] Beekwilder 2014__CDS

/translation="AEMFNGNSSNDGSSCMPVKDALRRTGNHHPNLWTDDFIQSLNSPY

SDSSYHKHREILIDEIRDMFSNGEGDEFGVLENIWFVDVVQRLGIDRHFQEEIKTALDY

IYKFWNHDSIFGDLNMVALGFRILRLNRYVASSDVFKKFKGEEGQFSGFESSDQDAKLE

MMLNLYKASELDFPDEDILKEARAFASMYLKHVIKEYGDIQESKNPLLMEIEYTFKYPW

RCRLPRLEAWNFIHIMRQQDCNISLANNLYKIPKIYMKKILELAILDFNILQSQHQHEM

KLISTWWKNSSAIQLDFFRHRHIESYFWWASPLFEPEFSTCRINCTKLSTKMFLLDDIY

DTYGTVEELKPFTTTLTRWDVSTVDNHPDYMKIAFNFSYEIYKEIASEAERKHGPFVYK

YLQSCWKSYIEAYMQEAEWIASNHIPGFDEYLMNGVKSSGMRILMIHALILMDTPLSDE

ILEQLDIPSSKSQALLSLITRLVDDVKDFEDEQAHGEMASSIECYMKDNHGSTREDALN

YLKIRIESCVQELNKELLEPSNMHGSFRNLYLNVGMRVIFFMLNDGDLFTHSNRKEIQD

AITKFFVEPIIP"

intron 2424..2568

/label=intron

intron 2957..3101

/label=intron

intron 3504..3648

/label=intron

intron 4061..4205

/label=intron

intron 4593..4737

/label=intron

CDS 4815..4832

/codon_start=1

/label=GSGSGS-Linker

/translation="GSGSGS"

misc_feature 4833..4836

/label=fusion site

CDS 4839..4856

/label=GSGSGS-Linker

gene join(4857..5055,5201..5517,5663..5860)

/label=mVenus

misc_feature 4969..4991

/label=sgRNA-target site

misc_feature 5028..5050

/label=sgRNA-target site

intron 5056..5200

/label=rbcS2 intron 1

misc_feature 5202..5224

/label=sgRNA-target-exclusive

intron 5518..5662

/label=rbcS2 intron 1

CDS 5861..5878

/codon_start=1

/label=GSGSGS-Linker

/translation="GSGSGS"

misc_feature 5881..5884

/label=fusion site

misc_feature join(5885..5903,6233..6234)

/label=GSGS-Linker

intron 5904..6232

/label=rbcS2 intron 2

CDS 6235..6258

/codon_start=1

/product="peptide that binds Strep-Tactin(R), an engineered

form of streptavidin"

/label=Strep-Tag II

/translation="WSHPQFEK"

misc_feature 6262..6265

/label=fusion site

3'UTR 6266..6988

/label=FDX1 3'UTR

misc_feature 7027..7051

/label=RB T-DNA repeat

/note="right border repeat from nopaline C58 T-DNA"

rep_origin 7139..7849

/label=oriV

/note="incP origin of replication"

promoter 7870..7974

/gene="bla"

/label=AmpR promoter

CDS 7975..8790

/codon_start=1

/gene="aph(3')-Ia"

/product="aminoglycoside phosphotransferase"

/label=KanR

/note="confers resistance to kanamycin in bacteria or G418

(Geneticin(R)) in eukaryotes"

/translation="MSHIQRETSCSRPRLNSNMDADLYGYKWARDNVGQSGATIYRLYG

KPDAPELFLKHGKGSVANDVTDEMVRLNWLTEFMPLPTIKHFIRTPDDAWLLTTAIPGK

TAFQVLEEYPDSGENIVDALAVFLRRLHSIPVCNCPFNSDRVFRLAQAQSRMNNGLVDA

SDFDDERNGWPVEQVWKEMHKLLPFSPDSVVTHGDFSLDNLIFDEGKLIGCIDVGRVGI

ADRYQDLAILWNCLGEFSPSLQKRLFQKYGIDNPDMNKLQFHLMLDEFF"

rep_origin 8961..9549

/direction=RIGHT

/label=ori

/note="high-copy-number ColE1/pMB1/pBR322/pUC origin of

replication"

CDS 9885..11033

/codon_start=1

/product="trans-acting replication protein that binds to

and activates oriV"

/label=trfA

/translation="MNRTFDRKAYRQELIDAGFSAEDAETIASRTVMRAPRETFQSVGS

MVQQATAKIERDSVQLAPPALPAPSAAVERSRRLEQEAAGLAKSMTIDTRGTMTTKKRK

TAGEDLAKQVSEAKQAALLKHTKQQIKEMQLSLFDIAPWPDTMRAMPNDTARSALFTTR

NKKIPREALQNKVIFHVNKDVKITYTGVELRADDDELVWQQVLEYAKRTPIGEPITFTF

YELCQDLGWSINGRYYTKAEECLSRLQATAMGFTSDRVGHLESVSLLHRFRVLDRGKKT

SRCQVLIDEEIVVLFAGDHYTKFIWEKYRKLSPTARRMFDYFSSHREPYPLKLETFRLM

CGSDSTRVKKWREQVGEACEELRGSGLVEHAWVNDDLVHCKR"

misc_feature 11179..11203

/label=LB T-DNA repeat

/note="left border repeat from nopaline C58 T-DNA"

ORIGIN

1 ctggggttga aaatattcga tcgtgccgct gaggcttgac atgattggtg cgtatgtttg

61 tatgaagcta caggactgat ttggcgggct atgagggcgg gggaagctct ggaagggccg

121 cgatggggcg cgcggcgtcc agaaggcgcc atacggcccg ctggcggcac ccatccggta

181 taaaagcccg cgaccccgaa cggtgacctc cactttcagc gacaaacgag cacttataca

241 tacgcgacta ttctgccgct atacataacc actcagctag cttaagatcc catcaagctt

301 gcatgccggg cgcgccagaa ggagcgcagc caaaccagga tgatgtttga tggggtattt

361 gagcacttgc aacccttatc cggaagcccc ctggcccaca aaggctaggc gccaatgcaa

421 gcagttcgca tgcagcccct ggagcggtgc cctcctgata aaccggccag ggggcctatg

481 ttctttactt ttttacaaga gaagtcactc aacatcttaa aaatggacga tgcgttgcgt

541 gcactgcggg gtcggtatcc cggttgtgag tgggttgttg tggaggatgg ggcctcgggg

601 gctggtgttt atcggcttcg gggtggtggg cgggagttgt ttgtcaaggt ggcagctctg

661 ggggccgggg tgggcttgtt gggtgaggct gagcggctgg tgtggttggc ggaggtgggg

721 attcccgtac ctcgtgttgt ggagggtggt ggggacgaga gggtcgcctg gttggtcacc

781 gaagcggttc cggggcgtcc ggccagtgcg cggtggccgc gggagcagcg gctggacgtg

841 gcggtggcgc tcgcggggct cgctcgttcg ctgcacgcgc tggactggga gcggtgtccg

901 ttcgatcgca gtctcgcggt gacggtgccg caggcggccc gtgctgtcgc tgaagggagc

961 gtcgacttgg aggatctgga cgaggagcgg aaggggtggt cgggggagcg gcttctcgcc

1021 gagctggagc ggactcggcc tgcggacgag gatctggcgg tttgccacgg tgacctgtgc

1081 ccggacaacg tgctgctcga ccctcgtacc tgcgaggtga ccgggctgat cgacgtgggg

1141 cgggtcggcc gtgcggaccg gcactccgat ctcgcgctgg tgctgcgcga gctggcccac

1201 gaggaggacc cgtggttcgg gccggagtgt tccgcggcgt tcctgcggga gtacgggcgc

1261 gggtgggatg gggcggtatc ggaggaaaag ctggcgtttt accggctgtt ggacgagttc

1321 ttctgagctt ccgctccgtg taaatggagg cgctcgttga tctgagcctt gccccctgac

1381 gaacggcggt ggatggaaga tactgctctc aagtgctgaa gcggtagctt agctccccgt

1441 ttcgtgctga tcagtctttt tcaacacgta aaaagcggag gagttttgca attttgttgg

1501 ttgtaacgat cctccgttga ttttggcctc tttctccatg ggcgggctgg gcgtatttga

1561 agcggaattc aagcttggag gctgaggctt gacatgattg gtgcgtatgt ttgtatgaag

1621 ctacaggact gatttggcgg gctatgaggg cgggggaagc tctggaaggg ccgcgatggg

1681 gcgcgcggcg tccagaaggc gccatacggc ccgctggcgg cacccatccg gtataaaagc

1741 ccgcgacccc gaacggtgac ctccactttc agcgacaaac gagcacttat acatacgcga

1801 ctattctgcc gctatacata accactcagc tagcttaaga tcccatcacc ggtctggcac

1861 tttcttgcgc tatgacactt ccagcaaaag gtagggcggg ctgcgagacg gcttcccggc

1921 gctgcatgca acaccgatga tacttatgct tcgacccccc gaagctcctt cggggctgca

1981 tgggcgctcc gatgccgctc cagggcgagc gctgtttaaa tagccaggcc cccgactgca

2041 aagacattat agcgagctac caaagccata cttcaaacac ctagatcact accacttcta

2101 cacaggccac tcgagcttgt ggtgagtcga cgagcaagcc cggcggatca ggcagcgtgc

2161 ttgcagattt gacttgcaac gcccgcattg tgtcgacgaa ggcttttggc tcctctgtcg

2221 ctgtctcaag cagcatctaa ccctgcgtcg ccgtttccat ttgcagatcg cactccgcta

2281 agggggcgcc tcttcctctt cgtttcagtc acaacccgca aaatggccga gatgttcaac

2341 ggcaacagca gcaacgacgg cagcagctgc atgcccgtga aggacgccct gcgccgcacc

2401 ggcaaccacc accccaacct gtggtgagtc gacgagcaag cccggcggat caggcagcgt

2461 gcttgcagat ttgacttgca acgcccgcat tgtgtcgacg aaggcttttg gctcctctgt

2521 cgctgtctca agcagcatct aaccctgcgt cgccgtttcc atttgcagga ccgacgactt

2581 catccagagc ctgaacagcc cctacagcga cagcagctac cacaagcacc gcgagatcct

2641 gatcgacgag atccgcgaca tgttcagcaa cggcgagggc gacgagttcg gcgtgctgga

2701 gaacatctgg ttcgtggacg tggtgcagcg cctgggcatc gaccgccact tccaggagga

2761 gatcaagacc gccctggact acatctacaa gttctggaac cacgacagca tcttcggcga

2821 cctgaacatg gtggccctgg gcttccgcat cctgcgcctg aaccgctacg tggccagcag

2881 cgacgtgttc aagaagttca agggcgagga gggccagttc agcggcttcg agagcagcga

2941 ccaggacgcc aagctggtga gtcgacgagc aagcccggcg gatcaggcag cgtgcttgca

3001 gatttgactt gcaacgcccg cattgtgtcg acgaaggctt ttggctcctc tgtcgctgtc

3061 tcaagcagca tctaaccctg cgtcgccgtt tccatttgca ggagatgatg ctgaacctgt

3121 acaaggccag cgagctggac ttccccgacg aggacatcct gaaggaggcc cgcgccttcg

3181 ccagcatgta cctgaagcac gtgatcaagg agtacggcga catccaggag agcaagaacc

3241 ccctgctgat ggagatcgag tacaccttca agtacccctg gcgctgccgc ctgccccgcc

3301 tggaggcctg gaacttcatc cacatcatgc gccagcagga ctgcaacatc agcctggcca

3361 acaacctgta caagatcccc aagatttaca tgaagaagat cctggagctg gccatcctgg

3421 acttcaacat cctgcagagc cagcaccagc acgagatgaa gctgatcagc acctggtgga

3481 agaacagcag cgccatccag ctggtgagtc gacgagcaag cccggcggat caggcagcgt

3541 gcttgcagat ttgacttgca acgcccgcat tgtgtcgacg aaggcttttg gctcctctgt

3601 cgctgtctca agcagcatct aaccctgcgt cgccgtttcc atttgcagga cttcttccgc

3661 caccgccaca tcgagagcta cttctggtgg gccagccccc tgttcgagcc cgagttcagc

3721 acctgccgca tcaactgcac caagctgagc accaagatgt tcctgctgga cgacatctac

3781 gacacctacg gcaccgtgga ggagctgaag cccttcacca ccaccctgac ccgctgggac

3841 gtgagcaccg tggacaacca ccccgactac atgaagatcg ccttcaactt cagctacgag

3901 atttacaagg agatcgccag cgaggccgag cgcaagcacg gccccttcgt gtacaagtac

3961 ctgcagagct gctggaagag ctacatcgag gcctacatgc aggaggccga gtggatcgcc

4021 agcaaccaca tccccggctt cgacgagtac ctgatgaacg gtgagtcgac gagcaagccc

4081 ggcggatcag gcagcgtgct tgcagatttg acttgcaacg cccgcattgt gtcgacgaag

4141 gcttttggct cctctgtcgc tgtctcaagc agcatctaac cctgcgtcgc cgtttccatt

4201 tgcaggcgtg aagagcagcg gcatgcgcat cctgatgatc cacgccctga tcctgatgga

4261 cacccccctg agcgacgaga tcctggagca gctggacatc cccagcagca agagccaggc

4321 cctgctgagc ctgatcaccc gcctggtgga cgacgtgaag gacttcgagg acgagcaggc

4381 ccacggcgag atggccagca gcatcgagtg ctacatgaag gacaaccacg gcagcacccg

4441 cgaggacgcc ctgaactacc tgaagatccg catcgagagc tgcgtgcagg agctgaacaa

4501 ggagctgctg gagcccagca acatgcacgg cagcttccgc aacctgtacc tgaacgtggg

4561 catgcgcgtg atcttcttca tgctgaacga cggtgagtcg acgagcaagc ccggcggatc

4621 aggcagcgtg cttgcagatt tgacttgcaa cgcccgcatt gtgtcgacga aggcttttgg

4681 ctcctctgtc gctgtctcaa gcagcatcta accctgcgtc gccgtttcca tttgcaggcg

4741 acctgttcac ccacagcaac cgcaaggaga tccaggacgc catcaccaag ttcttcgtgg

4801 agcccatcat ccccggcagc ggcagcggca gcaggtcggg cagcggcagc ggcagcgtga

4861 gcaagggcga ggagctgttc accggcgtgg tgcccatcct ggtggagctg gacggcgacg

4921 tgaacggcca caagttcagc gtgagcggcg agggcgaggg cgacgccacc tacggcaagc

4981 tgaccctgaa gctgatctgc accaccggca agctgcccgt gccctggccc accctggtga

5041 ccaccctggg ctacggtgag tcgacgagca agcccggcgg atcaggcagc gtgcttgcag

5101 atttgacttg caacgcccgc attgtgtcga cgaaggcttt tggctcctct gtcgctgtct

5161 caagcagcat ctaaccctgc gtcgccgttt ccatttgcag gcctgcagtg cttcgcccgc

5221 taccccgacc acatgaagca gcacgacttc ttcaagagcg ccatgcccga gggctacgtg

5281 caggagcgca ccatcttctt caaggacgac ggtaactaca agacccgcgc cgaggtgaag

5341 ttcgagggcg acaccctggt gaaccgcatc gagctgaagg gcatcgactt caaggaggac

5401 ggcaacatcc tgggccacaa gctggagtac aactacaaca gccacaacgt gtacatcacc

5461 gccgacaagc agaagaacgg catcaaggcc aacttcaaga tccgccacaa catcgaggtg

5521 agtcgacgag caagcccggc ggatcaggca gcgtgcttgc agatttgact tgcaacgccc

5581 gcattgtgtc gacgaaggct tttggctcct ctgtcgctgt ctcaagcagc atctaaccct

5641 gcgtcgccgt ttccatttgc aggacggcgg cgtgcagctg gccgaccact accagcagaa

5701 cacccccatc ggcgacggcc ccgtgctgct gcccgacaac cactacctga gctaccagag

5761 caagctgagc aaggacccca acgagaagcg cgaccacatg gtgctgctgg agttcgtgac

5821 cgccgccggc atcaccctgg gcatggacga gctgtacaag ggcagcggca gcggcagcgc

5881 ttcgggcagc ggcagcggct caggtgagct tgcggggttg cgagcaacac tccagcaacg

5941 aacagtgccc aagtcaggaa tctgcagtca gcctgggctt tcggcggctt tttcttgggc

6001 aaacagcttg cactcatgcc agcgcggctt gtccagcctc acttgagctt tccagctgct

6061 accagccggg ctatacgaca gcgacagagc catagcgtgg aatcacttat ttgggttgcc

6121 gaagtagcgg tcggagcgtg agttcttggt caagccgccc cttatccggt tcctgtccgt

6181 gtctttgtcc ctcgttcacc cttcgcggca cccttcatcc ccttgcttgc aggttggagc

6241 cacccgcagt tcgagaagta agcttgcgct ttcgccatct gcgggggtcg taggctagaa

6301 ctggggttgg ggatcgggct gcttgcatag ccaagcaatt ttccatctgg ccagcaatgg

6361 cctagcacta tgagcggttc aagtgtctct tgtgtgttgt gtcgcattgc atggccgtgg

6421 tgacctgcaa ttttctgtaa ccggacatgc agaagcttcg gttcgcgtcc tttcctcgct

6481 tgctacggga tcgggagtcg gcagggctag aagtcttggg taacacgcgc aattcagcaa

6541 tacaggccag ccagggcagc gaagggggac ttcagcaagg acctctcggg aatagtggaa

6601 gagctaggag gggtacagca gtagagatcg aggggtccag ctcacagttc tattacgtcg

6661 ttcgtgggga cgaactgggt cgaggcgcta cggactcgaa atgacggcag agggtggcaa

6721 ggaaggggag caccactgtg agcagttgca gcggcacata cactacgtct cttggcctta

6781 agcacagcca gcacacttgt acggggcaac agtagccccg aagcagcctg atgcagtcac

6841 accgtgccgg gccagtgtta acaaggaagg gcaggcacca gggcgagggc aggcgcggca

6901 aaactcgccg gttcctgaca cggtgacacg caggtatacg gtgacagctc agctagtgat

6961 accagctgct ccgcgttctg aggagaggcg ctcgatcgaa tattatccgt ttaaactatc

7021 agtgtttgac aggatatatt ggcgggtaaa cctaagagaa aagagcgttt attagaataa

7081 tcggatattt aaaagggcgt gaaaaggttt atccgttcgt ccatttgtat gtgccagccg

7141 cctttgcgac gctcaccggg ctggttgccc tcgccgctgg gctggcggcc gtctatggcc

7201 ctgcaaacgc gccagaaacg ccgtcgaagc cgtgtgcgag acaccgcggc cgccggcgtt

7261 gtggatacct cgcggaaaac ttggccctca ctgacagatg aggggcggac gttgacactt

7321 gaggggccga ctcacccggc gcggcgttga cagatgaggg gcaggctcga tttcggccgg

7381 cgacgtggag ctggccagcc tcgcaaatcg gcgaaaacgc ctgattttac gcgagtttcc

7441 cacagatgat gtggacaagc ctggggataa gtgccctgcg gtattgacac ttgaggggcg

7501 cgactactga cagatgaggg gcgcgatcct tgacacttga ggggcagagt gctgacagat

7561 gaggggcgca cctattgaca tttgaggggc tgtccacagg cagaaaatcc agcatttgca

7621 agggtttccg cccgtttttc ggccaccgct aacctgtctt ttaacctgct tttaaaccaa

7681 tatttataaa ccttgttttt aaccagggct gcgccctgtg cgcgtgaccg cgcacgccga

7741 aggggggtgc ccccccttct cgaaccctcc cggcccgcta acgcgggcct cccatccccc

7801 caggggctgc gcccctcggc cgcgaacggc ctcaccccaa aaatggcagc gctggccaat

7861 tcccgagtgc gcggaacccc tatttgttta tttttctaaa tacattcaaa tatgtatccg

7921 ctcatgagac aataaccctg ataaatgctt caataatatt gaaaaaggaa gagtatgagc

7981 catattcaac gggaaacgtc ttgctctagg ccgcgattaa attccaacat ggatgctgat

8041 ttatatgggt ataaatgggc tcgcgataat gtcgggcaat caggtgcgac aatctatcga

8101 ttgtatggga agcccgatgc gccagagttg tttctgaaac atggcaaagg tagcgttgcc

8161 aatgatgtta cagatgagat ggtcagacta aactggctga cggaatttat gcctcttccg

8221 accatcaagc attttatccg tactcctgat gatgcatggt tactcaccac tgcgatcccc

8281 gggaaaacag cattccaggt attagaagaa tatcctgatt caggtgaaaa tattgttgat

8341 gcgctggcag tgttcctgcg ccggttgcat tcgattcctg tttgtaattg tccttttaac

8401 agcgatcgcg tatttcgtct cgctcaggcg caatcacgaa tgaataacgg tttggttgat

8461 gcgagtgatt ttgatgacga gcgtaatggc tggcctgttg aacaagtctg gaaagaaatg

8521 cataaacttt tgccattctc accggattca gtcgtcactc atggtgattt ctcacttgat

8581 aaccttattt ttgacgaggg gaaattaata ggttgtattg atgttggacg agtcggaatc

8641 gcagaccgat accaggatct tgccatccta tggaactgcc tcggtgagtt ttctccttca

8701 ttacagaaac ggctttttca aaaatatggt attgataatc ctgatatgaa taaattgcag

8761 tttcatttga tgctcgatga gtttttctaa ctgtcagacc aagtttactc atatatactt

8821 tagattgatt taaaacttca tttttaattt aaaaggatct aggtgaagat cctttttgat

8881 aatctcatga ccaaaatccc ttaacgtgag ttttcgttcc actgagcgtc agaccccgta

8941 gaaaagatca aaggatcttc ttgagatcct ttttttctgc gcgtaatctg ctgcttgcaa

9001 acaaaaaaac caccgctacc agcggtggtt tgtttgccgg atcaagagct accaactctt

9061 tttccgaagg taactggctt cagcagagcg cagataccaa atactgtcct tctagtgtag

9121 ccgtagttag gccaccactt caagaactct gtagcaccgc ctacatacct cgctctgcta

9181 atcctgttac cagtggctgc tgccagtggc gataagtcgt gtcttaccgg gttggactca

9241 agacgatagt taccggataa ggcgcagcgg tcgggctgaa cggggggttc gtgcacacag

9301 cccagcttgg agcgaacgac ctacaccgaa ctgagatacc tacagcgtga gctatgagaa

9361 agcgccacgc ttcccgaagg gagaaaggcg gacaggtatc cggtaagcgg cagggtcgga

9421 acaggagagc gcacgaggga gcttccaggg ggaaacgcct ggtatcttta tagtcctgtc

9481 gggtttcgcc acctctgact tgagcgtcga tttttgtgat gctcgtcagg ggggcggagc

9541 ctatggaaaa acgccagcaa cgcggccttt ttacggttcc tggcagatcc tagatgtggc

9601 gcaacgatgc cggcgacaag caggagcgca ccgacttctt ccgcatcaag tgttttggct

9661 ctcaggccga ggcccacggc aagtatttgg gcaaggggtc gctggtattc gtgcagggca

9721 agattcggaa taccaagtac gagaaggacg gccagacggt ctacgggacc gacttcattg

9781 ccgataaggt ggattatctg gacaccaagg caccaggcgg gtcaaatcag gaataagggc

9841 acattgcccc ggcgtgagtc ggggcaatcc cgcaaggagg gtgaatgaat cggacgtttg

9901 accggaaggc atacaggcaa gaactgatcg acgcggggtt ttccgccgag gatgccgaaa

9961 ccatcgcaag ccgcaccgtc atgcgtgcgc cccgcgaaac cttccagtcc gtcggctcga

10021 tggtccagca agctacggcc aagatcgagc gcgacagcgt gcaactggct ccccctgccc

10081 tgcccgcgcc atcggccgcc gtggagcgtt cgcgtcgtct tgaacaggag gcggcaggtt

10141 tggcgaagtc gatgaccatc gacacgcgag gaactatgac gaccaagaag cgaaaaaccg

10201 ccggcgagga cctggcaaaa caggtcagcg aggccaagca ggccgcgttg ctgaaacaca

10261 cgaagcagca gatcaaggaa atgcagcttt ccttgttcga tattgcgccg tggccggaca

10321 cgatgcgagc gatgccaaac gacacggccc gctctgccct gttcaccacg cgcaacaaga

10381 aaatcccgcg cgaggcgctg caaaacaagg tcattttcca cgtcaacaag gacgtgaaga

10441 tcacctacac cggcgtcgag ctgcgggccg acgatgacga actggtgtgg cagcaggtgt

10501 tggagtacgc gaagcgcacc cctatcggcg agccgatcac cttcacgttc tacgagcttt

10561 gccaggacct gggctggtcg atcaatggcc ggtattacac gaaggccgag gaatgcctgt

10621 cgcgcctaca ggcgacggcg atgggcttca cgtccgaccg cgttgggcac ctggaatcgg

10681 tgtcgctgct gcaccgcttc cgcgtcctgg accgtggcaa gaaaacgtcc cgttgccagg

10741 tcctgatcga cgaggaaatc gtcgtgctgt ttgctggcga ccactacacg aaattcatat

10801 gggagaagta ccgcaagctg tcgccgacgg cccgacggat gttcgactat ttcagctcgc

10861 accgggagcc gtacccgctc aagctggaaa ccttccgcct catgtgcgga tcggattcca

10921 cccgcgtgaa gaagtggcgc gagcaggtcg gcgaagcctg cgaagagttg cgaggcagcg

10981 gcctggtgga acacgcctgg gtcaatgatg acctggtgca ttgcaaacgc tagggccttg

11041 tggggtcagt tccggctggg ggttcagcag cccctgctcg gatctgttgg accggacagt

11101 agtcatggtt gatgggctgc ctgtatcgag tggtgatttt gtgccgagct gccggtcggg

11161 gagctgttgg ctggctggtg gcaggatata ttgtggtgta aacaaattga cgcttagaca

11221 acttaataac acattgcgga cgtttttaat gta

//

>Targeted integration of mVenus on LHCBM1 locus

LOCUS Exported 6722 bp ds-DNA circular SYN 02-AUG-2024

DEFINITION synthetic circular DNA

ACCESSION .

VERSION .

KEYWORDS .

SOURCE synthetic DNA construct

ORGANISM recombinant plasmid

REFERENCE 1 (bases 1 to 6722)

AUTHORS Thomas Baier

TITLE Direct Submission

JOURNAL Exported Friday, Aug 2, 2024 from SnapGene Viewer 4.3.11

https://www.snapgene.com

FEATURES Location/Qualifiers

source 1..6722

/organism="recombinant plasmid"

/mol_type="other DNA"

misc_feature 50..56

/label=Insertion Event

misc_feature 64..110

/label=HA EcoRV

misc_feature 105..107

/label=PAM

misc_feature 108..127

/label=sgRNA1 rev

CDS 111..177

/codon_start=1

/label=LHCBM1

/translation="SDHLANPGTNNAFAYATKFTPQ"

CDS 178..195

/codon_start=1

/label=GSGSGS-Linker

/translation="GSGSGS"

CDS 196..313

/codon_start=1

/product="unnamed_input_seq"

/label=ext2A

/label=unnamed_input_seq__CDS

/translation="LLAIHPTEARHKQKIVAPVKQTLNFDLLKLAGDVESNPG"

CDS 316..333

/codon_start=1

/label=GSGSGS-Linker

/translation="GSGSGS"

CDS join(334..532,678..994,1140..1337)

/codon_start=1

/label=mVenus

/translation="VSKGEELFTGVVPILVELDGDVNGHKFSVSGEGEGDATYGKLTLK

LICTTGKLPVPWPTLVTTLGYGLQCFARYPDHMKQHDFFKSAMPEGYVQERTIFFKDDG

NYKTRAEVKFEGDTLVNRIELKGIDFKEDGNILGHKLEYNYNSHNVYITADKQKNGIKA

NFKIRHNIEDGGVQLADHYQQNTPIGDGPVLLPDNHYLSYQSKLSKDPNEKRDHMVLLE

FVTAAGITLGMDELYK"

intron 533..677

/label=rbcS2 intron 1

intron 995..1139

/label=rbcS2 intron 1

CDS 1338..1355

/codon_start=1

/label=GSGSGS-Linker

/translation="GSGSGS"

CDS join(1356..1613,1759..2112,2258..2431)

/codon_start=1

/label=AadA Spectinomycin resistance

/translation="REAVIAEVSTQLSEVVGVIERHLEPTLLAVHLYGSAVDGGLKPHS

DIDLLVTVTVRLDETTRRALINDLLETSASPGESEILRAVEVTIVVHDDIIPWRYPAKR

ELQFGEWQRNDILAGIFEPATIDIDLAILLTKAREHSVALVGPAAEELFDPVPEQDLFE

ALNETLTLWNSPPDWAGDERNVVLTLSRIWYSAVTGKIAPKDVAADWAMERLPAQYQPV

ILEARQAYLGQEEDRLASRADQLEEFVHYVKGEITKVVGK"

intron 1614..1758

/label=rbcS2 intron 1

intron 2113..2257

/label=rbcS2 intron 1

3'UTR 2435..2472

misc_feature 2435..2472

/label=HA2 EcoRV

misc_feature 2516..2540

/label=RB T-DNA repeat

/note="right border repeat from nopaline C58 T-DNA"

rep_origin 2627..3338

/label=oriV

/note="incP origin of replication"

promoter 3359..3463

/gene="bla"

/label=AmpR promoter

CDS 3464..4279

/codon_start=1

/gene="aph(3')-Ia"

/product="aminoglycoside phosphotransferase"

/label=KanR

/note="confers resistance to kanamycin in bacteria or G418

(Geneticin(R)) in eukaryotes"

/translation="MSHIQRETSCSRPRLNSNMDADLYGYKWARDNVGQSGATIYRLYG

KPDAPELFLKHGKGSVANDVTDEMVRLNWLTEFMPLPTIKHFIRTPDDAWLLTTAIPGK

TAFQVLEEYPDSGENIVDALAVFLRRLHSIPVCNCPFNSDRVFRLAQAQSRMNNGLVDA

SDFDDERNGWPVEQVWKEMHKLLPFSPDSVVTHGDFSLDNLIFDEGKLIGCIDVGRVGI

ADRYQDLAILWNCLGEFSPSLQKRLFQKYGIDNPDMNKLQFHLMLDEFF"

rep_origin 4450..5038

/direction=RIGHT

/label=ori

/note="high-copy-number ColE1/pMB1/pBR322/pUC origin of

replication"

CDS 5374..6522

/codon_start=1

/product="trans-acting replication protein that binds to

and activates oriV"

/label=trfA

/translation="MNRTFDRKAYRQELIDAGFSAEDAETIASRTVMRAPRETFQSVGS

MVQQATAKIERDSVQLAPPALPAPSAAVERSRRLEQEAAGLAKSMTIDTRGTMTTKKRK

TAGEDLAKQVSEAKQAALLKHTKQQIKEMQLSLFDIAPWPDTMRAMPNDTARSALFTTR

NKKIPREALQNKVIFHVNKDVKITYTGVELRADDDELVWQQVLEYAKRTPIGEPITFTF

YELCQDLGWSINGRYYTKAEECLSRLQATAMGFTSDRVGHLESVSLLHRFRVLDRGKKT

SRCQVLIDEEIVVLFAGDHYTKFIWEKYRKLSPTARRMFDYFSSHREPYPLKLETFRLM

CGSDSTRVKKWREQVGEACEELRGSGLVEHAWVNDDLVHCKR"

misc_feature 6668..6692

/label=LB T-DNA repeat

/note="left border repeat from nopaline C58 T-DNA"

ORIGIN

1 ttgcggacgt ttttaatgta ctggggttga aaatattcga tcgattgcct gaggcctgcc

61 gatatcttag tttacccggt gatgttatgc tcctcgtgcc cgctccacag gtccgaccac

121 ctggccaacc ccggcaccaa caacgccttc gcctacgcca ccaagttcac cccccagggc

181 agcggcagcg gcagcctgct ggccatccac cccaccgagg cccgccacaa gcagaagatc

241 gtggcccccg tgaagcagac cctgaacttc gacctgctga agctggccgg cgacgtggag

301 agcaaccccg gccccggcag cggcagcggc agcgtgagca agggcgagga gctgttcacc

361 ggcgtggtgc ccatcctggt ggagctggac ggcgacgtga acggccacaa gttcagcgtg

421 agcggcgagg gcgagggcga cgccacctac ggcaagctga ccctgaagct gatctgcacc

481 accggcaagc tgcccgtgcc ctggcccacc ctggtgacca ccctgggcta cggtgagtcg

541 acgagcaagc ccggcggatc aggcagcgtg cttgcagatt tgacttgcaa cgcccgcatt

601 gtgtcgacga aggcttttgg ctcctctgtc gctgtctcaa gcagcatcta accctgcgtc

661 gccgtttcca tttgcaggcc tgcagtgctt cgcccgctac cccgaccaca tgaagcagca

721 cgacttcttc aagagcgcca tgcccgaggg ctacgtgcag gagcgcacca tcttcttcaa

781 ggacgacggt aactacaaga cccgcgccga ggtgaagttc gagggcgaca ccctggtgaa

841 ccgcatcgag ctgaagggca tcgacttcaa ggaggacggc aacatcctgg gccacaagct

901 ggagtacaac tacaacagcc acaacgtgta catcaccgcc gacaagcaga agaacggcat

961 caaggccaac ttcaagatcc gccacaacat cgaggtgagt cgacgagcaa gcccggcgga

1021 tcaggcagcg tgcttgcaga tttgacttgc aacgcccgca ttgtgtcgac gaaggctttt

1081 ggctcctctg tcgctgtctc aagcagcatc taaccctgcg tcgccgtttc catttgcagg

1141 acggcggcgt gcagctggcc gaccactacc agcagaacac ccccatcggc gacggccccg

1201 tgctgctgcc cgacaaccac tacctgagct accagagcaa gctgagcaag gaccccaacg

1261 agaagcgcga ccacatggtg ctgctggagt tcgtgaccgc cgccggcatc accctgggca

1321 tggacgagct gtacaagggc agcggcagcg gcagccgcga ggccgtgatc gccgaggtga

1381 gcacccagct gagcgaggtg gtgggcgtga tcgagcgcca cctggagccc accctgctgg

1441 ccgtgcacct gtacggcagc gccgtggacg gcggcctgaa gccccacagc gacatcgacc

1501 tgctggtgac cgtgaccgtg cgcctggacg agacgacccg ccgcgccctg atcaacgacc

1561 tgctggagac gagcgccagc cccggcgaga gcgagatcct gcgcgccgtg gaggtgagtc

1621 gacgagcaag cccggcggat caggcagcgt gcttgcagat ttgacttgca acgcccgcat

1681 tgtgtcgacg aaggcttttg gctcctctgt cgctgtctca agcagcatct aaccctgcgt

1741 cgccgtttcc atttgcaggt gaccatcgtg gtgcacgacg acatcatccc ctggcgctac

1801 cccgccaagc gcgagctgca gttcggcgag tggcagcgca acgacatcct ggccggcatc

1861 ttcgagcccg ccaccatcga catcgacctg gccatcctgc tgaccaaggc ccgcgagcac

1921 agcgtggccc tggtgggccc cgccgccgag gagctgttcg accccgtgcc cgagcaggac

1981 ctgttcgagg ccctgaacga gacgctgacc ctgtggaaca gcccccccga ctgggccggc

2041 gacgagcgca acgtggtgct gaccctgagc cgcatctggt acagcgccgt gaccggcaag

2101 atcgccccca aggtgagtcg acgagcaagc ccggcggatc aggcagcgtg cttgcagatt

2161 tgacttgcaa cgcccgcatt gtgtcgacga aggcttttgg ctcctctgtc gctgtctcaa

2221 gcagcatcta accctgcgtc gccgtttcca tttgcaggac gtggccgccg actgggccat

2281 ggagcgcctg cccgcccagt accagcccgt gatcctggag gcccgccagg cctacctggg

2341 ccaggaggag gaccgcctgg ccagccgcgc cgaccagctg gaggagttcg tgcactacgt

2401 gaagggcgag atcaccaagg tggtgggcaa gtaaatgccc tggcggcaca gttttgatgt

2461 accaataggg atatcgggat acgatcgaat attatccgtt taaactatca gtgtttgaca

2521 ggatatattg gcgggtaaac ctaagagaaa agagcgttta ttagaataat cggatattta

2581 aaagggcgtg aaaaggttta tccgttcgtc catttgtatg tgccagccgc ctttgcgacg

2641 ctcaccgggc tggttgccct cgccgctggg ctggcggccg tctatggccc tgcaaacgcg

2701 ccagaaacgc cgtcgaagcc gtgtgcgaga caccgcggcc gccggcgttg tggatacctc

2761 gcggaaaact tggccctcac tgacagatga ggggcggacg ttgacacttg aggggccgac

2821 tcacccggcg cggcgttgac agatgagggg caggctcgat ttcggccggc gacgtggagc

2881 tggccagcct cgcaaatcgg cgaaaacgcc tgattttacg cgagtttccc acagatgatg

2941 tggacaagcc tggggataag tgccctgcgg tattgacact tgaggggcgc gactactgac

3001 agatgagggg cgcgatcctt gacacttgag gggcagagtg ctgacagatg aggggcgcac

3061 ctattgacat ttgaggggct gtccacaggc agaaaatcca gcatttgcaa gggtttccgc

3121 ccgtttttcg gccaccgcta acctgtcttt taacctgctt ttaaaccaat atttataaac

3181 cttgttttta accagggctg cgccctgtgc gcgtgaccgc gcacgccgaa ggggggtgcc

3241 cccccttctc gaaccctccc ggcccgctaa cgcgggcctc ccatcccccc aggggctgcg

3301 cccctcggcc gcgaacggcc tcaccccaaa aatggcagcg ctggccaatt cccgagtgcg

3361 cggaacccct atttgtttat ttttctaaat acattcaaat atgtatccgc tcatgagaca

3421 ataaccctga taaatgcttc aataatattg aaaaaggaag agtatgagcc atattcaacg

3481 ggaaacgtct tgctctaggc cgcgattaaa ttccaacatg gatgctgatt tatatgggta

3541 taaatgggct cgcgataatg tcgggcaatc aggtgcgaca atctatcgat tgtatgggaa

3601 gcccgatgcg ccagagttgt ttctgaaaca tggcaaaggt agcgttgcca atgatgttac

3661 agatgagatg gtcagactaa actggctgac ggaatttatg cctcttccga ccatcaagca

3721 ttttatccgt actcctgatg atgcatggtt actcaccact gcgatccccg ggaaaacagc

3781 attccaggta ttagaagaat atcctgattc aggtgaaaat attgttgatg cgctggcagt

3841 gttcctgcgc cggttgcatt cgattcctgt ttgtaattgt ccttttaaca gcgatcgcgt

3901 atttcgtctc gctcaggcgc aatcacgaat gaataacggt ttggttgatg cgagtgattt

3961 tgatgacgag cgtaatggct ggcctgttga acaagtctgg aaagaaatgc ataaactttt

4021 gccattctca ccggattcag tcgtcactca tggtgatttc tcacttgata accttatttt

4081 tgacgagggg aaattaatag gttgtattga tgttggacga gtcggaatcg cagaccgata

4141 ccaggatctt gccatcctat ggaactgcct cggtgagttt tctccttcat tacagaaacg

4201 gctttttcaa aaatatggta ttgataatcc tgatatgaat aaattgcagt ttcatttgat

4261 gctcgatgag tttttctaac tgtcagacca agtttactca tatatacttt agattgattt

4321 aaaacttcat ttttaattta aaaggatcta ggtgaagatc ctttttgata atctcatgac

4381 caaaatccct taacgtgagt tttcgttcca ctgagcgtca gaccccgtag aaaagatcaa

4441 aggatcttct tgagatcctt tttttctgcg cgtaatctgc tgcttgcaaa caaaaaaacc

4501 accgctacca gcggtggttt gtttgccgga tcaagagcta ccaactcttt ttccgaaggt

4561 aactggcttc agcagagcgc agataccaaa tactgtcctt ctagtgtagc cgtagttagg

4621 ccaccacttc aagaactctg tagcaccgcc tacatacctc gctctgctaa tcctgttacc

4681 agtggctgct gccagtggcg ataagtcgtg tcttaccggg ttggactcaa gacgatagtt

4741 accggataag gcgcagcggt cgggctgaac ggggggttcg tgcacacagc ccagcttgga

4801 gcgaacgacc tacaccgaac tgagatacct acagcgtgag ctatgagaaa gcgccacgct

4861 tcccgaaggg agaaaggcgg acaggtatcc ggtaagcggc agggtcggaa caggagagcg

4921 cacgagggag cttccagggg gaaacgcctg gtatctttat agtcctgtcg ggtttcgcca

4981 cctctgactt gagcgtcgat ttttgtgatg ctcgtcaggg gggcggagcc tatggaaaaa

5041 cgccagcaac gcggcctttt tacggttcct ggcagatcct agatgtggcg caacgatgcc

5101 ggcgacaagc aggagcgcac cgacttcttc cgcatcaagt gttttggctc tcaggccgag

5161 gcccacggca agtatttggg caaggggtcg ctggtattcg tgcagggcaa gattcggaat

5221 accaagtacg agaaggacgg ccagacggtc tacgggaccg acttcattgc cgataaggtg

5281 gattatctgg acaccaaggc accaggcggg tcaaatcagg aataagggca cattgccccg

5341 gcgtgagtcg gggcaatccc gcaaggaggg tgaatgaatc ggacgtttga ccggaaggca

5401 tacaggcaag aactgatcga cgcggggttt tccgccgagg atgccgaaac catcgcaagc

5461 cgcaccgtca tgcgtgcgcc ccgcgaaacc ttccagtccg tcggctcgat ggtccagcaa

5521 gctacggcca agatcgagcg cgacagcgtg caactggctc cccctgccct gcccgcgcca

5581 tcggccgccg tggagcgttc gcgtcgtctt gaacaggagg cggcaggttt ggcgaagtcg

5641 atgaccatcg acacgcgagg aactatgacg accaagaagc gaaaaaccgc cggcgaggac

5701 ctggcaaaac aggtcagcga ggccaagcag gccgcgttgc tgaaacacac gaagcagcag

5761 atcaaggaaa tgcagctttc cttgttcgat attgcgccgt ggccggacac gatgcgagcg

5821 atgccaaacg acacggcccg ctctgccctg ttcaccacgc gcaacaagaa aatcccgcgc

5881 gaggcgctgc aaaacaaggt cattttccac gtcaacaagg acgtgaagat cacctacacc

5941 ggcgtcgagc tgcgggccga cgatgacgaa ctggtgtggc agcaggtgtt ggagtacgcg

6001 aagcgcaccc ctatcggcga gccgatcacc ttcacgttct acgagctttg ccaggacctg

6061 ggctggtcga tcaatggccg gtattacacg aaggccgagg aatgcctgtc gcgcctacag

6121 gcgacggcga tgggcttcac gtccgaccgc gttgggcacc tggaatcggt gtcgctgctg

6181 caccgcttcc gcgtcctgga ccgtggcaag aaaacgtccc gttgccaggt cctgatcgac

6241 gaggaaatcg tcgtgctgtt tgctggcgac cactacacga aattcatatg ggagaagtac

6301 cgcaagctgt cgccgacggc ccgacggatg ttcgactatt tcagctcgca ccgggagccg

6361 tacccgctca agctggaaac cttccgcctc atgtgcggat cggattccac ccgcgtgaag

6421 aagtggcgcg agcaggtcgg cgaagcctgc gaagagttgc gaggcagcgg cctggtggaa

6481 cacgcctggg tcaatgatga cctggtgcat tgcaaacgct agggccttgt ggggtcagtt

6541 ccggctgggg gttcagcagc ccctgctcgg atctgttgga ccggacagta gtcatggttg

6601 atgggctgcc tgtatcgagt ggtgattttg tgccgagctg ccggtcgggg agctgttggc

6661 tggctggtgg caggatatat tgtggtgtaa acaaattgac gcttagacaa cttaataaca

6721 ca

//

>Targeted integration of CnVs on LHCBM1 locus

LOCUS Exported 8207 bp ds-DNA circular SYN 02-AUG-2024

DEFINITION synthetic circular DNA

ACCESSION .

VERSION .

KEYWORDS .

SOURCE synthetic DNA construct

ORGANISM recombinant plasmid

REFERENCE 1 (bases 1 to 8207)

AUTHORS Trial User

TITLE Direct Submission

JOURNAL Exported Friday, Aug 2, 2024 from SnapGene Viewer 4.3.11

https://www.snapgene.com

FEATURES Location/Qualifiers

source 1..8207

/organism="recombinant plasmid"

/mol_type="other DNA"

misc_feature 37..43

/label=Insertion Event

misc_feature 51..97

/label=HA1 EcoRV

misc_feature 92..94

/label=PAM

misc_feature 95..114

/label=sgRNA1 rev

CDS 98..164

/codon_start=1

/label=LHCBM1

/translation="SDHLANPGTNNAFAYATKFTPQ"

CDS 165..182

/codon_start=1

/label=GSGSGS-Linker

/translation="GSGSGS"

CDS 183..300

/codon_start=1

/product="unnamed_input_seq"

/label=ext2A

/label=unnamed_input_seq__CDS

/translation="LLAIHPTEARHKQKIVAPVKQTLNFDLLKLAGDVESNPG"

CDS 303..320

/codon_start=1

/label=GSGSGS-Linker

/translation="GSGSGS"

CDS join(321..418,564..951,1097..1498,1644..2055,2201..2587,

2733..2809)

/codon_start=1

/product="AFN21429.1 terpene synthase Valencene synthase

[Callitropsis nootkatensis] Beekwilder 2014"

/label=AFN21429.1 terpene synthase Valencene synthase

/label=AFN21429.1 terpene synthase Valencene synthase

[Callitropsis nootkatensis] Beekwilder 2014__CDS

/translation="AEMFNGNSSNDGSSCMPVKDALRRTGNHHPNLWTDDFIQSLNSPY

SDSSYHKHREILIDEIRDMFSNGEGDEFGVLENIWFVDVVQRLGIDRHFQEEIKTALDY

IYKFWNHDSIFGDLNMVALGFRILRLNRYVASSDVFKKFKGEEGQFSGFESSDQDAKLE

MMLNLYKASELDFPDEDILKEARAFASMYLKHVIKEYGDIQESKNPLLMEIEYTFKYPW

RCRLPRLEAWNFIHIMRQQDCNISLANNLYKIPKIYMKKILELAILDFNILQSQHQHEM

KLISTWWKNSSAIQLDFFRHRHIESYFWWASPLFEPEFSTCRINCTKLSTKMFLLDDIY

DTYGTVEELKPFTTTLTRWDVSTVDNHPDYMKIAFNFSYEIYKEIASEAERKHGPFVYK

YLQSCWKSYIEAYMQEAEWIASNHIPGFDEYLMNGVKSSGMRILMIHALILMDTPLSDE

ILEQLDIPSSKSQALLSLITRLVDDVKDFEDEQAHGEMASSIECYMKDNHGSTREDALN

YLKIRIESCVQELNKELLEPSNMHGSFRNLYLNVGMRVIFFMLNDGDLFTHSNRKEIQD

AITKFFVEPIIP"

intron 419..563

/label=intron

intron 952..1096

/label=intron

intron 1499..1643

/label=intron

unsure 1988^1989

/label=bis hier hin sequenziert

intron 2056..2200

/label=intron

intron 2588..2732

/label=intron

CDS 2810..2827

/codon_start=1

/label=GSGSGS-Linker

/translation="GSGSGS"

CDS join(2828..3085,3231..3584,3730..3903)

/codon_start=1

/label=AadA Spectinomycin resistance

/translation="REAVIAEVSTQLSEVVGVIERHLEPTLLAVHLYGSAVDGGLKPHS

DIDLLVTVTVRLDETTRRALINDLLETSASPGESEILRAVEVTIVVHDDIIPWRYPAKR

ELQFGEWQRNDILAGIFEPATIDIDLAILLTKAREHSVALVGPAAEELFDPVPEQDLFE

ALNETLTLWNSPPDWAGDERNVVLTLSRIWYSAVTGKIAPKDVAADWAMERLPAQYQPV

ILEARQAYLGQEEDRLASRADQLEEFVHYVKGEITKVVGK"

intron 3086..3230

/label=rbcS2 intron 1

intron 3585..3729

/label=rbcS2 intron 1

3'UTR 3907..3944

misc_feature 3907..3944

/label=HA2 EcoRV

misc_feature 3988..4012

/label=RB T-DNA repeat

/note="right border repeat from nopaline C58 T-DNA"

rep_origin 4099..4810

/label=oriV

/note="incP origin of replication"

promoter 4831..4935

/gene="bla"

/label=AmpR promoter

CDS 4936..5751

/codon_start=1

/gene="aph(3')-Ia"

/product="aminoglycoside phosphotransferase"

/label=KanR

/note="confers resistance to kanamycin in bacteria or G418

(Geneticin(R)) in eukaryotes"

/translation="MSHIQRETSCSRPRLNSNMDADLYGYKWARDNVGQSGATIYRLYG

KPDAPELFLKHGKGSVANDVTDEMVRLNWLTEFMPLPTIKHFIRTPDDAWLLTTAIPGK

TAFQVLEEYPDSGENIVDALAVFLRRLHSIPVCNCPFNSDRVFRLAQAQSRMNNGLVDA

SDFDDERNGWPVEQVWKEMHKLLPFSPDSVVTHGDFSLDNLIFDEGKLIGCIDVGRVGI

ADRYQDLAILWNCLGEFSPSLQKRLFQKYGIDNPDMNKLQFHLMLDEFF"

rep_origin 5922..6510

/direction=RIGHT

/label=ori

/note="high-copy-number ColE1/pMB1/pBR322/pUC origin of

replication"

CDS 6846..7994

/codon_start=1

/product="trans-acting replication protein that binds to

and activates oriV"

/label=trfA

/translation="MNRTFDRKAYRQELIDAGFSAEDAETIASRTVMRAPRETFQSVGS

MVQQATAKIERDSVQLAPPALPAPSAAVERSRRLEQEAAGLAKSMTIDTRGTMTTKKRK

TAGEDLAKQVSEAKQAALLKHTKQQIKEMQLSLFDIAPWPDTMRAMPNDTARSALFTTR

NKKIPREALQNKVIFHVNKDVKITYTGVELRADDDELVWQQVLEYAKRTPIGEPITFTF

YELCQDLGWSINGRYYTKAEECLSRLQATAMGFTSDRVGHLESVSLLHRFRVLDRGKKT

SRCQVLIDEEIVVLFAGDHYTKFIWEKYRKLSPTARRMFDYFSSHREPYPLKLETFRLM

CGSDSTRVKKWREQVGEACEELRGSGLVEHAWVNDDLVHCKR"

misc_feature 8140..8164

/label=LB T-DNA repeat

/note="left border repeat from nopaline C58 T-DNA"

ORIGIN

1 taatgtactg gggttgaaaa tattcgatcg attgcctgag gcctgccgat atcttagttt

61 acccggtgat gttatgctcc tcgtgcccgc tccacaggtc cgaccacctg gccaaccccg

121 gcaccaacaa cgccttcgcc tacgccacca agttcacccc ccagggcagc ggcagcggca

181 gcctgctggc catccacccc accgaggccc gccacaagca gaagatcgtg gcccccgtga

241 agcagaccct gaacttcgac ctgctgaagc tggccggcga cgtggagagc aaccccggcc

301 ccggcagcgg cagcggcagc gccgagatgt tcaacggcaa cagcagcaac gacggcagca

361 gctgcatgcc cgtgaaggac gccctgcgcc gcaccggcaa ccaccacccc aacctgtggt

421 gagtcgacga gcaagcccgg cggatcaggc agcgtgcttg cagatttgac ttgcaacgcc

481 cgcattgtgt cgacgaaggc ttttggctcc tctgtcgctg tctcaagcag catctaaccc

541 tgcgtcgccg tttccatttg caggaccgac gacttcatcc agagcctgaa cagcccctac

601 agcgacagca gctaccacaa gcaccgcgag atcctgatcg acgagatccg cgacatgttc

661 agcaacggcg agggcgacga gttcggcgtg ctggagaaca tctggttcgt ggacgtggtg

721 cagcgcctgg gcatcgaccg ccacttccag gaggagatca agaccgccct ggactacatc

781 tacaagttct ggaaccacga cagcatcttc ggcgacctga acatggtggc cctgggcttc

841 cgcatcctgc gcctgaaccg ctacgtggcc agcagcgacg tgttcaagaa gttcaagggc

901 gaggagggcc agttcagcgg cttcgagagc agcgaccagg acgccaagct ggtgagtcga

961 cgagcaagcc cggcggatca ggcagcgtgc ttgcagattt gacttgcaac gcccgcattg

1021 tgtcgacgaa ggcttttggc tcctctgtcg ctgtctcaag cagcatctaa ccctgcgtcg

1081 ccgtttccat ttgcaggaga tgatgctgaa cctgtacaag gccagcgagc tggacttccc

1141 cgacgaggac atcctgaagg aggcccgcgc cttcgccagc atgtacctga agcacgtgat

1201 caaggagtac ggcgacatcc aggagagcaa gaaccccctg ctgatggaga tcgagtacac

1261 cttcaagtac ccctggcgct gccgcctgcc ccgcctggag gcctggaact tcatccacat

1321 catgcgccag caggactgca acatcagcct ggccaacaac ctgtacaaga tccccaagat

1381 ttacatgaag aagatcctgg agctggccat cctggacttc aacatcctgc agagccagca

1441 ccagcacgag atgaagctga tcagcacctg gtggaagaac agcagcgcca tccagctggt

1501 gagtcgacga gcaagcccgg cggatcaggc agcgtgcttg cagatttgac ttgcaacgcc

1561 cgcattgtgt cgacgaaggc ttttggctcc tctgtcgctg tctcaagcag catctaaccc

1621 tgcgtcgccg tttccatttg caggacttct tccgccaccg ccacatcgag agctacttct

1681 ggtgggccag ccccctgttc gagcccgagt tcagcacctg ccgcatcaac tgcaccaagc

1741 tgagcaccaa gatgttcctg ctggacgaca tctacgacac ctacggcacc gtggaggagc

1801 tgaagccctt caccaccacc ctgacccgct gggacgtgag caccgtggac aaccaccccg

1861 actacatgaa gatcgccttc aacttcagct acgagattta caaggagatc gccagcgagg

1921 ccgagcgcaa gcacggcccc ttcgtgtaca agtacctgca gagctgctgg aagagctaca

1981 tcgaggccta catgcaggag gccgagtgga tcgccagcaa ccacatcccc ggcttcgacg

2041 agtacctgat gaacggtgag tcgacgagca agcccggcgg atcaggcagc gtgcttgcag

2101 atttgacttg caacgcccgc attgtgtcga cgaaggcttt tggctcctct gtcgctgtct

2161 caagcagcat ctaaccctgc gtcgccgttt ccatttgcag gcgtgaagag cagcggcatg

2221 cgcatcctga tgatccacgc cctgatcctg atggacaccc ccctgagcga cgagatcctg

2281 gagcagctgg acatccccag cagcaagagc caggccctgc tgagcctgat cacccgcctg

2341 gtggacgacg tgaaggactt cgaggacgag caggcccacg gcgagatggc cagcagcatc

2401 gagtgctaca tgaaggacaa ccacggcagc acccgcgagg acgccctgaa ctacctgaag

2461 atccgcatcg agagctgcgt gcaggagctg aacaaggagc tgctggagcc cagcaacatg

2521 cacggcagct tccgcaacct gtacctgaac gtgggcatgc gcgtgatctt cttcatgctg

2581 aacgacggtg agtcgacgag caagcccggc ggatcaggca gcgtgcttgc agatttgact

2641 tgcaacgccc gcattgtgtc gacgaaggct tttggctcct ctgtcgctgt ctcaagcagc

2701 atctaaccct gcgtcgccgt ttccatttgc aggcgacctg ttcacccaca gcaaccgcaa

2761 ggagatccag gacgccatca ccaagttctt cgtggagccc atcatccccg gcagcggcag

2821 cggcagccgc gaggccgtga tcgccgaggt gagcacccag ctgagcgagg tggtgggcgt

2881 gatcgagcgc cacctggagc ccaccctgct ggccgtgcac ctgtacggca gcgccgtgga

2941 cggcggcctg aagccccaca gcgacatcga cctgctggtg accgtgaccg tgcgcctgga

3001 cgagacgacc cgccgcgccc tgatcaacga cctgctggag acgagcgcca gccccggcga

3061 gagcgagatc ctgcgcgccg tggaggtgag tcgacgagca agcccggcgg atcaggcagc

3121 gtgcttgcag atttgacttg caacgcccgc attgtgtcga cgaaggcttt tggctcctct

3181 gtcgctgtct caagcagcat ctaaccctgc gtcgccgttt ccatttgcag gtgaccatcg

3241 tggtgcacga cgacatcatc ccctggcgct accccgccaa gcgcgagctg cagttcggcg

3301 agtggcagcg caacgacatc ctggccggca tcttcgagcc cgccaccatc gacatcgacc

3361 tggccatcct gctgaccaag gcccgcgagc acagcgtggc cctggtgggc cccgccgccg

3421 aggagctgtt cgaccccgtg cccgagcagg acctgttcga ggccctgaac gagacgctga

3481 ccctgtggaa cagccccccc gactgggccg gcgacgagcg caacgtggtg ctgaccctga

3541 gccgcatctg gtacagcgcc gtgaccggca agatcgcccc caaggtgagt cgacgagcaa

3601 gcccggcgga tcaggcagcg tgcttgcaga tttgacttgc aacgcccgca ttgtgtcgac

3661 gaaggctttt ggctcctctg tcgctgtctc aagcagcatc taaccctgcg tcgccgtttc

3721 catttgcagg acgtggccgc cgactgggcc atggagcgcc tgcccgccca gtaccagccc

3781 gtgatcctgg aggcccgcca ggcctacctg ggccaggagg aggaccgcct ggccagccgc

3841 gccgaccagc tggaggagtt cgtgcactac gtgaagggcg agatcaccaa ggtggtgggc

3901 aagtaaatgc cctggcggca cagttttgat gtaccaatag ggatatcggg atacgatcga

3961 atattatccg tttaaactat cagtgtttga caggatatat tggcgggtaa acctaagaga

4021 aaagagcgtt tattagaata atcggatatt taaaagggcg tgaaaaggtt tatccgttcg

4081 tccatttgta tgtgccagcc gcctttgcga cgctcaccgg gctggttgcc ctcgccgctg

4141 ggctggcggc cgtctatggc cctgcaaacg cgccagaaac gccgtcgaag ccgtgtgcga

4201 gacaccgcgg ccgccggcgt tgtggatacc tcgcggaaaa cttggccctc actgacagat

4261 gaggggcgga cgttgacact tgaggggccg actcacccgg cgcggcgttg acagatgagg

4321 ggcaggctcg atttcggccg gcgacgtgga gctggccagc ctcgcaaatc ggcgaaaacg

4381 cctgatttta cgcgagtttc ccacagatga tgtggacaag cctggggata agtgccctgc

4441 ggtattgaca cttgaggggc gcgactactg acagatgagg ggcgcgatcc ttgacacttg

4501 aggggcagag tgctgacaga tgaggggcgc acctattgac atttgagggg ctgtccacag

4561 gcagaaaatc cagcatttgc aagggtttcc gcccgttttt cggccaccgc taacctgtct

4621 tttaacctgc ttttaaacca atatttataa accttgtttt taaccagggc tgcgccctgt

4681 gcgcgtgacc gcgcacgccg aaggggggtg cccccccttc tcgaaccctc ccggcccgct

4741 aacgcgggcc tcccatcccc ccaggggctg cgcccctcgg ccgcgaacgg cctcacccca

4801 aaaatggcag cgctggccaa ttcccgagtg cgcggaaccc ctatttgttt atttttctaa

4861 atacattcaa atatgtatcc gctcatgaga caataaccct gataaatgct tcaataatat

4921 tgaaaaagga agagtatgag ccatattcaa cgggaaacgt cttgctctag gccgcgatta

4981 aattccaaca tggatgctga tttatatggg tataaatggg ctcgcgataa tgtcgggcaa

5041 tcaggtgcga caatctatcg attgtatggg aagcccgatg cgccagagtt gtttctgaaa

5101 catggcaaag gtagcgttgc caatgatgtt acagatgaga tggtcagact aaactggctg

5161 acggaattta tgcctcttcc gaccatcaag cattttatcc gtactcctga tgatgcatgg

5221 ttactcacca ctgcgatccc cgggaaaaca gcattccagg tattagaaga atatcctgat

5281 tcaggtgaaa atattgttga tgcgctggca gtgttcctgc gccggttgca ttcgattcct

5341 gtttgtaatt gtccttttaa cagcgatcgc gtatttcgtc tcgctcaggc gcaatcacga

5401 atgaataacg gtttggttga tgcgagtgat tttgatgacg agcgtaatgg ctggcctgtt

5461 gaacaagtct ggaaagaaat gcataaactt ttgccattct caccggattc agtcgtcact

5521 catggtgatt tctcacttga taaccttatt tttgacgagg ggaaattaat aggttgtatt

5581 gatgttggac gagtcggaat cgcagaccga taccaggatc ttgccatcct atggaactgc

5641 ctcggtgagt tttctccttc attacagaaa cggctttttc aaaaatatgg tattgataat

5701 cctgatatga ataaattgca gtttcatttg atgctcgatg agtttttcta actgtcagac

5761 caagtttact catatatact ttagattgat ttaaaacttc atttttaatt taaaaggatc

5821 taggtgaaga tcctttttga taatctcatg accaaaatcc cttaacgtga gttttcgttc

5881 cactgagcgt cagaccccgt agaaaagatc aaaggatctt cttgagatcc tttttttctg

5941 cgcgtaatct gctgcttgca aacaaaaaaa ccaccgctac cagcggtggt ttgtttgccg

6001 gatcaagagc taccaactct ttttccgaag gtaactggct tcagcagagc gcagatacca

6061 aatactgtcc ttctagtgta gccgtagtta ggccaccact tcaagaactc tgtagcaccg

6121 cctacatacc tcgctctgct aatcctgtta ccagtggctg ctgccagtgg cgataagtcg

6181 tgtcttaccg ggttggactc aagacgatag ttaccggata aggcgcagcg gtcgggctga

6241 acggggggtt cgtgcacaca gcccagcttg gagcgaacga cctacaccga actgagatac

6301 ctacagcgtg agctatgaga aagcgccacg cttcccgaag ggagaaaggc ggacaggtat

6361 ccggtaagcg gcagggtcgg aacaggagag cgcacgaggg agcttccagg gggaaacgcc

6421 tggtatcttt atagtcctgt cgggtttcgc cacctctgac ttgagcgtcg atttttgtga

6481 tgctcgtcag gggggcggag cctatggaaa aacgccagca acgcggcctt tttacggttc

6541 ctggcagatc ctagatgtgg cgcaacgatg ccggcgacaa gcaggagcgc accgacttct

6601 tccgcatcaa gtgttttggc tctcaggccg aggcccacgg caagtatttg ggcaaggggt

6661 cgctggtatt cgtgcagggc aagattcgga ataccaagta cgagaaggac ggccagacgg

6721 tctacgggac cgacttcatt gccgataagg tggattatct ggacaccaag gcaccaggcg

6781 ggtcaaatca ggaataaggg cacattgccc cggcgtgagt cggggcaatc ccgcaaggag

6841 ggtgaatgaa tcggacgttt gaccggaagg catacaggca agaactgatc gacgcggggt

6901 tttccgccga ggatgccgaa accatcgcaa gccgcaccgt catgcgtgcg ccccgcgaaa

6961 ccttccagtc cgtcggctcg atggtccagc aagctacggc caagatcgag cgcgacagcg

7021 tgcaactggc tccccctgcc ctgcccgcgc catcggccgc cgtggagcgt tcgcgtcgtc

7081 ttgaacagga ggcggcaggt ttggcgaagt cgatgaccat cgacacgcga ggaactatga

7141 cgaccaagaa gcgaaaaacc gccggcgagg acctggcaaa acaggtcagc gaggccaagc

7201 aggccgcgtt gctgaaacac acgaagcagc agatcaagga aatgcagctt tccttgttcg

7261 atattgcgcc gtggccggac acgatgcgag cgatgccaaa cgacacggcc cgctctgccc

7321 tgttcaccac gcgcaacaag aaaatcccgc gcgaggcgct gcaaaacaag gtcattttcc

7381 acgtcaacaa ggacgtgaag atcacctaca ccggcgtcga gctgcgggcc gacgatgacg

7441 aactggtgtg gcagcaggtg ttggagtacg cgaagcgcac ccctatcggc gagccgatca

7501 ccttcacgtt ctacgagctt tgccaggacc tgggctggtc gatcaatggc cggtattaca

7561 cgaaggccga ggaatgcctg tcgcgcctac aggcgacggc gatgggcttc acgtccgacc

7621 gcgttgggca cctggaatcg gtgtcgctgc tgcaccgctt ccgcgtcctg gaccgtggca

7681 agaaaacgtc ccgttgccag gtcctgatcg acgaggaaat cgtcgtgctg tttgctggcg

7741 accactacac gaaattcata tgggagaagt accgcaagct gtcgccgacg gcccgacgga

7801 tgttcgacta tttcagctcg caccgggagc cgtacccgct caagctggaa accttccgcc

7861 tcatgtgcgg atcggattcc acccgcgtga agaagtggcg cgagcaggtc ggcgaagcct

7921 gcgaagagtt gcgaggcagc ggcctggtgg aacacgcctg ggtcaatgat gacctggtgc

7981 attgcaaacg ctagggcctt gtggggtcag ttccggctgg gggttcagca gcccctgctc

8041 ggatctgttg gaccggacag tagtcatggt tgatgggctg cctgtatcga gtggtgattt

8101 tgtgccgagc tgccggtcgg ggagctgttg gctggctggt ggcaggatat attgtggtgt

8161 aaacaaattg acgcttagac aacttaataa cacattgcgg acgtttt

//

**Notes S2** **Native and edited genes**

> native LHCBM1_Cre01.g066917

LOCUS Exported 2823 bp ds-DNA linear UNA 14-OCT-2024

DEFINITION .

ACCESSION .

VERSION .

KEYWORDS .

SOURCE natural DNA sequence

ORGANISM unspecified

REFERENCE 1 (bases 1 to 2823)

AUTHORS Thomas Baier

TITLE Direct Submission

JOURNAL Exported Monday, Oct 14, 2024 from SnapGene 5.0.8

https://www.snapgene.com

FEATURES Location/Qualifiers

source 1..2823

/organism="unspecified"

/mol_type="genomic DNA"

5'UTR 1..25

/note="color: #a6acb3"

CDS join(26..141,379..461,715..1046,1195..1364,1543..1612)

/codon_start=1

/label=LHCBM1

/note="This feature has 5 segments:

1: 26 .. 141 / #ccffcc

2: 379 .. 461 / #ccffcc

3: 715 .. 1046 / #ccffcc

4: 1195 .. 1364 / #ccffcc

5: 1543 .. 1612 / #ccffcc"

/translation="MAFALAKSSARAAVSRRSTVKVEARRTVKPASKASTPDSFWYGPE

RPLFLGAFTGEPPSYLTGEFPGDYGWDTAGLSADPETFKRYRELELIHARWAMLGALGC

IFPELLGSYGVPFGEAVWFKAGAQIFQEGGLDYLGNPNLVHAQSILAILGTQVLLMGAI

EGYRVNGGPLGEGLDKLYPGGSFDPLGLADDPDTFAELKVKEIKNGRLAMFSMFGFFVQ

AIVTGKGPLQNLSDHLANPGTNNAFAYATKFTPQ"

primer_bind 77..96

/label=RTqPCR_for

/note="color: black; sequence: TCGACCGTCAAGGTCGAGGC; added:

2024-10-14"

primer_bind complement(776..795)

/label=RTqPCR_rev

/note="color: black; sequence: CGTGGATCAGCTCCAGCTCG; added:

2024-10-14"

primer_bind 1211..1238

/label=Ctrl_CDS_for

/note="color: black; sequence:

GTGGCTCGTTCGACCCCCTGGGCCTGGC; added: 2024-10-14"

primer_bind 1211..1238

/label=IVD_for

/note="color: black; sequence:

GTGGCTCGTTCGACCCCCTGGGCCTGGC; added: 2024-10-14"

misc_feature 1496..1542

/label=HA upstream

/note="color: #a6acb3"

misc_feature 1537..1539

/label=PAM

/note="color: #ccffff"

misc_feature 1540..1559

/label=sgRNA1 rev

/note="This feature has 2 segments:

1: 1540 .. 1542 / #cc99ff

2: 1543 .. 1559 / #cc99ff"

misc_feature 1590..1592

/label=PAM

/note="color: #ffffff"

misc_feature 1593..1612

/label=sgRNA2 rev

/note="This feature has 2 segments:

1: 1593 .. 1595 / #ffffff

2: 1596 .. 1612 / #ffffff"

3'UTR 1613..1823

/note="color: #999999"

misc_feature 1613..1650

/label=HA2 downstream

/note="color: #a6acb3"

primer_bind complement(1780..1823)

/label=Ctrl_3'UTR_rev

/note="color: black; sequence:

GTCTTCTCTCAAAATTTACAAACGTTGACAAGTCCCAGCGCAGC; added:

2024-10-14"

primer_bind complement(1780..1823)

/label=IVD_rev

/note="color: black; sequence:

GTCTTCTCTCAAAATTTACAAACGTTGACAAGTCCCAGCGCAGC; added:

2024-10-14"

ORIGIN

1 acagaagtta ctcccaccag tcaaaatggc cttcgccctt gccaagtcct ccgctcgcgc

61 cgcggtgtct cgccgctcga ccgtcaaggt cgaggcgcgc cgcaccgtga agcccgcctc

121 caaggcgtcc accccggaca ggtgtgtagc tcgccaatga agtgctctgg ggaagatttg

181 cagggggaat gacagggcac gactgccgga actggccact ccggcatggg gatcctcttt

241 ccctgacttg cgcttcctgt ctggtgagct gccaccagtg gcaaagaata cagctccatg

301 tctacaatgc ggcctgccga tttcactgta tcaagctctt gacgtctgaa ccctttcgcg

361 atggcccttt gcttgcagct tctggtatgg ccctgagcgc cccctgttcc tgggcgcctt

421 cactggcgag cccccgagct acctgactgg cgagttcccc ggtaagtctt tctgtgtcgc

481 ggggttctgg gcgttcgcat gcgcaacagt gtcgcacggt cgctcttgca gcacagtcac

541 tacagatagt ccaagtccga cgcatggcga tcgggcaact gcgatttgca catgcggcaa

601 gggatctcta gctcgggctg gcgaagcctt caggacatgg agcgctgtcc agcagctggt

661 tggtgatgct ctatcctaaa ttgcccctcc cacacaccct tacttgcttt ccaggtgact

721 acggctggga caccgccggt ctgtccgctg acccggagac cttcaagcgc taccgcgagc

781 tggagctgat ccacgcccgc tgggccatgc tcggcgctct gggctgcatc ttccccgagc

841 tgctgggctc ctacggcgtg cccttcggcg aggccgtgtg gttcaaggct ggtgctcaga

901 tcttccagga gggcggtctg gactacctgg gcaaccccaa cctggtgcac gcccagtcca

961 tcctggccat cctgggcacc caggtgctgc tgatgggcgc cattgagggc taccgcgtca

1021 acggcggccc cctgggcgag ggcctggtga gtgcggttcg ctgtgaaggg aagcttccct

1081 caaaacacat gggacagttt cggtttgtgt tgtctggttg cgtcagtagc aatgcacggt

1141 tcgtggacgt tgtgcatggc atcattgccc acccttgctc cctccgcgtt gcaggacaag

1201 ctgtaccccg gtggctcgtt cgaccccctg ggcctggctg acgaccccga caccttcgct

1261 gagctgaagg tgaaggagat caagaacggc cgcctggcca tgttctccat gttcggcttc

1321 ttcgttcagg ccatcgtgac cggcaagggc cccctgcaga acctgtgagt agggatgggg

1381 gcctagggtg acataacatg agtcgcggtg tgtgcagcac gctgcttgcc ataaatgccc

1441 agccgcgcta acctacgcgg attaaacatt aattcgagtt gatacttgca ctcccatctt

1501 agtttacccg gtgatgttat gctcctcgtg cccgctccac aggtccgacc acctggccaa

1561 ccccggcacc aacaacgcct tcgcctacgc caccaagttc accccccagt aaatgccctg

1621 gcggcacagt tttgatgtac caatagggat gcaggtctga gcggtttatt tgggtcgtct

1681 tgtgtggtct ggtggagctt gagttgtttg ggagcggtgg gttttgtgtg cggtctggcc

1741 gtgcagcagg caaggtcccg acaggcgcag gagcggctag ctgcgctggg acttgtcaac

1801 gtttgtaaat tttgagagaa gacgtgcatc cgaatctcac cccacgttcc ggatggggaa

1861 tggatctggg acgaactgac aggacacagg cgggatagca aaagctgcag caaaccgtta

1921 ggtacgactg ctacacagca agtgagcgga atagcaccga acttctagca ccccggtagt

1981 agataactga ggtagtacca gttgcatggc taaagttagg cagagtcaaa gttgggttgt

2041 cgtcctgtcg agactccatc cgagcgaacc gaagaaaatg tcgaaagcag atcacggtac

2101 tgttgcactg gcgcaggcgc agacccggca gccgccgccg cgtccacgcc acctccagag

2161 tagagatggc gtattcgcag ctcatgctca gctgccgacg ccacccgcgc gctgggtatc

2221 cccgcctgac agcgcttgaa tgccggctgc tccgctagcc atgcctgcag ccacagccga

2281 cggcctgtct ggactgtgca gtggcggtag ccagcgccag acagccgggt aaagcaggcg

2341 cgctgctggc ggcgccgttc tgttgcggca cgctgatgcg tgtgctggcg cgtgccccgc

2401 cactctcgcc tcttgccctt ccacctcccg ccccccgacg ccgtcgctgc tgtcgctgct

2461 gccgccgctg ctgccgcttc cgccgtcgct gcagtggggg ctgggcaggc ccaggagcct

2521 ggccatctgg gccacgccag atgtgcgcgg ctcgtggccg gccactgtag ctgtgcggtg

2581 agaagcctgg cattgacccg acacaggcca ccaggtagtt gcctacgccg ggatcaacgc

2641 ccacgatgcg catggtgcta gggttgccag ctcaaagaca gccagcggat cgccggccgc

2701 gaccacgccc ggcactgggt tgtccccggg tgcccggccc ggcccgccac ggggcccacg

2761 ctggtcaccg tcttcgttgc tgctccctcc gctgctgtgt gcccgcctgc cgccacgccc

2821 gcg

//

> native RBCS2 Cre02.g120150

LOCUS Exported 2995 bp ds-DNA linear UNA 14-OCT-2024

DEFINITION .

ACCESSION .

VERSION .

KEYWORDS .

SOURCE natural DNA sequence

ORGANISM unspecified

REFERENCE 1 (bases 1 to 2995)

AUTHORS Thomas Baier

TITLE Direct Submission

JOURNAL Exported Monday, Oct 14, 2024 from SnapGene 5.0.8

https://www.snapgene.com

FEATURES Location/Qualifiers

source 1..2995

/organism="unspecified"

/mol_type="genomic DNA"

5'UTR 501..849

/note="color: #999999"

CDS join(850..1013,1159..1238,1568..1653,1892..2119)

/codon_start=1

/label=rbcS2 CDS

/note="This feature has 4 segments:

1: 850 .. 1013 / #ccffcc

2: 1159 .. 1238 / #ccffcc

3: 1568 .. 1653 / #ccffcc

4: 1892 .. 2119 / #ccffcc"

/translation="MAAVIAKSSVSAAVARPARSSVRPMAALKPAVKAAPVAAPAQANQ

MMVWTPVNNKMFETFSYLPPLSDEQIAAQVDYIVANGWIPCLEFAESDKAYVSNESAIR

FGSVSCLYYDNRYWTMWKLPMFGCRDPMQVLREIVACTKAFPDAYVRLVAFDNQKQVQI

MGFLVQRPKSARDWQPANKRSV"

intron 1014..1158

/label=rbcS2i1

/note="color: #------"

primer_bind 1213..1232

/label=RTqPCR_for

/note="color: black; sequence: CCAGGTCGACTACATTGTCG; added:

2024-10-14"

intron 1239..1567

/label=rbcS2i2

/note="color: #------"

primer_bind 1630..1673

/label=IVD_for

/note="color: black; sequence:

GGCCGAAGACTATGCCGATATCCGCTTCGGCAGCGTGTCTTGCGTAAGTCTGGCGAGAG

CCCG; added: 2024-10-14"

intron 1654..1891

/label=rbcS2i3

/note="color: #------"

primer_bind 1904..1928

/label=Ctrl_CDS_for

/note="color: black; sequence: AACCGCTACTGGACCATGTGGAAGC;

added: 2024-10-14"

primer_bind complement(1954..1973)

/label=RTqPCR_rev

/note="color: black; sequence: TCTCGCGCAGCACCTGCATG; added:

2024-10-14"

misc_feature 2030..2076

/label=HA upstream

/note="color: #a6acb3"

misc_feature 2059..2061

/label=PAM

/note="color: #ffffff"

misc_feature 2062..2081

/label=sgRNA3

/note="This feature has 2 segments:

1: 2062 .. 2064 / #ffffff

2: 2065 .. 2081 / #ffffff"

misc_feature 2070..2089

/label=sgRNA1

/note="This feature has 2 segments:

1: 2070 .. 2086 / #ffffff

2: 2087 .. 2089 / #ffffff"

misc_feature 2071..2073

/label=PAM

/note="color: #ccffff"

misc_feature 2074..2093

/label=sgRNA2

/note="This feature has 2 segments:

1: 2074 .. 2076 / #cc99ff

2: 2077 .. 2093 / #cc99ff"

misc_feature 2083..2085

/label=PAM

/note="color: #ffffff"

misc_feature 2086..2105

/label=sgRNA4

/note="This feature has 2 segments:

1: 2086 .. 2088 / #ffffff

2: 2089 .. 2105 / #ffffff"

misc_feature 2090..2092

/label=PAM

/note="color: #ffffff"

3'UTR 2120..2495

/note="color: #999999"

misc_feature 2120..2170

/label=HA downstream

/note="color: #a6acb3"

primer_bind complement(2283..2312)

/label=Ctrl_3'UTR_rev

/note="color: black; sequence:

GAGAAAGAGGCCAAAATCAACGGAGGATCG; added: 2024-10-14"

primer_bind complement(2303..2340)

/label=IVD_rev

/note="color: black; sequence:

ACGCGTACTAGTCGCTTCAAATACGCCCAGCCCGCCCATGGAGAAAGAGG; added:

2024-10-14"

ORIGIN

1 tacgcaaacc gcacccgttc caccgtccta cgccgatccc gtcaagtccc gtcctagcgc

61 cattggtgga ttggtggacc gaacttcgga gtcccctgca cgatggtagt accgcactgt

121 ctcagtgtgt acaaatgatg atgaacccag tgccccaggg gagtggtgaa ctacgcagcc

181 cacgtcaagc aagccgcgac cgtcggcaca acccggatcg ccgcatgcgc cggcgcacgg

241 gtctatacat tcgacgcgag ccaggtaaaa ctcttccaca tacctcttag aggcgacacg

301 gcgccagaaa cgacgaaaaa ctggacaaac ggcaggaaca ttgtctgttt cctagcaaca

361 ccgcgagagc ggcccagatg ccccgcctgc cgtcctatga tacttcgtga cagatgaagg

421 taattggcat gctttgcgcg ccagccgggg ccgccgcgac gggggcgtat attagttgtg

481 tcacgccacg gtttgaactc gcccgcgtgg ccgagctcgt tagttttgat aaaacccagc

541 cttaatagcg tcgcgaacgt cctgagaatg caaagtgact atcgtgcgcg tgcacccgtg

601 ccgcatcctc actctgcgtg caagcccggc ttcccgggcg cgccagaagg agcgcagcca

661 aaccaggatg atgtttgatg gggtatttga gcacttgcaa cccttatccg gaagccccct

721 ggcccacaaa ggctaggcgc caatgcaagc agttcgcatg cagcccctgg agcggtgccc

781 tcctgataaa ccggccaggg ggcctatgtt ctttactttt ttacaagaga agtcactcaa

841 catcttaaaa tggccgccgt cattgccaag tcctccgtct ccgcggccgt ggcccgcccg

901 gcccgctcca gcgtgcgccc catggccgcg ctgaagcccg ccgtcaaggc cgcccccgtg

961 gctgccccgg ctcaggccaa ccagatgatg gtctggaccc cggtcaacaa caagtgagtc

1021 gacgagcaag cccggcggat caggcagcgt gcttgcagat ttgacttgca acgcccgcat

1081 tgtgtcgacg aaggcttttg gctcctctgt cgctgtctca agcagcatct aaccctgcgt

1141 cgccgtttcc atttgcagga tgttcgagac cttctcctac ctgccccccc tgagcgacga

1201 gcagatcgcc gcccaggtcg actacattgt cgccaacggt gagcttgcgg ggttgcgagc

1261 aacactccag caacgaacag tgcccaagtc aggaatctgc agtcagcctg ggctttcggc

1321 ggctttttct tgggcaaaca gcttgcactc atgccagcgc ggcttgtcca gcctcacttg

1381 agctttccag ctgctaccag ccgggctata cgacagcgac agagccatag cgtggaatca

1441 cttatttggg ttgccgaagt agcggtcgga gcgtgagttc ttggtcaagc cgccccttat

1501 ccggttcctg tccgtgtctt tgtccctcgt tcacccttcg cggcaccctt catccccttg

1561 cttgcaggct ggatcccctg cctggagttc gctgagtcgg acaaggccta cgtgtccaac

1621 gagtcggcca tccgcttcgg cagcgtgtct tgcgtaagtc tggcgagagc ccgacgggtc

1681 cactgtggca ctgggttagc ttttggcaca cgggtccact gtggcactgg ttagcttggc

1741 accgggacag cgcctatctc accgcgggga actgacgcat acccctgctc gtgcttcagc

1801 acggaaaagc aaggggccca attccatctt tggtggttct gtgcgctggt gactgaacct

1861 cttctccctc ccatttcccg tgcgcccgca gctgtactac gacaaccgct actggaccat

1921 gtggaagctg cccatgttcg gctgccgcga ccccatgcag gtgctgcgcg agatcgtcgc

1981 ctgcaccaag gccttccccg atgcctacgt gcgcctggtg gccttcgaca accagaagca

2041 ggtgcagatc atgggcttcc tggtccagcg ccccaagtct gcccgcgact ggcagcccgc

2101 caacaagcgc tccgtgtaaa tggaggcgct cgttgatctg agccttgccc cctgacgaac

2161 ggcggtggat ggaagatact gctctcaagt gctgaagcgg tagcttagct ccccgtttcg

2221 tgctgatcag tctttttcaa cacgtaaaaa gcggaggagt tttgcaattt tgttggttgt

2281 aacgatcctc cgttgatttt ggcctctttc tccatgggcg ggctgggcgt atttgaagcg

2341 cttttggaaa agttgctgcg gggttcatca gctgaagggg actcggttcg cagatcagtt

2401 acacactaaa gaacggcggg tagcaacacc agcaaacgtg acgaaacgga accgtgcagc

2461 aaaggtggag acagcatttg cagtaacctg cagtgacgaa catgagtcag ttgttcccgg

2521 tgcccctttg cttaatctgc atagaatgat agccagctac ctagtgccaa cgtcgatagg

2581 gggcgagatt gggtgtacaa aacgtacatt ggtgagtaaa ggccttactt ggggtcctcc

2641 gcgctggcgc tggcgcgtgt tccgcacctg ttctaaacga tgcaaacctt tgattctgcc

2701 ccgctgccca cttcctgcag ctcccaaccc cgcctcgtat gatgccgttc cggcattgtg

2761 tccttgatga ccgtgctgta tggtacaagc tgtgcccatg actgcagctt ctacgctgca

2821 gtgcatcacg cctcctgtcc ctccctccct cccttacatg tcgtgctggg caccggtggc

2881 gctggtgttc tccaggttgg tttcgggcgc atcctttctg gtagtcccaa cgccagcccg

2941 gccggcgtca tccagcccag ccatcccaat accgcagcca gcttccgtca gccag

//

> native RPL10A Cre02.g101350

LOCUS Exported 4183 bp ds-DNA linear UNA 14-OCT-2024

DEFINITION .

ACCESSION .

VERSION .

KEYWORDS .

SOURCE natural DNA sequence

ORGANISM unspecified

REFERENCE 1 (bases 1 to 4183)

AUTHORS Thomas Baier

TITLE Direct Submission

JOURNAL Exported Monday, Oct 14, 2024 from SnapGene 5.0.8

https://www.snapgene.com

FEATURES Location/Qualifiers

source 1..4183

/organism="unspecified"

/mol_type="genomic DNA"

5'UTR 501..640

/note="color: #999999"

CDS join(641..683,924..1187,1376..1500,2453..2665)

/codon_start=1

/label=RPL10 CDS

/note="This feature has 4 segments:

1: 641 .. 683 / #ccffcc

2: 924 .. 1187 / #ccffcc

3: 1376 .. 1500 / #ccffcc

4: 2453 .. 2665 / #ccffcc"

/translation="MSKISNDVLRESVSALVEGAKTKPRKFQETVELQIGLKNYDPQKD

KRFSGSVRLPFVPRPRMRVCVLGDVKHCEQAGAIGVDAKGVEDLKKLNKNKKLVKKLAQ

AYHAFLASDSVIKQIPRLLGPGLNKAGKFPAPINKNLEEMVLDTKCSIKFQLKKVLCMG

VAVANVGMTEGEIRTNIMYAINFLVSLLKKNWQNVRCLYIKSTMGKPIRIY"

primer_bind 663..682

/label=RTqPCR_for

/note="color: black; sequence: TTCTCCGCGAGAGTGTCTCC; added:

2024-10-14"

intron 684..923

/label=RPL10i1

/note="color: #------"

primer_bind complement(1069..1088)

/label=RTqPCR_rev

/note="color: black; sequence: CATCACCCAGCACGCACACG; added:

2024-10-14"

intron 1188..1375

/label=RPL10i2

/note="color: #------"

intron 1501..2452

/label=RPL10i3

/note="color: #------"

primer_bind 2428..2452

/label=Ctrl_CDS_for

/note="color: black; sequence: GCTTGCCTTGCTCTTCCCTGCACAG;

added: 2024-10-14"

primer_bind 2428..2452

/label=IVD_for

/note="color: black; sequence: GCTTGCCTTGCTCTTCCCTGCACAG;

added: 2024-10-14"

misc_feature 2570..2637

/label=HA upstream

/note="color: #a6acb3"

misc_feature 2578..2580

/label=PAM

/note="color: #ffffff"

misc_feature 2581..2600

/label=sgRNA1

/note="This feature has 2 segments:

1: 2581 .. 2583 / #ffffff

2: 2584 .. 2600 / #ffffff"

misc_feature 2620..2639

/label=sgRNA3

/note="This feature has 2 segments:

1: 2620 .. 2636 / #ffffff

2: 2637 .. 2639 / #ffffff"

misc_feature 2621..2640

/label=sgRNA 2

/note="This feature has 2 segments:

1: 2621 .. 2637 / #cc99ff

2: 2638 .. 2640 / #cc99ff"

misc_feature 2640..2642

/label=PAM

/note="color: #ffffff"

misc_feature 2641..2643

/label=PAM

/note="color: #ccffff"

3'UTR 2666..3683

/note="color: #999999"

misc_feature 2666..2725

/label=HA downstream

/note="color: #a6acb3"

primer_bind complement(2817..2846)

/label=Ctrl_3'UTR_rev

/note="color: black; sequence:

CTTGGTAATGTTCGTGCGACGAGTTATCCC; added: 2024-10-14"

primer_bind complement(2985..3009)

/label=IVD_rev

/note="color: black; sequence: GGATCAGAAAGCATTCTCGGTGCGG;

added: 2024-10-14"

ORIGIN

1 gcacaaatac acacactacg ggggcggcat gcaactgtgg taaacagggg ggtgctctta

61 aagacacact ctccgggtgc tcgtggagcg tgaaggaaga ttgctgtaga cgctagagcg

121 ggctcgcggc attccgagtc agctgcttag tatagcgctt agtgcataag cgaagcagcg

181 gtggtgcatt caggaacatg tgtgacgcca ggtctgtaac aaaggactac agcatctcgg

241 cgactcggcc cgcaaaccct gctacaccgg cgacggggca gtgggggaca ttccgttgtt

301 ccgtgtagtg caggctatga ttgtgaaatt acaagaaggg acagtccaac caagctaggg

361 tgcccaggct atgcgcagca agttcgcatg ccaggcgcga gggtggcggg aacgggcggg

421 tcagggtgac gggttgaggg caaggcacgg tacccgccag ccaactgggc cttactttca

481 tcatagggaa agcataaatc ataacagtgt agtttatatt atgcatgatg tcttccgcag

541 aagaggcacc gtgatgccca ccgcccccat gcatcaattg tgagggtcaa gagcgcccgc

601 ggacccctgg acattccttt tccttgggtg aagcacgaaa atgagcaaga tctcgaacga

661 cgttctccgc gagagtgtct ccggtgagct ctttttctga gtagaaactt tcctttctgg

721 gcttgcttct tcggcgactt gacgcgtcta gcttagctcg ctcgattttc gcttctacgt

781 agtgttaacg atagcttatg aagcaagttg acaattaagg cacagggcag gagtcgccgc

841 cggcacaagt cgcgccgggt tttacgctcg tcgcacgcgc tgctgacgct ctgtaatttt

901 atgttgcctc ccgtttattg cagccctggt ggagggggct aagaccaagc cccggaagtt

961 ccaggagacc gtggagctgc agattggtct gaagaactac gacccccaga aggacaagcg

1021 tttcagcggc tccgtccgcc tgcccttcgt gcctcgcccc cgcatgcgcg tgtgcgtgct

1081 gggtgatgtg aagcactgcg agcaggccgg cgccattggc gtcgacgcca agggtgtgga

1141 ggacctgaag aagctgaaca agaacaagaa gctggtcaag aagctgggtg agttggggca

1201 tcgcttgagg gaggcgcgaa agtggcggcc gagaatggct gggttgccag gcggttccgt

1261 gcgacatact caccgcaaca ttgcttttaa taatacggtg gtatcagatt ttgatgagcg

1321 cgcggattgc cagacgtgag ctaacccgac cccgacggcc ttcctcgccc cacagcccag

1381 gcgtaccacg ccttcctggc ttcggactcc gtcatcaagc agattccccg tctgctgggc

1441 cccggtctga acaaggccgg caagttccct gctcctatca acaagaacct ggaggagatg

1501 gtgagcgaca aacgcaacgc ccttgggttg gcagggatgg gacgggggca ggcagggtgg

1561 gctgctcact gactgcagtg gtaagggttg aattatggca cagcttgaca taactgcgcg

1621 ctggaaagca ttggagctgg agcggagcgg agcggacggg agcctggaag ctcggaggct

1681 ggcgcagagc gtattggcgg ccggtgggac aggggacggg tctggcgcac agggcagcag

1741 gctgcggggc cgacaggagg caggagcagg cgcttgcctt aatatgcgaa agccaggcga

1801 acccagctgg cgagaataga tggaccgcag ccctgggcgg cctcgggcag cggcgcagaa

1861 gctggcggca gtttgccagc ttctacaccg ctggactggg cagcaggggc tcactgcagg

1921 ctggcgaggg cagcggccat gttgcgtcat tagcgcaatg gattcaatgc acgccgtcag

1981 cggttatgcc atggcgtgat tggacggacc acctcgcgca gcctcagccg gctgctgcgg

2041 ctgcaagtgg gtgcgcttgc agcgagccaa ggccagcggg cggctgggca cagcaggggg

2101 acctctgctg ctaagcgcca cagcgtggat cctctactgc gcacggcgcg gacggctcag

2161 tgcaagaaga cggagggact cagcgacagc atgatgatca gccgctgcag cggcggcact

2221 cgctcgggtg ggctggccaa gggctggaac cgcggtctgg aggaaagccg ctcggcagca

2281 gcagcagcaa cagcctggct tccgggctcc cccggttggc ccaatgagcg caggccagcg

2341 cgtggatgcg caagcgccag acttgaacac caaatattgt agcacaacca caggtccctg

2401 accgccctgc ccgccctgcc gtgccgcgct tgccttgctc ttccctgcac aggtgctgga

2461 caccaagtgc agcatcaagt tccagctgaa gaaggtgctg tgcatgggcg tggccgtggc

2521 caacgtgggc atgaccgagg gcgagatccg taccaacatc atgtacgcca tcaacttcct

2581 ggtgtcgctg ctcaagaaga actggcagaa cgtgcgctgc ctgtacatca agagcaccat

2641 gggcaagccc atccgcatct actaagcaat tggtgggtgc gggccggcgg cccgttgggg

2701 gctgcgcggc cgcgccgggg ttccccgggc gtgctggtca cacgcccggc ccggctgcgg

2761 tgttggcgcg tggctcgagt tggttcttca gttcctggaa ctctccacca tggtcaggga

2821 taactcgtcg cacgaacatt accaaggacc ggggcgcttt tagcctgcct gccctcgtgg

2881 ctggcgaggc ggcgcttgcc ggcttccatt gggagttcac atgagtgact catgctcgtt

2941 cggcaggctt cactgcgttt ggaaaggtta catgacgaca agctccgcac cgagaatgct

3001 ttctgatcct gcgggcctct ggcttgcgcg gtgtgtttgt aacgccggcg tagtgatcat

3061 gcctcgtgcc tcgggttcgt tctttggggg ttgttggtgg aaaacactga gcagcgtcag

3121 ccaaatatag acgacggcag gagccggccc tgcacctaga gcgttgggcc aggcactggg

3181 tacgcagccc acccagattc ttgaacttga gcagcacagc atctaacagc cttgattgac

3241 gtgtgttgcg gaactaacgc gtacgtagac cagagcggaa ggcggagtgg gcttgcaggc

3301 ggcacgagag caggcagtca gtagcgcaag ttgcgaatcc gcatgttccc tcgtaacgga

3361 ccgtgaacac tgtggacggt cgcgcccatg gctagcggac gtaaggtggc tgctgtattg

3421 ggatggctgg gctgccgggc tgggcgacgg cggccggcct tgatgtgaca gagaaccgca

3481 catgtcatgc agtacacggg gtggcgcatg gctgggcacc aggttggacc acattggcca

3541 ttgcaagtgg cgttgcggtg tgtctgtgtg gtgccatcgt ctggccgggc gcccggagcg

3601 tgagggattc gcatcagcaa acttccagtc agcaagcaac ggaacagcga cggtacggta

3661 cagaaactcc ggcataacgg cattcgatca gccgtgcact cctgccctgt cgttgcaagt

3721 tgttgccatc tgctggggta tgggaatcag ctgcaccctg cgggacttca ccaacctgcc

3781 ttcacactgt cccaccgcac actcatgcca aaggaaatgt ccttccgcag ggttggcttg

3841 ggtcaggagg ggagaagacg aacagccatc ggcatgagcg cttcttgccc gcacggagca

3901 cactatggca ccaactggtg cccaagaggt attgccctgc cctcagcttg cttctaagct

3961 tcaactgtcc ccaaggctac gcacacaagc gcctgacttc agcacatcac caccacaaga

4021 gtggaaactt ccaacgcctg cctgagatac acacacattc acacactcca ggacaggcca

4081 catcacgttg tcccaagccc cagtcagttg cagtgtcagt gcccacctgc ctacctgccg

4141 acctccgata tatacttgcc tgactgcccg gccttgctgc tcg

//

> edited LHCBM1_YFP_aadA_Cre01.g066917

LOCUS Exported 5077 bp ds-DNA linear UNA 14-OCT-2024

DEFINITION natural linear DNA

ACCESSION .

VERSION .

KEYWORDS .

SOURCE natural DNA sequence

ORGANISM unspecified

REFERENCE 1 (bases 1 to 5077)

AUTHORS Thomas Baier

TITLE Direct Submission

JOURNAL Exported Monday, Oct 14, 2024 from SnapGene 5.0.8

https://www.snapgene.com

FEATURES Location/Qualifiers

source 1..5077

/organism="unspecified"

/mol_type="genomic DNA"

5'UTR 1..25

CDS join(26..141,379..461,715..1046,1195..1364,1543..1609)

/codon_start=1

/label=LHCBM1

/translation="MAFALAKSSARAAVSRRSTVKVEARRTVKPASKASTPDSFWYGPE

RPLFLGAFTGEPPSYLTGEFPGDYGWDTAGLSADPETFKRYRELELIHARWAMLGALGC

IFPELLGSYGVPFGEAVWFKAGAQIFQEGGLDYLGNPNLVHAQSILAILGTQVLLMGAI

EGYRVNGGPLGEGLDKLYPGGSFDPLGLADDPDTFAELKVKEIKNGRLAMFSMFGFFVQ

AIVTGKGPLQNLSDHLANPGTNNAFAYATKFTPQ"

primer_bind 77..96

/label=RTqPCR_for

primer_bind complement(776..795)

/label=RTqPCR_rev

primer_bind 1211..1238

/label=Ctrl_CDS_for

primer_bind 1211..1238

/label=IVD_for

misc_feature 1496..1542

/label=HA upstream

primer_bind 1496..1525

/label=HA_upstream_for

misc_feature 1537..1539

/label=PAM

misc_feature 1540..1559

/label=sgRNA1 rev

primer_bind 1543..1600

/label=2A_extension_1

primer_bind 1580..1649

/label=2A_extension_2

misc_feature 1590..1592

/label=PAM

misc_feature 1593..1609

/label=sgRNA2 rev

CDS 1610..1627

/codon_start=1

/label=GSGSGS-Linker

/translation="GSGSGS"

primer_bind 1627..1696

/label=for

CDS 1628..1745

/codon_start=1

/product="unnamed_input_seq"

/label=ext2A

/label=unnamed_input_seq__CDS

/translation="LLAIHPTEARHKQKIVAPVKQTLNFDLLKLAGDVESNPG"

primer_bind complement(1672..1741)

/label=rev1

primer_bind complement(1675..1735)

/label=insert_upstream_rev (1)

primer_bind complement(1714..1752)

/label=rev2

primer_bind 1746..1799

/label=for (1)

CDS 1748..1765

/codon_start=1

/label=GSGSGS-Linker

/translation="GSGSGS"

CDS join(1766..1964,2110..2426,2572..2769)

/codon_start=1

/label=mVenus

/translation="VSKGEELFTGVVPILVELDGDVNGHKFSVSGEGEGDATYGKLTLK

LICTTGKLPVPWPTLVTTLGYGLQCFARYPDHMKQHDFFKSAMPEGYVQERTIFFKDDG

NYKTRAEVKFEGDTLVNRIELKGIDFKEDGNILGHKLEYNYNSHNVYITADKQKNGIKA

NFKIRHNIEDGGVQLADHYQQNTPIGDGPVLLPDNHYLSYQSKLSKDPNEKRDHMVLLE

FVTAAGITLGMDELYK"

primer_bind complement(1780..1799)

/label=insert_upstream_rev

intron 1965..2109

/label=rbcS2 intron 1

intron 2427..2571

/label=rbcS2 intron 1

primer_bind complement(2748..2787)

/label=rev

CDS 2770..2787

/codon_start=1

/label=GSGSGS-Linker

/translation="GSGSGS"

primer_bind 2784..2823

/label=for (2)

CDS join(2788..3045,3191..3544,3690..3863)

/codon_start=1

/label=AadA Spectinomycin resistance

/translation="REAVIAEVSTQLSEVVGVIERHLEPTLLAVHLYGSAVDGGLKPHS

DIDLLVTVTVRLDETTRRALINDLLETSASPGESEILRAVEVTIVVHDDIIPWRYPAKR

ELQFGEWQRNDILAGIFEPATIDIDLAILLTKAREHSVALVGPAAEELFDPVPEQDLFE

ALNETLTLWNSPPDWAGDERNVVLTLSRIWYSAVTGKIAPKDVAADWAMERLPAQYQPV

ILEARQAYLGQEEDRLASRADQLEEFVHYVKGEITKVVGK"

intron 3046..3190

/label=rbcS2 intron 1

intron 3545..3689

/label=rbcS2 intron 1

primer_bind 3741..3770

/label=insert_downstream_for

primer_bind complement(3830..3870)

/label=aadA_rev

3'UTR 3867..4077

misc_feature 3867..3904

/label=HA2 downstream

primer_bind complement(4034..4077)

/label=Ctrl_3'UTR_rev

primer_bind complement(4034..4077)

/label=IVD_rev

ORIGIN

1 acagaagtta ctcccaccag tcaaaatggc cttcgccctt gccaagtcct ccgctcgcgc

61 cgcggtgtct cgccgctcga ccgtcaaggt cgaggcgcgc cgcaccgtga agcccgcctc

121 caaggcgtcc accccggaca ggtgtgtagc tcgccaatga agtgctctgg ggaagatttg

181 cagggggaat gacagggcac gactgccgga actggccact ccggcatggg gatcctcttt

241 ccctgacttg cgcttcctgt ctggtgagct gccaccagtg gcaaagaata cagctccatg

301 tctacaatgc ggcctgccga tttcactgta tcaagctctt gacgtctgaa ccctttcgcg

361 atggcccttt gcttgcagct tctggtatgg ccctgagcgc cccctgttcc tgggcgcctt

421 cactggcgag cccccgagct acctgactgg cgagttcccc ggtaagtctt tctgtgtcgc

481 ggggttctgg gcgttcgcat gcgcaacagt gtcgcacggt cgctcttgca gcacagtcac

541 tacagatagt ccaagtccga cgcatggcga tcgggcaact gcgatttgca catgcggcaa

601 gggatctcta gctcgggctg gcgaagcctt caggacatgg agcgctgtcc agcagctggt

661 tggtgatgct ctatcctaaa ttgcccctcc cacacaccct tacttgcttt ccaggtgact

721 acggctggga caccgccggt ctgtccgctg acccggagac cttcaagcgc taccgcgagc

781 tggagctgat ccacgcccgc tgggccatgc tcggcgctct gggctgcatc ttccccgagc

841 tgctgggctc ctacggcgtg cccttcggcg aggccgtgtg gttcaaggct ggtgctcaga

901 tcttccagga gggcggtctg gactacctgg gcaaccccaa cctggtgcac gcccagtcca

961 tcctggccat cctgggcacc caggtgctgc tgatgggcgc cattgagggc taccgcgtca

1021 acggcggccc cctgggcgag ggcctggtga gtgcggttcg ctgtgaaggg aagcttccct

1081 caaaacacat gggacagttt cggtttgtgt tgtctggttg cgtcagtagc aatgcacggt

1141 tcgtggacgt tgtgcatggc atcattgccc acccttgctc cctccgcgtt gcaggacaag

1201 ctgtaccccg gtggctcgtt cgaccccctg ggcctggctg acgaccccga caccttcgct

1261 gagctgaagg tgaaggagat caagaacggc cgcctggcca tgttctccat gttcggcttc

1321 ttcgttcagg ccatcgtgac cggcaagggc cccctgcaga acctgtgagt agggatgggg

1381 gcctagggtg acataacatg agtcgcggtg tgtgcagcac gctgcttgcc ataaatgccc

1441 agccgcgcta acctacgcgg attaaacatt aattcgagtt gatacttgca ctcccatctt

1501 agtttacccg gtgatgttat gctcctcgtg cccgctccac aggtccgacc acctggccaa

1561 ccccggcacc aacaacgcct tcgcctacgc caccaagttc accccccagg gcagcggcag

1621 cggcagcctg ctggccatcc accccaccga ggcccgccac aagcagaaga tcgtggcccc

1681 cgtgaagcag accctgaact tcgacctgct gaagctggcc ggcgacgtgg agagcaaccc

1741 cggccccggc agcggcagcg gcagcgtgag caagggcgag gagctgttca ccggcgtggt

1801 gcccatcctg gtggagctgg acggcgacgt gaacggccac aagttcagcg tgagcggcga

1861 gggcgagggc gacgccacct acggcaagct gaccctgaag ctgatctgca ccaccggcaa

1921 gctgcccgtg ccctggccca ccctggtgac caccctgggc tacggtgagt cgacgagcaa

1981 gcccggcgga tcaggcagcg tgcttgcaga tttgacttgc aacgcccgca ttgtgtcgac

2041 gaaggctttt ggctcctctg tcgctgtctc aagcagcatc taaccctgcg tcgccgtttc

2101 catttgcagg cctgcagtgc ttcgcccgct accccgacca catgaagcag cacgacttct

2161 tcaagagcgc catgcccgag ggctacgtgc aggagcgcac catcttcttc aaggacgacg

2221 gtaactacaa gacccgcgcc gaggtgaagt tcgagggcga caccctggtg aaccgcatcg

2281 agctgaaggg catcgacttc aaggaggacg gcaacatcct gggccacaag ctggagtaca

2341 actacaacag ccacaacgtg tacatcaccg ccgacaagca gaagaacggc atcaaggcca

2401 acttcaagat ccgccacaac atcgaggtga gtcgacgagc aagcccggcg gatcaggcag

2461 cgtgcttgca gatttgactt gcaacgcccg cattgtgtcg acgaaggctt ttggctcctc

2521 tgtcgctgtc tcaagcagca tctaaccctg cgtcgccgtt tccatttgca ggacggcggc

2581 gtgcagctgg ccgaccacta ccagcagaac acccccatcg gcgacggccc cgtgctgctg

2641 cccgacaacc actacctgag ctaccagagc aagctgagca aggaccccaa cgagaagcgc

2701 gaccacatgg tgctgctgga gttcgtgacc gccgccggca tcaccctggg catggacgag

2761 ctgtacaagg gcagcggcag cggcagccgc gaggccgtga tcgccgaggt gagcacccag

2821 ctgagcgagg tggtgggcgt gatcgagcgc cacctggagc ccaccctgct ggccgtgcac

2881 ctgtacggca gcgccgtgga cggcggcctg aagccccaca gcgacatcga cctgctggtg

2941 accgtgaccg tgcgcctgga cgagacgacc cgccgcgccc tgatcaacga cctgctggag

3001 acgagcgcca gccccggcga gagcgagatc ctgcgcgccg tggaggtgag tcgacgagca

3061 agcccggcgg atcaggcagc gtgcttgcag atttgacttg caacgcccgc attgtgtcga

3121 cgaaggcttt tggctcctct gtcgctgtct caagcagcat ctaaccctgc gtcgccgttt

3181 ccatttgcag gtgaccatcg tggtgcacga cgacatcatc ccctggcgct accccgccaa

3241 gcgcgagctg cagttcggcg agtggcagcg caacgacatc ctggccggca tcttcgagcc

3301 cgccaccatc gacatcgacc tggccatcct gctgaccaag gcccgcgagc acagcgtggc

3361 cctggtgggc cccgccgccg aggagctgtt cgaccccgtg cccgagcagg acctgttcga

3421 ggccctgaac gagacgctga ccctgtggaa cagccccccc gactgggccg gcgacgagcg

3481 caacgtggtg ctgaccctga gccgcatctg gtacagcgcc gtgaccggca agatcgcccc

3541 caaggtgagt cgacgagcaa gcccggcgga tcaggcagcg tgcttgcaga tttgacttgc

3601 aacgcccgca ttgtgtcgac gaaggctttt ggctcctctg tcgctgtctc aagcagcatc

3661 taaccctgcg tcgccgtttc catttgcagg acgtggccgc cgactgggcc atggagcgcc

3721 tgcccgccca gtaccagccc gtgatcctgg aggcccgcca ggcctacctg ggccaggagg

3781 aggaccgcct ggccagccgc gccgaccagc tggaggagtt cgtgcactac gtgaagggcg

3841 agatcaccaa ggtggtgggc aagtaaatgc cctggcggca cagttttgat gtaccaatag

3901 ggatgcaggt ctgagcggtt tatttgggtc gtcttgtgtg gtctggtgga gcttgagttg

3961 tttgggagcg gtgggttttg tgtgcggtct ggccgtgcag caggcaaggt cccgacaggc

4021 gcaggagcgg ctagctgcgc tgggacttgt caacgtttgt aaattttgag agaagacgtg

4081 catccgaatc tcaccccacg ttccggatgg ggaatggatc tgggacgaac tgacaggaca

4141 caggcgggat agcaaaagct gcagcaaacc gttaggtacg actgctacac agcaagtgag

4201 cggaatagca ccgaacttct agcaccccgg tagtagataa ctgaggtagt accagttgca

4261 tggctaaagt taggcagagt caaagttggg ttgtcgtcct gtcgagactc catccgagcg

4321 aaccgaagaa aatgtcgaaa gcagatcacg gtactgttgc actggcgcag gcgcagaccc

4381 ggcagccgcc gccgcgtcca cgccacctcc agagtagaga tggcgtattc gcagctcatg

4441 ctcagctgcc gacgccaccc gcgcgctggg tatccccgcc tgacagcgct tgaatgccgg

4501 ctgctccgct agccatgcct gcagccacag ccgacggcct gtctggactg tgcagtggcg

4561 gtagccagcg ccagacagcc gggtaaagca ggcgcgctgc tggcggcgcc gttctgttgc

4621 ggcacgctga tgcgtgtgct ggcgcgtgcc ccgccactct cgcctcttgc ccttccacct

4681 cccgcccccc gacgccgtcg ctgctgtcgc tgctgccgcc gctgctgccg cttccgccgt

4741 cgctgcagtg ggggctgggc aggcccagga gcctggccat ctgggccacg ccagatgtgc

4801 gcggctcgtg gccggccact gtagctgtgc ggtgagaagc ctggcattga cccgacacag

4861 gccaccaggt agttgcctac gccgggatca acgcccacga tgcgcatggt gctagggttg

4921 ccagctcaaa gacagccagc ggatcgccgg ccgcgaccac gcccggcact gggttgtccc

4981 cgggtgcccg gcccggcccg ccacggggcc cacgctggtc accgtcttcg ttgctgctcc

5041 ctccgctgct gtgtgcccgc ctgccgccac gcccgcg

//

> edited RBCS2_YFP_aadA_Cre02.g120150

LOCUS Exported 5249 bp ds-DNA linear UNA 14-OCT-2024

DEFINITION .

ACCESSION .

VERSION .

KEYWORDS .

SOURCE natural DNA sequence

ORGANISM unspecified

REFERENCE 1 (bases 1 to 5249)

AUTHORS Thomas Baier

TITLE Direct Submission

JOURNAL Exported Monday, Oct 14, 2024 from SnapGene 5.0.8

https://www.snapgene.com

FEATURES Location/Qualifiers

source 1..5249

/organism="unspecified"

/mol_type="genomic DNA"

/note="color: #ffffff"

5'UTR 501..849

/note="color: #999999"

CDS join(850..1013,1159..1238,1568..1653,1892..2116)

/codon_start=1

/label=rbcS2 CDS

/note="This feature has 4 segments:

1: 850 .. 1013 / #ccffcc

2: 1159 .. 1238 / #ccffcc

3: 1568 .. 1653 / #ccffcc

4: 1892 .. 2116 / #ccffcc"

/translation="MAAVIAKSSVSAAVARPARSSVRPMAALKPAVKAAPVAAPAQANQ

MMVWTPVNNKMFETFSYLPPLSDEQIAAQVDYIVANGWIPCLEFAESDKAYVSNESAIR

FGSVSCLYYDNRYWTMWKLPMFGCRDPMQVLREIVACTKAFPDAYVRLVAFDNQKQVQI

MGFLVQRPKSARDWQPANKRSV"

intron 1014..1158

/label=rbcS2i1

/note="color: #------"

primer_bind 1213..1232

/label=RTqPCR_for

/note="color: black; sequence: CCAGGTCGACTACATTGTCG; added:

2024-10-13"

intron 1239..1567

/label=rbcS2i2

/note="color: #------"

primer_bind 1630..1673

/label=IVD_for (1)

/note="color: black; sequence:

GGCCGAAGACTATGCCGATATCCGCTTCGGCAGCGTGTCTTGCGTAAGTCTGGCGAGAG

CCCG; added: 2024-10-14"

intron 1654..1891

/label=rbcS2i3

/note="color: #------"

primer_bind 1904..1928

/label=Ctrl_CDS_for

/note="color: black; sequence: AACCGCTACTGGACCATGTGGAAGC;

added: 2024-10-13"

primer_bind complement(1954..1973)

/label=RTqPCR_rev

/note="color: black; sequence: TCTCGCGCAGCACCTGCATG; added:

2024-10-13"

misc_feature 2030..2076

/label=HA upstream

/note="color: #a6acb3"

misc_feature 2059..2061

/label=PAM

/note="color: #ffffff"

misc_feature 2062..2081

/label=sgRNA3

/note="This feature has 2 segments:

1: 2062 .. 2064 / #ffffff

2: 2065 .. 2081 / #ffffff"

misc_feature 2070..2089

/label=sgRNA1

/note="This feature has 2 segments:

1: 2070 .. 2086 / #ffffff

2: 2087 .. 2089 / #ffffff"

misc_feature 2071..2073

/label=PAM

/note="color: #ccffff"

misc_feature 2074..2093

/label=sgRNA2

/note="This feature has 2 segments:

1: 2074 .. 2076 / #cc99ff

2: 2077 .. 2093 / #cc99ff"

misc_feature 2083..2085

/label=PAM

/note="color: #ffffff"

misc_feature 2086..2105

/label=sgRNA4

/note="This feature has 2 segments:

1: 2086 .. 2088 / #ffffff

2: 2089 .. 2105 / #ffffff"

misc_feature 2090..2092

/label=PAM

/note="color: #ffffff"

CDS 2117..2134

/codon_start=1

/label=GSGSGS-Linker

/note="color: #993366"

/translation="GSGSGS"

CDS 2135..2252

/codon_start=1

/product="unnamed_input_seq"

/label=ext2A

/label=unnamed_input_seq__CDS

/note="color: #993366"

/translation="LLAIHPTEARHKQKIVAPVKQTLNFDLLKLAGDVESNPG"

primer_bind complement(2182..2242)

/label=insert_upstream_rev (1)

/note="color: black; sequence:

GCTCTCCACGTCGCCGGCCAGCTTCAGCAGGTCGAAGTTCAGGGTCTGCTTCACGGGGG

CC; added: 2024-10-14"

CDS 2255..2272

/codon_start=1

/label=GSGSGS-Linker

/note="color: #993366"

/translation="GSGSGS"

CDS join(2273..2471,2617..2933,3079..3276)

/codon_start=1

/label=mVenus

/note="This feature has 3 segments:

1: 2273 .. 2471 / #fff424

2: 2617 .. 2933 / #fff424

3: 3079 .. 3276 / #fff424"

/translation="VSKGEELFTGVVPILVELDGDVNGHKFSVSGEGEGDATYGKLTLK

LICTTGKLPVPWPTLVTTLGYGLQCFARYPDHMKQHDFFKSAMPEGYVQERTIFFKDDG

NYKTRAEVKFEGDTLVNRIELKGIDFKEDGNILGHKLEYNYNSHNVYITADKQKNGIKA

NFKIRHNIEDGGVQLADHYQQNTPIGDGPVLLPDNHYLSYQSKLSKDPNEKRDHMVLLE

FVTAAGITLGMDELYK"

primer_bind complement(2287..2306)

/label=insert_upstream_rev

/note="color: black; sequence: CCACGCCGGTGAACAGCTCC; added:

2024-10-14"

intron 2472..2616

/label=rbcS2 intron 1

/note="color: #------"

intron 2934..3078

/label=rbcS2 intron 1

/note="color: #------"

primer_bind complement(3255..3294)

/label=rev

/note="color: black; sequence:

GGCCGAAGACTAGCTGCCGCTGCCGCTGCCCTTGTACAGCTCGTCCATGCCC;

added: 2024-10-14"

CDS 3277..3294

/codon_start=1

/label=GSGSGS-Linker

/note="This feature has 2 segments:

1: 3277 .. 3290 / #993366

2: 3291 .. 3294 / #993366"

/translation="GSGSGS"

primer_bind 3291..3330

/label=for (2)

/note="color: black; sequence:

GGCCGAAGACTACAGCCGCGAGGCCGTGATCGCCGAGGTGAGCACCCAGCTG;

added: 2024-10-14"

CDS join(3295..3552,3698..4051,4197..4370)

/codon_start=1

/label=AadA Spectinomycin resistance

/note="This feature has 3 segments:

1: 3295 .. 3552 / #ff0000

2: 3698 .. 4051 / #ff0000

3: 4197 .. 4370 / #ff0000"

/translation="REAVIAEVSTQLSEVVGVIERHLEPTLLAVHLYGSAVDGGLKPHS

DIDLLVTVTVRLDETTRRALINDLLETSASPGESEILRAVEVTIVVHDDIIPWRYPAKR

ELQFGEWQRNDILAGIFEPATIDIDLAILLTKAREHSVALVGPAAEELFDPVPEQDLFE

ALNETLTLWNSPPDWAGDERNVVLTLSRIWYSAVTGKIAPKDVAADWAMERLPAQYQPV

ILEARQAYLGQEEDRLASRADQLEEFVHYVKGEITKVVGK"

intron 3553..3697

/label=rbcS2 intron 1

/note="color: #------"

intron 4052..4196

/label=rbcS2 intron 1

/note="color: #------"

primer_bind 4248..4277

/label=insert_downstream_for

/note="color: black; sequence:

GTGATCCTGGAGGCCCGCCAGGCCTACCTG; added: 2024-10-14"

3'UTR 4374..4749

/note="color: #999999"

misc_feature 4374..4424

/label=HA downstream

/note="color: #a6acb3"

primer_bind complement(4537..4566)

/label=Ctrl_3'UTR_rev

/note="color: black; sequence:

GAGAAAGAGGCCAAAATCAACGGAGGATCG; added: 2024-10-13"

primer_bind complement(4557..4594)

/label=IVD_rev

/note="color: black; sequence:

ACGCGTACTAGTCGCTTCAAATACGCCCAGCCCGCCCATGGAGAAAGAGG; added:

2024-10-13"

ORIGIN

1 tacgcaaacc gcacccgttc caccgtccta cgccgatccc gtcaagtccc gtcctagcgc

61 cattggtgga ttggtggacc gaacttcgga gtcccctgca cgatggtagt accgcactgt

121 ctcagtgtgt acaaatgatg atgaacccag tgccccaggg gagtggtgaa ctacgcagcc

181 cacgtcaagc aagccgcgac cgtcggcaca acccggatcg ccgcatgcgc cggcgcacgg

241 gtctatacat tcgacgcgag ccaggtaaaa ctcttccaca tacctcttag aggcgacacg

301 gcgccagaaa cgacgaaaaa ctggacaaac ggcaggaaca ttgtctgttt cctagcaaca

361 ccgcgagagc ggcccagatg ccccgcctgc cgtcctatga tacttcgtga cagatgaagg

421 taattggcat gctttgcgcg ccagccgggg ccgccgcgac gggggcgtat attagttgtg

481 tcacgccacg gtttgaactc gcccgcgtgg ccgagctcgt tagttttgat aaaacccagc

541 cttaatagcg tcgcgaacgt cctgagaatg caaagtgact atcgtgcgcg tgcacccgtg

601 ccgcatcctc actctgcgtg caagcccggc ttcccgggcg cgccagaagg agcgcagcca

661 aaccaggatg atgtttgatg gggtatttga gcacttgcaa cccttatccg gaagccccct

721 ggcccacaaa ggctaggcgc caatgcaagc agttcgcatg cagcccctgg agcggtgccc

781 tcctgataaa ccggccaggg ggcctatgtt ctttactttt ttacaagaga agtcactcaa

841 catcttaaaa tggccgccgt cattgccaag tcctccgtct ccgcggccgt ggcccgcccg

901 gcccgctcca gcgtgcgccc catggccgcg ctgaagcccg ccgtcaaggc cgcccccgtg

961 gctgccccgg ctcaggccaa ccagatgatg gtctggaccc cggtcaacaa caagtgagtc

1021 gacgagcaag cccggcggat caggcagcgt gcttgcagat ttgacttgca acgcccgcat

1081 tgtgtcgacg aaggcttttg gctcctctgt cgctgtctca agcagcatct aaccctgcgt

1141 cgccgtttcc atttgcagga tgttcgagac cttctcctac ctgccccccc tgagcgacga

1201 gcagatcgcc gcccaggtcg actacattgt cgccaacggt gagcttgcgg ggttgcgagc

1261 aacactccag caacgaacag tgcccaagtc aggaatctgc agtcagcctg ggctttcggc

1321 ggctttttct tgggcaaaca gcttgcactc atgccagcgc ggcttgtcca gcctcacttg

1381 agctttccag ctgctaccag ccgggctata cgacagcgac agagccatag cgtggaatca

1441 cttatttggg ttgccgaagt agcggtcgga gcgtgagttc ttggtcaagc cgccccttat

1501 ccggttcctg tccgtgtctt tgtccctcgt tcacccttcg cggcaccctt catccccttg

1561 cttgcaggct ggatcccctg cctggagttc gctgagtcgg acaaggccta cgtgtccaac

1621 gagtcggcca tccgcttcgg cagcgtgtct tgcgtaagtc tggcgagagc ccgacgggtc

1681 cactgtggca ctgggttagc ttttggcaca cgggtccact gtggcactgg ttagcttggc

1741 accgggacag cgcctatctc accgcgggga actgacgcat acccctgctc gtgcttcagc

1801 acggaaaagc aaggggccca attccatctt tggtggttct gtgcgctggt gactgaacct

1861 cttctccctc ccatttcccg tgcgcccgca gctgtactac gacaaccgct actggaccat

1921 gtggaagctg cccatgttcg gctgccgcga ccccatgcag gtgctgcgcg agatcgtcgc

1981 ctgcaccaag gccttccccg atgcctacgt gcgcctggtg gccttcgaca accagaagca

2041 ggtgcagatc atgggcttcc tggtccagcg ccccaagtct gcccgcgact ggcagcccgc

2101 caacaagcgc tccgtgggca gcggcagcgg cagcctgctg gccatccacc ccaccgaggc

2161 ccgccacaag cagaagatcg tggcccccgt gaagcagacc ctgaacttcg acctgctgaa

2221 gctggccggc gacgtggaga gcaaccccgg ccccggcagc ggcagcggca gcgtgagcaa

2281 gggcgaggag ctgttcaccg gcgtggtgcc catcctggtg gagctggacg gcgacgtgaa

2341 cggccacaag ttcagcgtga gcggcgaggg cgagggcgac gccacctacg gcaagctgac

2401 cctgaagctg atctgcacca ccggcaagct gcccgtgccc tggcccaccc tggtgaccac

2461 cctgggctac ggtgagtcga cgagcaagcc cggcggatca ggcagcgtgc ttgcagattt

2521 gacttgcaac gcccgcattg tgtcgacgaa ggcttttggc tcctctgtcg ctgtctcaag

2581 cagcatctaa ccctgcgtcg ccgtttccat ttgcaggcct gcagtgcttc gcccgctacc

2641 ccgaccacat gaagcagcac gacttcttca agagcgccat gcccgagggc tacgtgcagg

2701 agcgcaccat cttcttcaag gacgacggta actacaagac ccgcgccgag gtgaagttcg

2761 agggcgacac cctggtgaac cgcatcgagc tgaagggcat cgacttcaag gaggacggca

2821 acatcctggg ccacaagctg gagtacaact acaacagcca caacgtgtac atcaccgccg

2881 acaagcagaa gaacggcatc aaggccaact tcaagatccg ccacaacatc gaggtgagtc

2941 gacgagcaag cccggcggat caggcagcgt gcttgcagat ttgacttgca acgcccgcat

3001 tgtgtcgacg aaggcttttg gctcctctgt cgctgtctca agcagcatct aaccctgcgt

3061 cgccgtttcc atttgcagga cggcggcgtg cagctggccg accactacca gcagaacacc

3121 cccatcggcg acggccccgt gctgctgccc gacaaccact acctgagcta ccagagcaag

3181 ctgagcaagg accccaacga gaagcgcgac cacatggtgc tgctggagtt cgtgaccgcc

3241 gccggcatca ccctgggcat ggacgagctg tacaagggca gcggcagcgg cagccgcgag

3301 gccgtgatcg ccgaggtgag cacccagctg agcgaggtgg tgggcgtgat cgagcgccac

3361 ctggagccca ccctgctggc cgtgcacctg tacggcagcg ccgtggacgg cggcctgaag

3421 ccccacagcg acatcgacct gctggtgacc gtgaccgtgc gcctggacga gacgacccgc

3481 cgcgccctga tcaacgacct gctggagacg agcgccagcc ccggcgagag cgagatcctg

3541 cgcgccgtgg aggtgagtcg acgagcaagc ccggcggatc aggcagcgtg cttgcagatt

3601 tgacttgcaa cgcccgcatt gtgtcgacga aggcttttgg ctcctctgtc gctgtctcaa

3661 gcagcatcta accctgcgtc gccgtttcca tttgcaggtg accatcgtgg tgcacgacga

3721 catcatcccc tggcgctacc ccgccaagcg cgagctgcag ttcggcgagt ggcagcgcaa

3781 cgacatcctg gccggcatct tcgagcccgc caccatcgac atcgacctgg ccatcctgct

3841 gaccaaggcc cgcgagcaca gcgtggccct ggtgggcccc gccgccgagg agctgttcga

3901 ccccgtgccc gagcaggacc tgttcgaggc cctgaacgag acgctgaccc tgtggaacag

3961 cccccccgac tgggccggcg acgagcgcaa cgtggtgctg accctgagcc gcatctggta

4021 cagcgccgtg accggcaaga tcgcccccaa ggtgagtcga cgagcaagcc cggcggatca

4081 ggcagcgtgc ttgcagattt gacttgcaac gcccgcattg tgtcgacgaa ggcttttggc

4141 tcctctgtcg ctgtctcaag cagcatctaa ccctgcgtcg ccgtttccat ttgcaggacg

4201 tggccgccga ctgggccatg gagcgcctgc ccgcccagta ccagcccgtg atcctggagg

4261 cccgccaggc ctacctgggc caggaggagg accgcctggc cagccgcgcc gaccagctgg

4321 aggagttcgt gcactacgtg aagggcgaga tcaccaaggt ggtgggcaag taaatggagg

4381 cgctcgttga tctgagcctt gccccctgac gaacggcggt ggatggaaga tactgctctc

4441 aagtgctgaa gcggtagctt agctccccgt ttcgtgctga tcagtctttt tcaacacgta

4501 aaaagcggag gagttttgca attttgttgg ttgtaacgat cctccgttga ttttggcctc

4561 tttctccatg ggcgggctgg gcgtatttga agcgcttttg gaaaagttgc tgcggggttc

4621 atcagctgaa ggggactcgg ttcgcagatc agttacacac taaagaacgg cgggtagcaa

4681 caccagcaaa cgtgacgaaa cggaaccgtg cagcaaaggt ggagacagca tttgcagtaa

4741 cctgcagtga cgaacatgag tcagttgttc ccggtgcccc tttgcttaat ctgcatagaa

4801 tgatagccag ctacctagtg ccaacgtcga tagggggcga gattgggtgt acaaaacgta

4861 cattggtgag taaaggcctt acttggggtc ctccgcgctg gcgctggcgc gtgttccgca

4921 cctgttctaa acgatgcaaa cctttgattc tgccccgctg cccacttcct gcagctccca

4981 accccgcctc gtatgatgcc gttccggcat tgtgtccttg atgaccgtgc tgtatggtac

5041 aagctgtgcc catgactgca gcttctacgc tgcagtgcat cacgcctcct gtccctccct

5101 ccctccctta catgtcgtgc tgggcaccgg tggcgctggt gttctccagg ttggtttcgg

5161 gcgcatcctt tctggtagtc ccaacgccag cccggccggc gtcatccagc ccagccatcc

5221 caataccgca gccagcttcc gtcagccag

//

> edited RPL10A_YFP_aadA_Cre02.g101350

LOCUS Exported 6437 bp ds-DNA linear UNA 14-OCT-2024

DEFINITION natural linear DNA

ACCESSION .

VERSION .

KEYWORDS .

SOURCE natural DNA sequence

ORGANISM unspecified

REFERENCE 1 (bases 1 to 6437)

AUTHORS Thomas Baier

TITLE Direct Submission

JOURNAL Exported Monday, Oct 14, 2024 from SnapGene 5.0.8

https://www.snapgene.com

FEATURES Location/Qualifiers

source 1..6437

/organism="unspecified"

/mol_type="genomic DNA"

5'UTR 501..640

CDS join(641..683,924..1187,1376..1500,2453..2662)

/codon_start=1

/label=RPL10 CDS

/translation="MSKISNDVLRESVSALVEGAKTKPRKFQETVELQIGLKNYDPQKD

KRFSGSVRLPFVPRPRMRVCVLGDVKHCEQAGAIGVDAKGVEDLKKLNKNKKLVKKLAQ

AYHAFLASDSVIKQIPRLLGPGLNKAGKFPAPINKNLEEMVLDTKCSIKFQLKKVLCMG

VAVANVGMTEGEIRTNIMYAINFLVSLLKKNWQNVRCLYIKSTMGKPIRIY"

primer_bind 663..682

/label=RTqPCR_for

intron 684..923

/label=RPL10i1

primer_bind complement(1069..1088)

/label=RTqPCR_rev

intron 1188..1375

/label=RPL10i2

intron 1501..2452

/label=RPL10i3

primer_bind 2428..2452

/label=Ctrl_CDS_for

primer_bind 2428..2452

/label=IVD_for

misc_feature 2570..2637

/label=HA upstream

misc_feature 2578..2580

/label=PAM

misc_feature 2581..2600

/label=sgRNA1

misc_feature 2620..2639

/label=sgRNA3

misc_feature 2621..2640

/label=sgRNA 2

misc_feature 2640..2642

/label=PAM

misc_feature 2641..2643

/label=PAM

CDS 2663..2680

/codon_start=1

/label=GSGSGS-Linker

/translation="GSGSGS"

CDS 2681..2798

/codon_start=1

/product="unnamed_input_seq"

/label=ext2A

/label=unnamed_input_seq__CDS

/translation="LLAIHPTEARHKQKIVAPVKQTLNFDLLKLAGDVESNPG"

CDS 2801..2818

/codon_start=1

/label=GSGSGS-Linker

/translation="GSGSGS"

CDS join(2819..3017,3163..3479,3625..3822)

/codon_start=1

/label=mVenus

/translation="VSKGEELFTGVVPILVELDGDVNGHKFSVSGEGEGDATYGKLTLK

LICTTGKLPVPWPTLVTTLGYGLQCFARYPDHMKQHDFFKSAMPEGYVQERTIFFKDDG

NYKTRAEVKFEGDTLVNRIELKGIDFKEDGNILGHKLEYNYNSHNVYITADKQKNGIKA

NFKIRHNIEDGGVQLADHYQQNTPIGDGPVLLPDNHYLSYQSKLSKDPNEKRDHMVLLE

FVTAAGITLGMDELYK"

primer_bind complement(2833..2852)

/label=insert_upstream_rev

intron 3018..3162

/label=rbcS2 intron 1

intron 3480..3624

/label=rbcS2 intron 1

primer_bind complement(3801..3840)

/label=rev

CDS 3823..3840

/codon_start=1

/label=GSGSGS-Linker

/translation="GSGSGS"

primer_bind 3837..3876

/label=for (2)

CDS join(3841..4098,4244..4597,4743..4916)

/codon_start=1

/label=AadA Spectinomycin resistance

/translation="REAVIAEVSTQLSEVVGVIERHLEPTLLAVHLYGSAVDGGLKPHS

DIDLLVTVTVRLDETTRRALINDLLETSASPGESEILRAVEVTIVVHDDIIPWRYPAKR

ELQFGEWQRNDILAGIFEPATIDIDLAILLTKAREHSVALVGPAAEELFDPVPEQDLFE

ALNETLTLWNSPPDWAGDERNVVLTLSRIWYSAVTGKIAPKDVAADWAMERLPAQYQPV

ILEARQAYLGQEEDRLASRADQLEEFVHYVKGEITKVVGK"

intron 4099..4243

/label=rbcS2 intron 1

intron 4598..4742

/label=rbcS2 intron 1

primer_bind 4794..4823

/label=insert_downstream_for

3'UTR 4920..5937

misc_feature 4920..4979

/label=HA downstream

primer_bind complement(5071..5100)

/label=Ctrl_3'UTR_rev

primer_bind complement(5239..5263)

/label=IVD_rev

ORIGIN

1 gcacaaatac acacactacg ggggcggcat gcaactgtgg taaacagggg ggtgctctta

61 aagacacact ctccgggtgc tcgtggagcg tgaaggaaga ttgctgtaga cgctagagcg

121 ggctcgcggc attccgagtc agctgcttag tatagcgctt agtgcataag cgaagcagcg

181 gtggtgcatt caggaacatg tgtgacgcca ggtctgtaac aaaggactac agcatctcgg

241 cgactcggcc cgcaaaccct gctacaccgg cgacggggca gtgggggaca ttccgttgtt

301 ccgtgtagtg caggctatga ttgtgaaatt acaagaaggg acagtccaac caagctaggg

361 tgcccaggct atgcgcagca agttcgcatg ccaggcgcga gggtggcggg aacgggcggg

421 tcagggtgac gggttgaggg caaggcacgg tacccgccag ccaactgggc cttactttca

481 tcatagggaa agcataaatc ataacagtgt agtttatatt atgcatgatg tcttccgcag

541 aagaggcacc gtgatgccca ccgcccccat gcatcaattg tgagggtcaa gagcgcccgc

601 ggacccctgg acattccttt tccttgggtg aagcacgaaa atgagcaaga tctcgaacga

661 cgttctccgc gagagtgtct ccggtgagct ctttttctga gtagaaactt tcctttctgg

721 gcttgcttct tcggcgactt gacgcgtcta gcttagctcg ctcgattttc gcttctacgt

781 agtgttaacg atagcttatg aagcaagttg acaattaagg cacagggcag gagtcgccgc

841 cggcacaagt cgcgccgggt tttacgctcg tcgcacgcgc tgctgacgct ctgtaatttt

901 atgttgcctc ccgtttattg cagccctggt ggagggggct aagaccaagc cccggaagtt

961 ccaggagacc gtggagctgc agattggtct gaagaactac gacccccaga aggacaagcg

1021 tttcagcggc tccgtccgcc tgcccttcgt gcctcgcccc cgcatgcgcg tgtgcgtgct

1081 gggtgatgtg aagcactgcg agcaggccgg cgccattggc gtcgacgcca agggtgtgga

1141 ggacctgaag aagctgaaca agaacaagaa gctggtcaag aagctgggtg agttggggca

1201 tcgcttgagg gaggcgcgaa agtggcggcc gagaatggct gggttgccag gcggttccgt

1261 gcgacatact caccgcaaca ttgcttttaa taatacggtg gtatcagatt ttgatgagcg

1321 cgcggattgc cagacgtgag ctaacccgac cccgacggcc ttcctcgccc cacagcccag

1381 gcgtaccacg ccttcctggc ttcggactcc gtcatcaagc agattccccg tctgctgggc

1441 cccggtctga acaaggccgg caagttccct gctcctatca acaagaacct ggaggagatg

1501 gtgagcgaca aacgcaacgc ccttgggttg gcagggatgg gacgggggca ggcagggtgg

1561 gctgctcact gactgcagtg gtaagggttg aattatggca cagcttgaca taactgcgcg

1621 ctggaaagca ttggagctgg agcggagcgg agcggacggg agcctggaag ctcggaggct

1681 ggcgcagagc gtattggcgg ccggtgggac aggggacggg tctggcgcac agggcagcag

1741 gctgcggggc cgacaggagg caggagcagg cgcttgcctt aatatgcgaa agccaggcga

1801 acccagctgg cgagaataga tggaccgcag ccctgggcgg cctcgggcag cggcgcagaa

1861 gctggcggca gtttgccagc ttctacaccg ctggactggg cagcaggggc tcactgcagg

1921 ctggcgaggg cagcggccat gttgcgtcat tagcgcaatg gattcaatgc acgccgtcag

1981 cggttatgcc atggcgtgat tggacggacc acctcgcgca gcctcagccg gctgctgcgg

2041 ctgcaagtgg gtgcgcttgc agcgagccaa ggccagcggg cggctgggca cagcaggggg

2101 acctctgctg ctaagcgcca cagcgtggat cctctactgc gcacggcgcg gacggctcag

2161 tgcaagaaga cggagggact cagcgacagc atgatgatca gccgctgcag cggcggcact

2221 cgctcgggtg ggctggccaa gggctggaac cgcggtctgg aggaaagccg ctcggcagca

2281 gcagcagcaa cagcctggct tccgggctcc cccggttggc ccaatgagcg caggccagcg

2341 cgtggatgcg caagcgccag acttgaacac caaatattgt agcacaacca caggtccctg

2401 accgccctgc ccgccctgcc gtgccgcgct tgccttgctc ttccctgcac aggtgctgga

2461 caccaagtgc agcatcaagt tccagctgaa gaaggtgctg tgcatgggcg tggccgtggc

2521 caacgtgggc atgaccgagg gcgagatccg taccaacatc atgtacgcca tcaacttcct

2581 ggtgtcgctg ctcaagaaga actggcagaa cgtgcgctgc ctgtacatca agagcaccat

2641 gggcaagccc atccgcatct acggcagcgg cagcggcagc ctgctggcca tccaccccac

2701 cgaggcccgc cacaagcaga agatcgtggc ccccgtgaag cagaccctga acttcgacct

2761 gctgaagctg gccggcgacg tggagagcaa ccccggcccc ggcagcggca gcggcagcgt

2821 gagcaagggc gaggagctgt tcaccggcgt ggtgcccatc ctggtggagc tggacggcga

2881 cgtgaacggc cacaagttca gcgtgagcgg cgagggcgag ggcgacgcca cctacggcaa

2941 gctgaccctg aagctgatct gcaccaccgg caagctgccc gtgccctggc ccaccctggt

3001 gaccaccctg ggctacggtg agtcgacgag caagcccggc ggatcaggca gcgtgcttgc

3061 agatttgact tgcaacgccc gcattgtgtc gacgaaggct tttggctcct ctgtcgctgt

3121 ctcaagcagc atctaaccct gcgtcgccgt ttccatttgc aggcctgcag tgcttcgccc

3181 gctaccccga ccacatgaag cagcacgact tcttcaagag cgccatgccc gagggctacg

3241 tgcaggagcg caccatcttc ttcaaggacg acggtaacta caagacccgc gccgaggtga

3301 agttcgaggg cgacaccctg gtgaaccgca tcgagctgaa gggcatcgac ttcaaggagg

3361 acggcaacat cctgggccac aagctggagt acaactacaa cagccacaac gtgtacatca

3421 ccgccgacaa gcagaagaac ggcatcaagg ccaacttcaa gatccgccac aacatcgagg

3481 tgagtcgacg agcaagcccg gcggatcagg cagcgtgctt gcagatttga cttgcaacgc

3541 ccgcattgtg tcgacgaagg cttttggctc ctctgtcgct gtctcaagca gcatctaacc

3601 ctgcgtcgcc gtttccattt gcaggacggc ggcgtgcagc tggccgacca ctaccagcag

3661 aacaccccca tcggcgacgg ccccgtgctg ctgcccgaca accactacct gagctaccag

3721 agcaagctga gcaaggaccc caacgagaag cgcgaccaca tggtgctgct ggagttcgtg

3781 accgccgccg gcatcaccct gggcatggac gagctgtaca agggcagcgg cagcggcagc

3841 cgcgaggccg tgatcgccga ggtgagcacc cagctgagcg aggtggtggg cgtgatcgag

3901 cgccacctgg agcccaccct gctggccgtg cacctgtacg gcagcgccgt ggacggcggc

3961 ctgaagcccc acagcgacat cgacctgctg gtgaccgtga ccgtgcgcct ggacgagacg

4021 acccgccgcg ccctgatcaa cgacctgctg gagacgagcg ccagccccgg cgagagcgag

4081 atcctgcgcg ccgtggaggt gagtcgacga gcaagcccgg cggatcaggc agcgtgcttg

4141 cagatttgac ttgcaacgcc cgcattgtgt cgacgaaggc ttttggctcc tctgtcgctg

4201 tctcaagcag catctaaccc tgcgtcgccg tttccatttg caggtgacca tcgtggtgca

4261 cgacgacatc atcccctggc gctaccccgc caagcgcgag ctgcagttcg gcgagtggca

4321 gcgcaacgac atcctggccg gcatcttcga gcccgccacc atcgacatcg acctggccat

4381 cctgctgacc aaggcccgcg agcacagcgt ggccctggtg ggccccgccg ccgaggagct

4441 gttcgacccc gtgcccgagc aggacctgtt cgaggccctg aacgagacgc tgaccctgtg

4501 gaacagcccc cccgactggg ccggcgacga gcgcaacgtg gtgctgaccc tgagccgcat

4561 ctggtacagc gccgtgaccg gcaagatcgc ccccaaggtg agtcgacgag caagcccggc

4621 ggatcaggca gcgtgcttgc agatttgact tgcaacgccc gcattgtgtc gacgaaggct

4681 tttggctcct ctgtcgctgt ctcaagcagc atctaaccct gcgtcgccgt ttccatttgc

4741 aggacgtggc cgccgactgg gccatggagc gcctgcccgc ccagtaccag cccgtgatcc

4801 tggaggcccg ccaggcctac ctgggccagg aggaggaccg cctggccagc cgcgccgacc

4861 agctggagga gttcgtgcac tacgtgaagg gcgagatcac caaggtggtg ggcaagtaag

4921 caattggtgg gtgcgggccg gcggcccgtt gggggctgcg cggccgcgcc ggggttcccc

4981 gggcgtgctg gtcacacgcc cggcccggct gcggtgttgg cgcgtggctc gagttggttc

5041 ttcagttcct ggaactctcc accatggtca gggataactc gtcgcacgaa cattaccaag

5101 gaccggggcg cttttagcct gcctgccctc gtggctggcg aggcggcgct tgccggcttc

5161 cattgggagt tcacatgagt gactcatgct cgttcggcag gcttcactgc gtttggaaag

5221 gttacatgac gacaagctcc gcaccgagaa tgctttctga tcctgcgggc ctctggcttg

5281 cgcggtgtgt ttgtaacgcc ggcgtagtga tcatgcctcg tgcctcgggt tcgttctttg

5341 ggggttgttg gtggaaaaca ctgagcagcg tcagccaaat atagacgacg gcaggagccg

5401 gccctgcacc tagagcgttg ggccaggcac tgggtacgca gcccacccag attcttgaac

5461 ttgagcagca cagcatctaa cagccttgat tgacgtgtgt tgcggaacta acgcgtacgt

5521 agaccagagc ggaaggcgga gtgggcttgc aggcggcacg agagcaggca gtcagtagcg

5581 caagttgcga atccgcatgt tccctcgtaa cggaccgtga acactgtgga cggtcgcgcc

5641 catggctagc ggacgtaagg tggctgctgt attgggatgg ctgggctgcc gggctgggcg

5701 acggcggccg gccttgatgt gacagagaac cgcacatgtc atgcagtaca cggggtggcg

5761 catggctggg caccaggttg gaccacattg gccattgcaa gtggcgttgc ggtgtgtctg

5821 tgtggtgcca tcgtctggcc gggcgcccgg agcgtgaggg attcgcatca gcaaacttcc

5881 agtcagcaag caacggaaca gcgacggtac ggtacagaaa ctccggcata acggcattcg

5941 atcagccgtg cactcctgcc ctgtcgttgc aagttgttgc catctgctgg ggtatgggaa

6001 tcagctgcac cctgcgggac ttcaccaacc tgccttcaca ctgtcccacc gcacactcat

6061 gccaaaggaa atgtccttcc gcagggttgg cttgggtcag gaggggagaa gacgaacagc

6121 catcggcatg agcgcttctt gcccgcacgg agcacactat ggcaccaact ggtgcccaag

6181 aggtattgcc ctgccctcag cttgcttcta agcttcaact gtccccaagg ctacgcacac

6241 aagcgcctga cttcagcaca tcaccaccac aagagtggaa acttccaacg cctgcctgag

6301 atacacacac attcacacac tccaggacag gccacatcac gttgtcccaa gccccagtca

6361 gttgcagtgt cagtgcccac ctgcctacct gccgacctcc gatatatact tgcctgactg

6421 cccggccttg ctgctcg

//

> edited LHCBM1_CnVs_aadA_Cre01.g066917

LOCUS Exported 5562 bp ds-DNA linear UNA 14-OCT-2024

DEFINITION natural linear DNA

ACCESSION .

VERSION .

KEYWORDS .

SOURCE natural DNA sequence

ORGANISM unspecified

REFERENCE 1 (bases 1 to 5562)

AUTHORS Thomas Baier

TITLE Direct Submission

JOURNAL Exported Monday, Oct 14, 2024 from SnapGene 5.0.8

https://www.snapgene.com

FEATURES Location/Qualifiers

source 1..5562

/organism="unspecified"

/mol_type="genomic DNA"

5'UTR 1..25

CDS join(26..141,379..461,715..1046,1195..1364,1543..1609)

/codon_start=1

/label=LHCBM1

/translation="MAFALAKSSARAAVSRRSTVKVEARRTVKPASKASTPDSFWYGPE

RPLFLGAFTGEPPSYLTGEFPGDYGWDTAGLSADPETFKRYRELELIHARWAMLGALGC

IFPELLGSYGVPFGEAVWFKAGAQIFQEGGLDYLGNPNLVHAQSILAILGTQVLLMGAI

EGYRVNGGPLGEGLDKLYPGGSFDPLGLADDPDTFAELKVKEIKNGRLAMFSMFGFFVQ

AIVTGKGPLQNLSDHLANPGTNNAFAYATKFTPQ"

primer_bind 77..96

/label=RTqPCR_for

primer_bind complement(776..795)

/label=RTqPCR_rev

primer_bind 1211..1238

/label=Ctrl_CDS_for

primer_bind 1211..1238

/label=IVD_for

misc_feature 1496..1542

/label=HA EcoRV

misc_feature 1537..1539

/label=PAM

misc_feature 1540..1559

/label=sgRNA1 rev

misc_feature 1590..1592

/label=PAM

misc_feature 1593..1609

/label=sgRNA2 rev

CDS 1610..1627

/codon_start=1

/label=GSGSGS-Linker

/translation="GSGSGS"

CDS 1628..1745

/codon_start=1

/product="unnamed_input_seq"

/label=ext2A

/label=unnamed_input_seq__CDS

/translation="LLAIHPTEARHKQKIVAPVKQTLNFDLLKLAGDVESNPG"

primer_bind complement(1675..1735)

/label=insert_upstream_rev (1)

CDS 1748..1765

/codon_start=1

/label=GSGSGS-Linker

/translation="GSGSGS"

CDS join(1766..1863,2009..2396,2542..2943,3089..3500,3646..4032,

4178..4254)

/codon_start=1

/product="AFN21429.1 terpene synthase Valencene synthase

[Callitropsis nootkatensis] Beekwilder 2014"

/label=AFN21429.1 terpene synthase Valencene synthase

/label=AFN21429.1 terpene synthase Valencene synthase

[Callitropsis nootkatensis] Beekwilder 2014__CDS

/translation="AEMFNGNSSNDGSSCMPVKDALRRTGNHHPNLWTDDFIQSLNSPY

SDSSYHKHREILIDEIRDMFSNGEGDEFGVLENIWFVDVVQRLGIDRHFQEEIKTALDY

IYKFWNHDSIFGDLNMVALGFRILRLNRYVASSDVFKKFKGEEGQFSGFESSDQDAKLE

MMLNLYKASELDFPDEDILKEARAFASMYLKHVIKEYGDIQESKNPLLMEIEYTFKYPW

RCRLPRLEAWNFIHIMRQQDCNISLANNLYKIPKIYMKKILELAILDFNILQSQHQHEM

KLISTWWKNSSAIQLDFFRHRHIESYFWWASPLFEPEFSTCRINCTKLSTKMFLLDDIY

DTYGTVEELKPFTTTLTRWDVSTVDNHPDYMKIAFNFSYEIYKEIASEAERKHGPFVYK

YLQSCWKSYIEAYMQEAEWIASNHIPGFDEYLMNGVKSSGMRILMIHALILMDTPLSDE

ILEQLDIPSSKSQALLSLITRLVDDVKDFEDEQAHGEMASSIECYMKDNHGSTREDALN

YLKIRIESCVQELNKELLEPSNMHGSFRNLYLNVGMRVIFFMLNDGDLFTHSNRKEIQD

AITKFFVEPIIP"

intron 1864..2008

/label=intron

intron 2397..2541

/label=intron

intron 2944..3088

/label=intron

intron 3501..3645

/label=intron

intron 4033..4177

/label=intron

CDS 4255..4272

/codon_start=1

/label=GSGSGS-Linker

/translation="GSGSGS"

CDS join(4273..4530,4676..5029,5175..5348)

/codon_start=1

/label=AadA Spectinomycin resistance

/translation="REAVIAEVSTQLSEVVGVIERHLEPTLLAVHLYGSAVDGGLKPHS

DIDLLVTVTVRLDETTRRALINDLLETSASPGESEILRAVEVTIVVHDDIIPWRYPAKR

ELQFGEWQRNDILAGIFEPATIDIDLAILLTKAREHSVALVGPAAEELFDPVPEQDLFE

ALNETLTLWNSPPDWAGDERNVVLTLSRIWYSAVTGKIAPKDVAADWAMERLPAQYQPV

ILEARQAYLGQEEDRLASRADQLEEFVHYVKGEITKVVGK"

intron 4531..4675

/label=rbcS2 intron 1

intron 5030..5174

/label=rbcS2 intron 1

primer_bind 5226..5255

/label=insert_downstream_for

3'UTR 5352..5562

misc_feature 5352..5389

/label=HA2 EcoRV

primer_bind complement(5519..5562)

/label=Ctrl_3'UTR_rev

primer_bind complement(5519..5562)

/label=IVD_rev

ORIGIN

1 acagaagtta ctcccaccag tcaaaatggc cttcgccctt gccaagtcct ccgctcgcgc

61 cgcggtgtct cgccgctcga ccgtcaaggt cgaggcgcgc cgcaccgtga agcccgcctc

121 caaggcgtcc accccggaca ggtgtgtagc tcgccaatga agtgctctgg ggaagatttg

181 cagggggaat gacagggcac gactgccgga actggccact ccggcatggg gatcctcttt

241 ccctgacttg cgcttcctgt ctggtgagct gccaccagtg gcaaagaata cagctccatg

301 tctacaatgc ggcctgccga tttcactgta tcaagctctt gacgtctgaa ccctttcgcg

361 atggcccttt gcttgcagct tctggtatgg ccctgagcgc cccctgttcc tgggcgcctt

421 cactggcgag cccccgagct acctgactgg cgagttcccc ggtaagtctt tctgtgtcgc

481 ggggttctgg gcgttcgcat gcgcaacagt gtcgcacggt cgctcttgca gcacagtcac

541 tacagatagt ccaagtccga cgcatggcga tcgggcaact gcgatttgca catgcggcaa

601 gggatctcta gctcgggctg gcgaagcctt caggacatgg agcgctgtcc agcagctggt

661 tggtgatgct ctatcctaaa ttgcccctcc cacacaccct tacttgcttt ccaggtgact

721 acggctggga caccgccggt ctgtccgctg acccggagac cttcaagcgc taccgcgagc

781 tggagctgat ccacgcccgc tgggccatgc tcggcgctct gggctgcatc ttccccgagc

841 tgctgggctc ctacggcgtg cccttcggcg aggccgtgtg gttcaaggct ggtgctcaga

901 tcttccagga gggcggtctg gactacctgg gcaaccccaa cctggtgcac gcccagtcca

961 tcctggccat cctgggcacc caggtgctgc tgatgggcgc cattgagggc taccgcgtca

1021 acggcggccc cctgggcgag ggcctggtga gtgcggttcg ctgtgaaggg aagcttccct

1081 caaaacacat gggacagttt cggtttgtgt tgtctggttg cgtcagtagc aatgcacggt

1141 tcgtggacgt tgtgcatggc atcattgccc acccttgctc cctccgcgtt gcaggacaag

1201 ctgtaccccg gtggctcgtt cgaccccctg ggcctggctg acgaccccga caccttcgct

1261 gagctgaagg tgaaggagat caagaacggc cgcctggcca tgttctccat gttcggcttc

1321 ttcgttcagg ccatcgtgac cggcaagggc cccctgcaga acctgtgagt agggatgggg

1381 gcctagggtg acataacatg agtcgcggtg tgtgcagcac gctgcttgcc ataaatgccc

1441 agccgcgcta acctacgcgg attaaacatt aattcgagtt gatacttgca ctcccatctt

1501 agtttacccg gtgatgttat gctcctcgtg cccgctccac aggtccgacc acctggccaa

1561 ccccggcacc aacaacgcct tcgcctacgc caccaagttc accccccagg gcagcggcag

1621 cggcagcctg ctggccatcc accccaccga ggcccgccac aagcagaaga tcgtggcccc

1681 cgtgaagcag accctgaact tcgacctgct gaagctggcc ggcgacgtgg agagcaaccc

1741 cggccccggc agcggcagcg gcagcgccga gatgttcaac ggcaacagca gcaacgacgg

1801 cagcagctgc atgcccgtga aggacgccct gcgccgcacc ggcaaccacc accccaacct

1861 gtggtgagtc gacgagcaag cccggcggat caggcagcgt gcttgcagat ttgacttgca

1921 acgcccgcat tgtgtcgacg aaggcttttg gctcctctgt cgctgtctca agcagcatct

1981 aaccctgcgt cgccgtttcc atttgcagga ccgacgactt catccagagc ctgaacagcc

2041 cctacagcga cagcagctac cacaagcacc gcgagatcct gatcgacgag atccgcgaca

2101 tgttcagcaa cggcgagggc gacgagttcg gcgtgctgga gaacatctgg ttcgtggacg

2161 tggtgcagcg cctgggcatc gaccgccact tccaggagga gatcaagacc gccctggact

2221 acatctacaa gttctggaac cacgacagca tcttcggcga cctgaacatg gtggccctgg

2281 gcttccgcat cctgcgcctg aaccgctacg tggccagcag cgacgtgttc aagaagttca

2341 agggcgagga gggccagttc agcggcttcg agagcagcga ccaggacgcc aagctggtga

2401 gtcgacgagc aagcccggcg gatcaggcag cgtgcttgca gatttgactt gcaacgcccg

2461 cattgtgtcg acgaaggctt ttggctcctc tgtcgctgtc tcaagcagca tctaaccctg

2521 cgtcgccgtt tccatttgca ggagatgatg ctgaacctgt acaaggccag cgagctggac

2581 ttccccgacg aggacatcct gaaggaggcc cgcgccttcg ccagcatgta cctgaagcac

2641 gtgatcaagg agtacggcga catccaggag agcaagaacc ccctgctgat ggagatcgag

2701 tacaccttca agtacccctg gcgctgccgc ctgccccgcc tggaggcctg gaacttcatc

2761 cacatcatgc gccagcagga ctgcaacatc agcctggcca acaacctgta caagatcccc

2821 aagatttaca tgaagaagat cctggagctg gccatcctgg acttcaacat cctgcagagc

2881 cagcaccagc acgagatgaa gctgatcagc acctggtgga agaacagcag cgccatccag

2941 ctggtgagtc gacgagcaag cccggcggat caggcagcgt gcttgcagat ttgacttgca

3001 acgcccgcat tgtgtcgacg aaggcttttg gctcctctgt cgctgtctca agcagcatct

3061 aaccctgcgt cgccgtttcc atttgcagga cttcttccgc caccgccaca tcgagagcta

3121 cttctggtgg gccagccccc tgttcgagcc cgagttcagc acctgccgca tcaactgcac

3181 caagctgagc accaagatgt tcctgctgga cgacatctac gacacctacg gcaccgtgga

3241 ggagctgaag cccttcacca ccaccctgac ccgctgggac gtgagcaccg tggacaacca

3301 ccccgactac atgaagatcg ccttcaactt cagctacgag atttacaagg agatcgccag

3361 cgaggccgag cgcaagcacg gccccttcgt gtacaagtac ctgcagagct gctggaagag

3421 ctacatcgag gcctacatgc aggaggccga gtggatcgcc agcaaccaca tccccggctt

3481 cgacgagtac ctgatgaacg gtgagtcgac gagcaagccc ggcggatcag gcagcgtgct

3541 tgcagatttg acttgcaacg cccgcattgt gtcgacgaag gcttttggct cctctgtcgc

3601 tgtctcaagc agcatctaac cctgcgtcgc cgtttccatt tgcaggcgtg aagagcagcg

3661 gcatgcgcat cctgatgatc cacgccctga tcctgatgga cacccccctg agcgacgaga

3721 tcctggagca gctggacatc cccagcagca agagccaggc cctgctgagc ctgatcaccc

3781 gcctggtgga cgacgtgaag gacttcgagg acgagcaggc ccacggcgag atggccagca

3841 gcatcgagtg ctacatgaag gacaaccacg gcagcacccg cgaggacgcc ctgaactacc

3901 tgaagatccg catcgagagc tgcgtgcagg agctgaacaa ggagctgctg gagcccagca

3961 acatgcacgg cagcttccgc aacctgtacc tgaacgtggg catgcgcgtg atcttcttca

4021 tgctgaacga cggtgagtcg acgagcaagc ccggcggatc aggcagcgtg cttgcagatt

4081 tgacttgcaa cgcccgcatt gtgtcgacga aggcttttgg ctcctctgtc gctgtctcaa

4141 gcagcatcta accctgcgtc gccgtttcca tttgcaggcg acctgttcac ccacagcaac

4201 cgcaaggaga tccaggacgc catcaccaag ttcttcgtgg agcccatcat ccccggcagc

4261 ggcagcggca gccgcgaggc cgtgatcgcc gaggtgagca cccagctgag cgaggtggtg

4321 ggcgtgatcg agcgccacct ggagcccacc ctgctggccg tgcacctgta cggcagcgcc

4381 gtggacggcg gcctgaagcc ccacagcgac atcgacctgc tggtgaccgt gaccgtgcgc

4441 ctggacgaga cgacccgccg cgccctgatc aacgacctgc tggagacgag cgccagcccc

4501 ggcgagagcg agatcctgcg cgccgtggag gtgagtcgac gagcaagccc ggcggatcag

4561 gcagcgtgct tgcagatttg acttgcaacg cccgcattgt gtcgacgaag gcttttggct

4621 cctctgtcgc tgtctcaagc agcatctaac cctgcgtcgc cgtttccatt tgcaggtgac

4681 catcgtggtg cacgacgaca tcatcccctg gcgctacccc gccaagcgcg agctgcagtt

4741 cggcgagtgg cagcgcaacg acatcctggc cggcatcttc gagcccgcca ccatcgacat

4801 cgacctggcc atcctgctga ccaaggcccg cgagcacagc gtggccctgg tgggccccgc

4861 cgccgaggag ctgttcgacc ccgtgcccga gcaggacctg ttcgaggccc tgaacgagac

4921 gctgaccctg tggaacagcc cccccgactg ggccggcgac gagcgcaacg tggtgctgac

4981 cctgagccgc atctggtaca gcgccgtgac cggcaagatc gcccccaagg tgagtcgacg

5041 agcaagcccg gcggatcagg cagcgtgctt gcagatttga cttgcaacgc ccgcattgtg

5101 tcgacgaagg cttttggctc ctctgtcgct gtctcaagca gcatctaacc ctgcgtcgcc

5161 gtttccattt gcaggacgtg gccgccgact gggccatgga gcgcctgccc gcccagtacc

5221 agcccgtgat cctggaggcc cgccaggcct acctgggcca ggaggaggac cgcctggcca

5281 gccgcgccga ccagctggag gagttcgtgc actacgtgaa gggcgagatc accaaggtgg

5341 tgggcaagta aatgccctgg cggcacagtt ttgatgtacc aatagggatg caggtctgag

5401 cggtttattt gggtcgtctt gtgtggtctg gtggagcttg agttgtttgg gagcggtggg

5461 ttttgtgtgc ggtctggccg tgcagcaggc aaggtcccga caggcgcagg agcggctagc

5521 tgcgctggga cttgtcaacg tttgtaaatt ttgagagaag ac

//

> edited RBCS2_CnVs_aadA_Cre02.g120150

LOCUS Exported 6734 bp ds-DNA linear UNA 14-OCT-2024

DEFINITION natural linear DNA

ACCESSION .

VERSION .

KEYWORDS .

SOURCE natural DNA sequence

ORGANISM unspecified

REFERENCE 1 (bases 1 to 6734)

AUTHORS Thomas Baier

TITLE Direct Submission

JOURNAL Exported Monday, Oct 14, 2024 from SnapGene 5.0.8

https://www.snapgene.com

FEATURES Location/Qualifiers

source 1..6734

/organism="unspecified"

/mol_type="genomic DNA"

5'UTR 501..849

CDS join(850..1013,1159..1238,1568..1653,1892..2116)

/codon_start=1

/label=rbcS2 CDS

/translation="MAAVIAKSSVSAAVARPARSSVRPMAALKPAVKAAPVAAPAQANQ

MMVWTPVNNKMFETFSYLPPLSDEQIAAQVDYIVANGWIPCLEFAESDKAYVSNESAIR

FGSVSCLYYDNRYWTMWKLPMFGCRDPMQVLREIVACTKAFPDAYVRLVAFDNQKQVQI

MGFLVQRPKSARDWQPANKRSV"

intron 1014..1158

/label=rbcS2i1

primer_bind 1213..1232

/label=RTqPCR_for

intron 1239..1567

/label=rbcS2i2

primer_bind 1630..1673

/label=IVD_for (1)

intron 1654..1891

/label=rbcS2i3

primer_bind 1904..1928

/label=Ctrl_CDS_for

primer_bind complement(1954..1973)

/label=RTqPCR_rev

misc_feature 2030..2076

/label=HA upstream

misc_feature 2059..2061

/label=PAM

misc_feature 2062..2081

/label=sgRNA3

misc_feature 2070..2089

/label=sgRNA1

misc_feature 2071..2073

/label=PAM

misc_feature 2074..2093

/label=sgRNA2

misc_feature 2083..2085

/label=PAM

misc_feature 2086..2105

/label=sgRNA4

misc_feature 2090..2092

/label=PAM

CDS 2117..2134

/codon_start=1

/label=GSGSGS-Linker

/translation="GSGSGS"

CDS 2135..2252

/codon_start=1

/product="unnamed_input_seq"

/label=ext2A

/label=unnamed_input_seq__CDS

/translation="LLAIHPTEARHKQKIVAPVKQTLNFDLLKLAGDVESNPG"

primer_bind complement(2182..2242)

/label=insert_upstream_rev (1)

CDS 2255..2272

/codon_start=1

/label=GSGSGS-Linker

/translation="GSGSGS"

CDS join(2273..2370,2516..2903,3049..3450,3596..4007,4153..4539,

4685..4761)

/codon_start=1

/product="AFN21429.1 terpene synthase Valencene synthase

[Callitropsis nootkatensis] Beekwilder 2014"

/label=AFN21429.1 terpene synthase Valencene synthase

/label=AFN21429.1 terpene synthase Valencene synthase

[Callitropsis nootkatensis] Beekwilder 2014__CDS

/translation="AEMFNGNSSNDGSSCMPVKDALRRTGNHHPNLWTDDFIQSLNSPY

SDSSYHKHREILIDEIRDMFSNGEGDEFGVLENIWFVDVVQRLGIDRHFQEEIKTALDY

IYKFWNHDSIFGDLNMVALGFRILRLNRYVASSDVFKKFKGEEGQFSGFESSDQDAKLE

MMLNLYKASELDFPDEDILKEARAFASMYLKHVIKEYGDIQESKNPLLMEIEYTFKYPW

RCRLPRLEAWNFIHIMRQQDCNISLANNLYKIPKIYMKKILELAILDFNILQSQHQHEM

KLISTWWKNSSAIQLDFFRHRHIESYFWWASPLFEPEFSTCRINCTKLSTKMFLLDDIY

DTYGTVEELKPFTTTLTRWDVSTVDNHPDYMKIAFNFSYEIYKEIASEAERKHGPFVYK

YLQSCWKSYIEAYMQEAEWIASNHIPGFDEYLMNGVKSSGMRILMIHALILMDTPLSDE

ILEQLDIPSSKSQALLSLITRLVDDVKDFEDEQAHGEMASSIECYMKDNHGSTREDALN

YLKIRIESCVQELNKELLEPSNMHGSFRNLYLNVGMRVIFFMLNDGDLFTHSNRKEIQD

AITKFFVEPIIP"

intron 2371..2515

/label=intron

intron 2904..3048

/label=intron

intron 3451..3595

/label=intron

intron 4008..4152

/label=intron

intron 4540..4684

/label=intron

CDS 4762..4779

/codon_start=1

/label=GSGSGS-Linker

/translation="GSGSGS"

CDS join(4780..5037,5183..5536,5682..5855)

/codon_start=1

/label=AadA Spectinomycin resistance

/translation="REAVIAEVSTQLSEVVGVIERHLEPTLLAVHLYGSAVDGGLKPHS

DIDLLVTVTVRLDETTRRALINDLLETSASPGESEILRAVEVTIVVHDDIIPWRYPAKR

ELQFGEWQRNDILAGIFEPATIDIDLAILLTKAREHSVALVGPAAEELFDPVPEQDLFE

ALNETLTLWNSPPDWAGDERNVVLTLSRIWYSAVTGKIAPKDVAADWAMERLPAQYQPV

ILEARQAYLGQEEDRLASRADQLEEFVHYVKGEITKVVGK"

intron 5038..5182

/label=rbcS2 intron 1

intron 5537..5681

/label=rbcS2 intron 1

primer_bind 5733..5762

/label=insert_downstream_for

3'UTR 5859..6234

misc_feature 5859..5909

/label=HA downstream

primer_bind complement(6022..6051)

/label=Ctrl_3'UTR_rev

primer_bind complement(6042..6079)

/label=IVD_rev

ORIGIN

1 tacgcaaacc gcacccgttc caccgtccta cgccgatccc gtcaagtccc gtcctagcgc

61 cattggtgga ttggtggacc gaacttcgga gtcccctgca cgatggtagt accgcactgt

121 ctcagtgtgt acaaatgatg atgaacccag tgccccaggg gagtggtgaa ctacgcagcc

181 cacgtcaagc aagccgcgac cgtcggcaca acccggatcg ccgcatgcgc cggcgcacgg

241 gtctatacat tcgacgcgag ccaggtaaaa ctcttccaca tacctcttag aggcgacacg

301 gcgccagaaa cgacgaaaaa ctggacaaac ggcaggaaca ttgtctgttt cctagcaaca

361 ccgcgagagc ggcccagatg ccccgcctgc cgtcctatga tacttcgtga cagatgaagg

421 taattggcat gctttgcgcg ccagccgggg ccgccgcgac gggggcgtat attagttgtg

481 tcacgccacg gtttgaactc gcccgcgtgg ccgagctcgt tagttttgat aaaacccagc

541 cttaatagcg tcgcgaacgt cctgagaatg caaagtgact atcgtgcgcg tgcacccgtg

601 ccgcatcctc actctgcgtg caagcccggc ttcccgggcg cgccagaagg agcgcagcca

661 aaccaggatg atgtttgatg gggtatttga gcacttgcaa cccttatccg gaagccccct

721 ggcccacaaa ggctaggcgc caatgcaagc agttcgcatg cagcccctgg agcggtgccc

781 tcctgataaa ccggccaggg ggcctatgtt ctttactttt ttacaagaga agtcactcaa

841 catcttaaaa tggccgccgt cattgccaag tcctccgtct ccgcggccgt ggcccgcccg

901 gcccgctcca gcgtgcgccc catggccgcg ctgaagcccg ccgtcaaggc cgcccccgtg

961 gctgccccgg ctcaggccaa ccagatgatg gtctggaccc cggtcaacaa caagtgagtc

1021 gacgagcaag cccggcggat caggcagcgt gcttgcagat ttgacttgca acgcccgcat

1081 tgtgtcgacg aaggcttttg gctcctctgt cgctgtctca agcagcatct aaccctgcgt

1141 cgccgtttcc atttgcagga tgttcgagac cttctcctac ctgccccccc tgagcgacga

1201 gcagatcgcc gcccaggtcg actacattgt cgccaacggt gagcttgcgg ggttgcgagc

1261 aacactccag caacgaacag tgcccaagtc aggaatctgc agtcagcctg ggctttcggc

1321 ggctttttct tgggcaaaca gcttgcactc atgccagcgc ggcttgtcca gcctcacttg

1381 agctttccag ctgctaccag ccgggctata cgacagcgac agagccatag cgtggaatca

1441 cttatttggg ttgccgaagt agcggtcgga gcgtgagttc ttggtcaagc cgccccttat

1501 ccggttcctg tccgtgtctt tgtccctcgt tcacccttcg cggcaccctt catccccttg

1561 cttgcaggct ggatcccctg cctggagttc gctgagtcgg acaaggccta cgtgtccaac

1621 gagtcggcca tccgcttcgg cagcgtgtct tgcgtaagtc tggcgagagc ccgacgggtc

1681 cactgtggca ctgggttagc ttttggcaca cgggtccact gtggcactgg ttagcttggc

1741 accgggacag cgcctatctc accgcgggga actgacgcat acccctgctc gtgcttcagc

1801 acggaaaagc aaggggccca attccatctt tggtggttct gtgcgctggt gactgaacct

1861 cttctccctc ccatttcccg tgcgcccgca gctgtactac gacaaccgct actggaccat

1921 gtggaagctg cccatgttcg gctgccgcga ccccatgcag gtgctgcgcg agatcgtcgc

1981 ctgcaccaag gccttccccg atgcctacgt gcgcctggtg gccttcgaca accagaagca

2041 ggtgcagatc atgggcttcc tggtccagcg ccccaagtct gcccgcgact ggcagcccgc

2101 caacaagcgc tccgtgggca gcggcagcgg cagcctgctg gccatccacc ccaccgaggc

2161 ccgccacaag cagaagatcg tggcccccgt gaagcagacc ctgaacttcg acctgctgaa

2221 gctggccggc gacgtggaga gcaaccccgg ccccggcagc ggcagcggca gcgccgagat

2281 gttcaacggc aacagcagca acgacggcag cagctgcatg cccgtgaagg acgccctgcg

2341 ccgcaccggc aaccaccacc ccaacctgtg gtgagtcgac gagcaagccc ggcggatcag

2401 gcagcgtgct tgcagatttg acttgcaacg cccgcattgt gtcgacgaag gcttttggct

2461 cctctgtcgc tgtctcaagc agcatctaac cctgcgtcgc cgtttccatt tgcaggaccg

2521 acgacttcat ccagagcctg aacagcccct acagcgacag cagctaccac aagcaccgcg

2581 agatcctgat cgacgagatc cgcgacatgt tcagcaacgg cgagggcgac gagttcggcg

2641 tgctggagaa catctggttc gtggacgtgg tgcagcgcct gggcatcgac cgccacttcc

2701 aggaggagat caagaccgcc ctggactaca tctacaagtt ctggaaccac gacagcatct

2761 tcggcgacct gaacatggtg gccctgggct tccgcatcct gcgcctgaac cgctacgtgg

2821 ccagcagcga cgtgttcaag aagttcaagg gcgaggaggg ccagttcagc ggcttcgaga

2881 gcagcgacca ggacgccaag ctggtgagtc gacgagcaag cccggcggat caggcagcgt

2941 gcttgcagat ttgacttgca acgcccgcat tgtgtcgacg aaggcttttg gctcctctgt

3001 cgctgtctca agcagcatct aaccctgcgt cgccgtttcc atttgcagga gatgatgctg

3061 aacctgtaca aggccagcga gctggacttc cccgacgagg acatcctgaa ggaggcccgc

3121 gccttcgcca gcatgtacct gaagcacgtg atcaaggagt acggcgacat ccaggagagc

3181 aagaaccccc tgctgatgga gatcgagtac accttcaagt acccctggcg ctgccgcctg

3241 ccccgcctgg aggcctggaa cttcatccac atcatgcgcc agcaggactg caacatcagc

3301 ctggccaaca acctgtacaa gatccccaag atttacatga agaagatcct ggagctggcc

3361 atcctggact tcaacatcct gcagagccag caccagcacg agatgaagct gatcagcacc

3421 tggtggaaga acagcagcgc catccagctg gtgagtcgac gagcaagccc ggcggatcag

3481 gcagcgtgct tgcagatttg acttgcaacg cccgcattgt gtcgacgaag gcttttggct

3541 cctctgtcgc tgtctcaagc agcatctaac cctgcgtcgc cgtttccatt tgcaggactt

3601 cttccgccac cgccacatcg agagctactt ctggtgggcc agccccctgt tcgagcccga

3661 gttcagcacc tgccgcatca actgcaccaa gctgagcacc aagatgttcc tgctggacga

3721 catctacgac acctacggca ccgtggagga gctgaagccc ttcaccacca ccctgacccg

3781 ctgggacgtg agcaccgtgg acaaccaccc cgactacatg aagatcgcct tcaacttcag

3841 ctacgagatt tacaaggaga tcgccagcga ggccgagcgc aagcacggcc ccttcgtgta

3901 caagtacctg cagagctgct ggaagagcta catcgaggcc tacatgcagg aggccgagtg

3961 gatcgccagc aaccacatcc ccggcttcga cgagtacctg atgaacggtg agtcgacgag

4021 caagcccggc ggatcaggca gcgtgcttgc agatttgact tgcaacgccc gcattgtgtc

4081 gacgaaggct tttggctcct ctgtcgctgt ctcaagcagc atctaaccct gcgtcgccgt

4141 ttccatttgc aggcgtgaag agcagcggca tgcgcatcct gatgatccac gccctgatcc

4201 tgatggacac ccccctgagc gacgagatcc tggagcagct ggacatcccc agcagcaaga

4261 gccaggccct gctgagcctg atcacccgcc tggtggacga cgtgaaggac ttcgaggacg

4321 agcaggccca cggcgagatg gccagcagca tcgagtgcta catgaaggac aaccacggca

4381 gcacccgcga ggacgccctg aactacctga agatccgcat cgagagctgc gtgcaggagc

4441 tgaacaagga gctgctggag cccagcaaca tgcacggcag cttccgcaac ctgtacctga

4501 acgtgggcat gcgcgtgatc ttcttcatgc tgaacgacgg tgagtcgacg agcaagcccg

4561 gcggatcagg cagcgtgctt gcagatttga cttgcaacgc ccgcattgtg tcgacgaagg

4621 cttttggctc ctctgtcgct gtctcaagca gcatctaacc ctgcgtcgcc gtttccattt

4681 gcaggcgacc tgttcaccca cagcaaccgc aaggagatcc aggacgccat caccaagttc

4741 ttcgtggagc ccatcatccc cggcagcggc agcggcagcc gcgaggccgt gatcgccgag

4801 gtgagcaccc agctgagcga ggtggtgggc gtgatcgagc gccacctgga gcccaccctg

4861 ctggccgtgc acctgtacgg cagcgccgtg gacggcggcc tgaagcccca cagcgacatc

4921 gacctgctgg tgaccgtgac cgtgcgcctg gacgagacga cccgccgcgc cctgatcaac

4981 gacctgctgg agacgagcgc cagccccggc gagagcgaga tcctgcgcgc cgtggaggtg

5041 agtcgacgag caagcccggc ggatcaggca gcgtgcttgc agatttgact tgcaacgccc

5101 gcattgtgtc gacgaaggct tttggctcct ctgtcgctgt ctcaagcagc atctaaccct

5161 gcgtcgccgt ttccatttgc aggtgaccat cgtggtgcac gacgacatca tcccctggcg

5221 ctaccccgcc aagcgcgagc tgcagttcgg cgagtggcag cgcaacgaca tcctggccgg

5281 catcttcgag cccgccacca tcgacatcga cctggccatc ctgctgacca aggcccgcga

5341 gcacagcgtg gccctggtgg gccccgccgc cgaggagctg ttcgaccccg tgcccgagca

5401 ggacctgttc gaggccctga acgagacgct gaccctgtgg aacagccccc ccgactgggc

5461 cggcgacgag cgcaacgtgg tgctgaccct gagccgcatc tggtacagcg ccgtgaccgg

5521 caagatcgcc cccaaggtga gtcgacgagc aagcccggcg gatcaggcag cgtgcttgca

5581 gatttgactt gcaacgcccg cattgtgtcg acgaaggctt ttggctcctc tgtcgctgtc

5641 tcaagcagca tctaaccctg cgtcgccgtt tccatttgca ggacgtggcc gccgactggg

5701 ccatggagcg cctgcccgcc cagtaccagc ccgtgatcct ggaggcccgc caggcctacc

5761 tgggccagga ggaggaccgc ctggccagcc gcgccgacca gctggaggag ttcgtgcact

5821 acgtgaaggg cgagatcacc aaggtggtgg gcaagtaaat ggaggcgctc gttgatctga

5881 gccttgcccc ctgacgaacg gcggtggatg gaagatactg ctctcaagtg ctgaagcggt

5941 agcttagctc cccgtttcgt gctgatcagt ctttttcaac acgtaaaaag cggaggagtt

6001 ttgcaatttt gttggttgta acgatcctcc gttgattttg gcctctttct ccatgggcgg

6061 gctgggcgta tttgaagcgc ttttggaaaa gttgctgcgg ggttcatcag ctgaagggga

6121 ctcggttcgc agatcagtta cacactaaag aacggcgggt agcaacacca gcaaacgtga

6181 cgaaacggaa ccgtgcagca aaggtggaga cagcatttgc agtaacctgc agtgacgaac

6241 atgagtcagt tgttcccggt gcccctttgc ttaatctgca tagaatgata gccagctacc

6301 tagtgccaac gtcgataggg ggcgagattg ggtgtacaaa acgtacattg gtgagtaaag

6361 gccttacttg gggtcctccg cgctggcgct ggcgcgtgtt ccgcacctgt tctaaacgat

6421 gcaaaccttt gattctgccc cgctgcccac ttcctgcagc tcccaacccc gcctcgtatg

6481 atgccgttcc ggcattgtgt ccttgatgac cgtgctgtat ggtacaagct gtgcccatga

6541 ctgcagcttc tacgctgcag tgcatcacgc ctcctgtccc tccctccctc ccttacatgt

6601 cgtgctgggc accggtggcg ctggtgttct ccaggttggt ttcgggcgca tcctttctgg

6661 tagtcccaac gccagcccgg ccggcgtcat ccagcccagc catcccaata ccgcagccag

6721 cttccgtcag ccag

//
